# Discovery of a Novel Class of Acylthiourea-Containing Isoxazoline Insecticides against *Plutella xylostella*

**DOI:** 10.3390/molecules28083300

**Published:** 2023-04-07

**Authors:** Fangyi Li, Biaobiao Jiang, Yuqin Luo, Siqi He, Di Feng, Deyu Hu, Runjiang Song

**Affiliations:** National Key Laboratory of Green Pesticide, Key Laboratory of Green Pesticide and Agricultural Bioengineering, Ministry of Education, Center for R&D of Fine Chemicals of Guizhou University, Guiyang 550025, China; gs.fangyili20@gzu.edu.cn (F.L.); jiangbb_gz@foxmail.com (B.J.); luoyuqin2019@163.com (Y.L.); fengdi15286592722@163.com (D.F.)

**Keywords:** diamondback moth, isoxazoline, acylthiourea, 3D-QSAR, insecticidal activity, molecular docking, proteomics

## Abstract

Isoxazoline structures are widely found in natural products and are rich in biological activities. This study discloses the development of a series of novel isoxazoline derivatives by introducing acylthiourea fragments to access insecticidal activity. All synthetic compounds were examined for their insecticidal activity against *Plutella xylostella*, with results showing moderate to strong activity. Based on this, the structure–activity relationship analysis was carried out via the constructed three-dimensional quantitative structure–activity relationship model to further guide the structure optimization, resulting in the optimal compound **32**. The LC_50_ of compound **32** against *Plutella xylostella* was 0.26 mg/L, demonstrating better activity than the positive control, ethiprole (LC_50_ = 3.81 mg/L), avermectin (LC_50_ = 12.32 mg/L), and compounds **1**–**31**. The insect GABA enzyme-linked immunosorbent assay demonstrated that compound **32** might act on the insect GABA receptor, and the molecular docking assay further illustrated the mode of action of compound **32** with the GABA receptor. In addition, the proteomics analysis indicated that the action of compound **32** on *Plutella xylostella* was multi-pathway.

## 1. Introduction

Vegetables are an indispensable side dish for human beings. Not only do they offer a diet with diversity, but they also provide essential nutrients for our health, such as dietary fiber, vitamins, and minerals. Many of the veggies on which people depend are members of the crucifer family, and the diamondback moth (*Plutella xylostella*) is one of the most destructive multigenerational insect pests for them worldwide, producing between four and twenty generations per year in temperate and tropical regions, respectively [1,2]. The challenge of managing this moth is exacerbated by its strong reproductive potential, rapid generation turnover, protracted growing period, and extensive migration range [3]. The rapid evolution of drug resistance owing to the irrational use of insecticides has made its control a major concern for many countries, yet there is a dearth of effective agrochemicals on the market to combat it [4,5]. It has been found that the moth is highly resistant to organophosphorus, carbamates, pyrethroids, novel chemical insecticides, and toxins from Bacillus thuringiensis at the present time [1,6,7]. Breeding to accomplish control of this pest is less likely, given that *Plutella xylostella* is the first insect identified to be resistant to the biocide *Bacillus thuringiensis* [8]. Natural enemies, such as parasitic wasps and other organisms, appear to be a more viable option for population control; nonetheless, the high cost of biological control and the uncertainty factor significantly limit its deployment in the field. Therefore, new insecticides with unique modes of action against diamondback moths are desperately needed.

The discovery of novel effective insecticides from active fragments present in natural products has certainly brought better options for the control of *Plutella xylostella*. There is no doubt that avermectin, derived from Streptomyces avermitilis, is a successful example. Avermectin inhibits the potential conduction of the nervous system by increasing the release of GABA (γ-aminobutyric acid), which eventually causes paralysis and death of the insect [9]. The GABA receptor is necessary for the fast inhibitory neurotransmission of insects [10] and, thus, is an important target for insecticides. By blocking GABA-gated chloride channels in their normal state, one class of insecticides known as “GABA receptor antagonists” is able to achieve its intended effect of killing insects by disrupting their regular behavior [11,12,13]. Isoxazoline compounds are a novel class of pesticides acting on this target and can bind to new sites on known insecticide targets without cross-resistance to other conventional insecticide types [14,15,16]. In addition to being easily accessible in natural goods [17], this active moiety has been shown to be promising against agricultural pests such as Hemiptera, Thysanoptera, Diptera, Lepidoptera, and mites [18,19]. More importantly, GABA receptors are more selective in insects than in mammals [20]. Such facts suggest that isoxazoline scaffolds may provide new ideas for the design of insecticide compounds. Therefore, the design of isoxazoline derivatives for the creation of agricultural insecticides has great research value and significance; it is expected that there will be broad application prospects.

**Figure 1:**
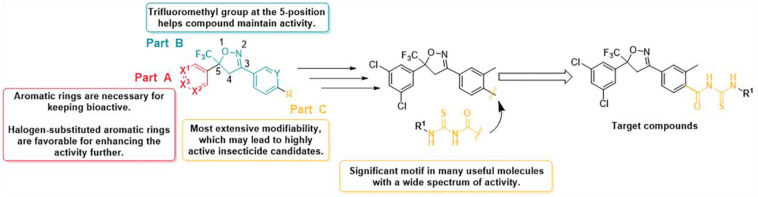
Design of the target compounds.

Based on the structure of isoxazolines, modifications to it can occur mainly in three regions (Figure 1). To keep its insecticidal action, component A of the molecule requires an aromatic ring substitution at the 5-position of isoxazoline, and the installation of halogen at the aromatic ring substituent can further improve the insecticidal effect [21,22,23]. The trifluoromethyl group at the 5-position of isoxazoline in part B is similarly crucial to help the compound remain active [24]. Major R&D institutions have concentrated on optimizing the C region since it is the most amenable to change and may produce highly active pesticide candidates.

The acylthiourea backbone plays a significant motif in the construction of many useful compounds, and its derivatives have demonstrated excellent biological properties, including insecticidal [25,26], fungicidal [27,28], herbicidal [29,30], antitumor [31,32], and other activities, garnering considerable interest in recent years. In 2020, Lu et al. [25] combined the acylthiourea moiety with the natural product source insecticide doramectin and found compounds that were more effective than doramectin and avermectin for the control of *Plutella xylostella*.

**Figure 2:**
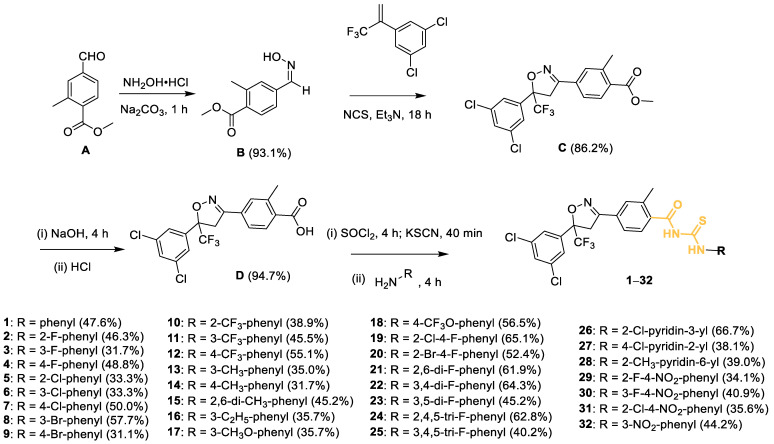
Synthesis route of the target compounds **1**–**32**.

In the present study, a new series of acylthiourea-containing isoxazoline derivatives were synthesized by introducing acylthiourea moieties into part C of the isoxazolines, and their synthetic pathways are shown in Figure 2. We evaluated the pesticidal activity of these compounds against *Plutella xylostella.* On this basis, a three-dimensional quantitative structure–activity relationship (3D-QSAR) model was constructed to obtain the optimal compound **32** with the best insecticidal activity. In addition, a mechanism study concerning the action mode of compound **32** was carried out by enzyme activity assay, molecular docking assay, and proteomics assay. According to the insect GABA enzyme-linked immunosorbent assay (ELISA), compound **32** may act on the insect GABA receptor, and molecular docking explains the key factors for the interaction of compound **32** with the receptor.

## 2. Results and Discussion

### 2.1. Preparation of Compounds

Using methyl 4-formyl-2-methylbenzoate as the starting material, compound **B** was obtained by nucleophilic addition–elimination reaction with hydroxylamine hydrochloride, followed by 1,3-dipole cycloaddition reaction with NCS and 1,3-dichloro-5-(3,3,3-trifluoroprop-1-en-2-yl)benzene to give isoxazoline intermediate **C**. Compound **C** was subjected to hydrolysis reaction to give intermediate **D**. Intermediate **D** was reacted with KSCN under sulfoxide chloride conditions to form acyl isothiocyanates, and finally with various substituted amines via nucleophilic reactions to produce compounds **1**–**32**. The data of all target compounds (melting point, yield, ^1^H NMR, ^13^C NMR, and HRMS) are given in the Supplementary Material.

### 2.2. Insecticidal Activity

Table 1 shows the pesticidal activities of the target compounds against diamondback moths. Obviously, most of the compounds showed potent insecticidal activities against diamondback moths. All compounds showed 100% mortality at 100 mg/L, and at a concentration of 10 mg/L, half of the compounds still showed more than 80% mortality. The LC_50_ of compounds **1**–**3**, **7**, **9**, **11**, **21**–**23**, **29**, and **32** were 2.53, 3.12, 1.62, 0.51, 2.53, 3.50, 2.55, 0.89, 2.11, 1.02, and 0.26 mg/L, respectively, which were all the better than the positive control ethiprole (3.81 mg/L) and avermectin (LC_50_ = 12.32 mg/L).

### 2.3. 3D-QSAR Analysis

**Figure 3:**
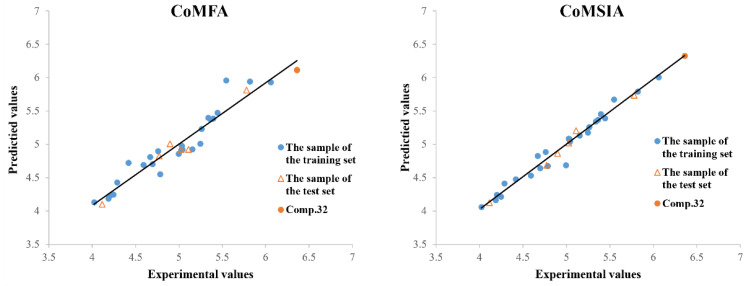
Plots of experimental and predicted pLC_50_ for the CoMFA and CoMSIA models of 3D-QSAR.

The comparative molecular field analysis (CoMFA) model and comparative molecular similarity index analysis (CoMSIA) model were established using the pLC_50_ values of the target compounds that had been synthesized against *Plutella xylostella*. As shown in Table 2, the cross-validation coefficient (*q*^2^) of the constructed CoMFA model is 0.751, and the non-cross-validation correlation coefficient (*r*^2^) is 0.931. The *q*^2^ of the CoMSIA model is 0.697, and the *r*^2^ is 0.977 (*q*^2^ > 0.5 and *r*^2^ > 0.8). As shown in Figure 3 and Table 3, the experimental and predicted values of molecular activity in the training and test sets were highly consistent, which shows that the model has good stability and is reliable. In the CoMFA model, the relative contributions of the steric field and electrostatic field to the model were 52.7% and 47.3%. The results showed that the spatial effect has a slightly stronger effect on the activity than the electrical distribution of the groups. The steric field, electrostatic field, hydrophobic field, H-bond acceptor field, and H-bond donor field in the CoMSIA model all have an influence on the activity of the compound, and the relative contributions are 9.5%, 29.1%, 22.6%, 29.1%, and 9.7%, respectively, which shows that the electrostatic field, hydrophobic field, and H-bond acceptor field have greater effects on the compound molecular activity.

**Figure 4:**
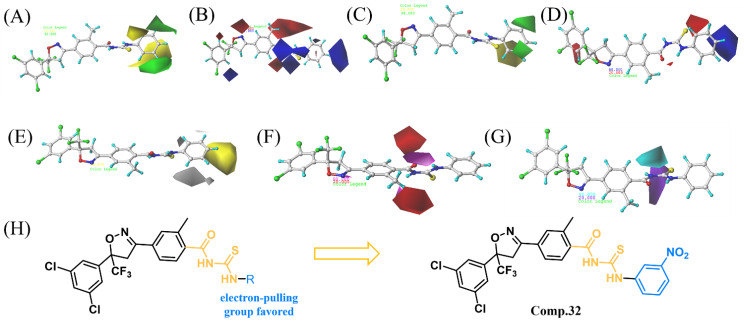
CoMFA contour maps of steric field (**A**) and electrostatic field (**B**). CoMSIA contour maps of steric field (**C**), electrostatic field (**D**), hydrophobic field (**E**), H-bond acceptor field (**F**), and H-bond donor field (**G**). Relationship between the structure and insecticidal activity against *Plutella xylostella* (**H**).

Figure 4A demonstrates the contour map of the steric field of CoMFA, where the green region indicates that increasing the substituent space structure can improve the activity of the compound, and the yellow region is counterproductive. As shown in Figure 4A, the yellow color block covering near the 2-position of the R group demonstrates that the introduction of smaller groups can improve the insecticidal activity, for example, compound **1** (R = phenyl, LC_50_ = 2.53 mg/L) > **2** (R = 2-F-phenyl, LC_50_ = 3.12 mg/L) > **10** (R = 2-CF_3_-phenyl, LC_50_ = 47.51 mg/L). The green color block covering near the 3-position of the R group demonstrates that the insecticidal activity is improved when larger groups are introduced, such as compound **16** (R = 3-C_2_H_5_-phenyl, LC_50_ = 4.07 mg/L) > **13** (R = 3-CH_3_-phenyl, LC_50_ = 29.15 mg/L). The contour map of the electrostatic field of CoMFA is shown in Figure 4B, where the blue area indicates that the addition of positively charged groups can improve the activity of the compounds, and the red area is counterproductive. As shown in Figure 4B, there are blue color blocks covering near the 1- and 4-positions of the R group, which means that the introduction of the electron-giving group is favorable for the activity of this part, which explains the better activity of compound **1** (R = phenyl, LC_50_ = 2.53 mg/L) than compound **4** (R = 4-F-phenyl, LC_50_ = 5.21 mg/L).

The steric and electrostatic field contour maps of the CoMSIA model are shown in Figure 4C,D. From these two field contour maps in the CoMFA model and the CoMSIA model, the locations of the areas covered by the same color are basically the same, which shows that these two fields have common reference suggestions for the structural optimization of such compounds. In the hydrophobic field contour maps of the CoMSIA model (Figure 4E), with a yellow block covering near the 4-position of the R group, indicating that the addition of hydrophobic groups here is beneficial to improve the activity, such as compound **7** (R = 4-Cl-phenyl, LC_50_ = 0.51 mg/L) > **1** (R = phenyl, LC_50_ = 2.53 mg/L) > **27** (R = 4-Cl-pyridine-2-yl, LC_50_ = 37.06 mg/L). Interestingly, in the CoMSIA model of the H-bonded acceptor field and H-bonded donor field (Figure 4F,G), there are purplish red and blue–green contours distributed on the acylthiourea group, which indicates that acylthiourea group has an important influence on the activity of the target compound.

### 2.4. Design and Synthesis of Compound **32**

**Figure 5:**
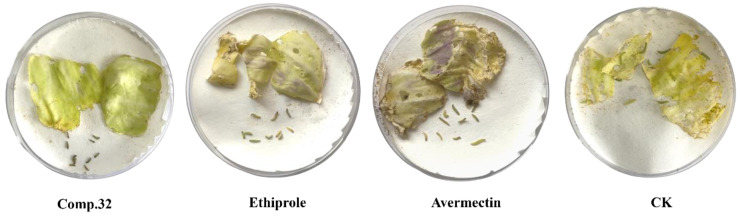
Insecticidal activity of the compounds against *Plutella xylostella* at 10 mg/L (48 h).

On the basis of the constructed 3D-QSAR model, a new candidate compound **32** was synthesized by retaining the acylthiourea part and adding an electron-accepting nitro group at the 3-position of the benzene ring. The insecticidal activity of compound **32** against *Plutella xylostella* was tested (Figure 5), and the experimental results exhibited that the LC_50_ of compound **32** was 0.26 mg/L, which was better than the positive control ethiprole (LC_50_ = 3.81 mg/L), avermectin (LC_50_ = 12.32 mg/L), and compounds **1**–**31**, demonstrating the 3D-QSAR model’s good predictive power.

### 2.5. Enzyme Activity Determination

**Figure 6:**
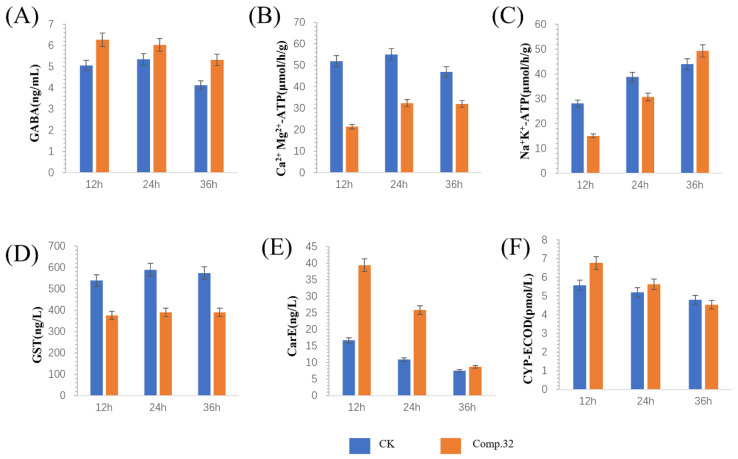
Effect of compound **32** on the enzyme activities of GABA (**A**), Ca^2+^Mg^2+^-ATP (**B**), Na^+^ K^+^ -ATP (**C**), GST (**D**), CarE (**E**), and CYP-ECOD (**F**) of the diamondback moth. Vertical bars indicate mean ± SD (n = 3).

Figure 6 shows the effects of compound **32** on the enzyme activities of GABA, Ca^2+^Mg^2+^-ATPase (Ca^2+^Mg^2+^-ATP), Na^+^K^+^-ATPase (Na^+^K^+^-ATP), glutathione-S-transferase (GST), carboxylesterase (CarE), and cytochrome P450 enzyme (CYP-ECOD) in *Plutella xylostella*. GABA levels were all increased compared to the blank group but decreased with increasing test time, indicating that compound **32** may act on the GABA receptor. The inhibition of Ca^2+^Mg^2+^-ATP activity by compound **32** at 12, 24, and 36 h was 58.8%, 41.2%, and 31.8%, respectively. At 12 and 24 h, the inhibition of Na^+^K^+^-ATP activity was 46.3% and 20.8%. Although compound **32** can down-regulate the GST content, there is no significant change at all time periods, demonstrating that the regulation of compound **32** on GST does not change with the transformation of time. Interestingly, the activities of CarE and CYP-ECOD detoxification enzymes showed a trend of first high to low, which was consistent with the trend of GABA content, indicating that the detoxification capacity decreased with time. Notably, the most significant increase in CarE enzyme activity was observed, with the strongest detoxification capacity at 12 h.

### 2.6. Docking

**Figure 7:**
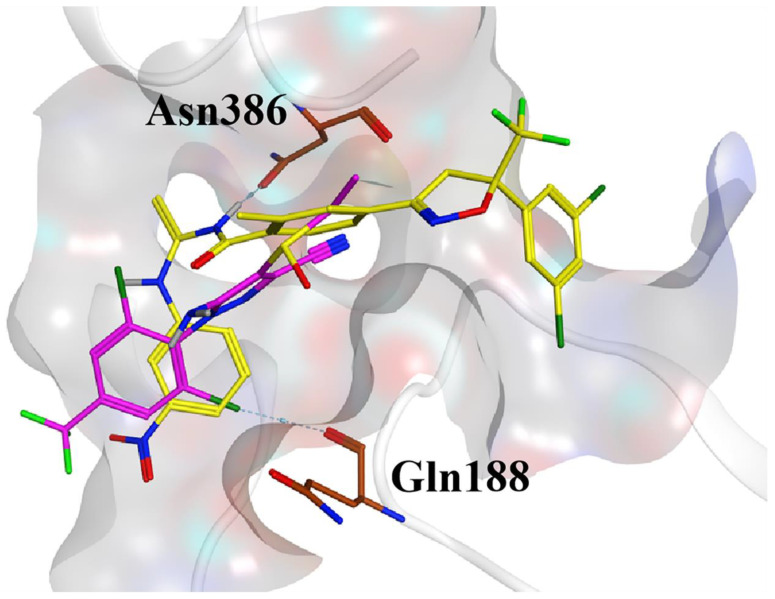
Predicted binding pattern of compound **32** (yellow) and ethiprole (pink) at the GABA-active site.

To explore the mode of action of the compounds with the GABA receptor, molecular docking experiments were performed. The results showed that compound **32** and ethiprole had similar binding patterns within the RDL active site. Compound **32** formed a hydrogen bond interaction with the nitrogen atom on the amide bond. The chlorine atom on the benzene ring of the positive control ethiprole formed a hydrogen bond interaction with Gln188 (Figure 7). The presence of these interactions played a stabilizing role in the binding of the GABA receptor to compound **32** and ethiprole. Compound **32** occupied more active cavities than ethiprole, which may account for its greater effectiveness than ethiprole against *Plutella xylostella*.

### 2.7. Proteomics Analysis

**Figure 8:**
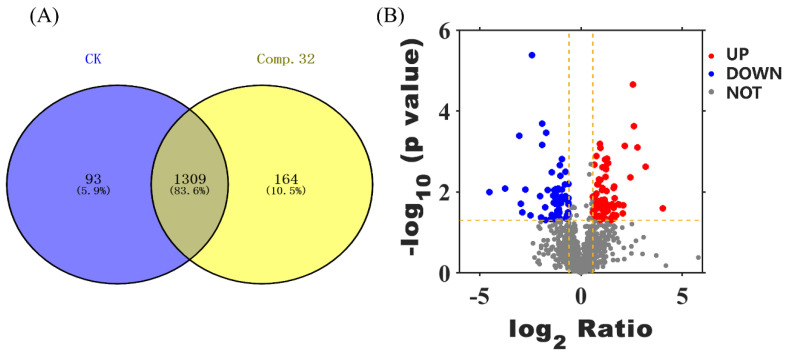
Venn diagram (**A**) and volcano plot (**B**) for proteins identified in the treatment and control groups.

To investigate the mode of action of compound **32** on diamondback moths, quantitative proteomics analysis of diamondback moths was carried out using a protein profiling label-free technique. As shown in Figure 8A, a total of 1566 proteins were identified, including 1402 proteins in the CK group and 1473 proteins in the treatment group. The differential proteins were 93 (5.9%) in the control group, 164 (10.5%) in the treated group, and 1309 (83.6%) identical proteins in both groups.

#### 2.7.1. GO Analysis

**Figure 9:**
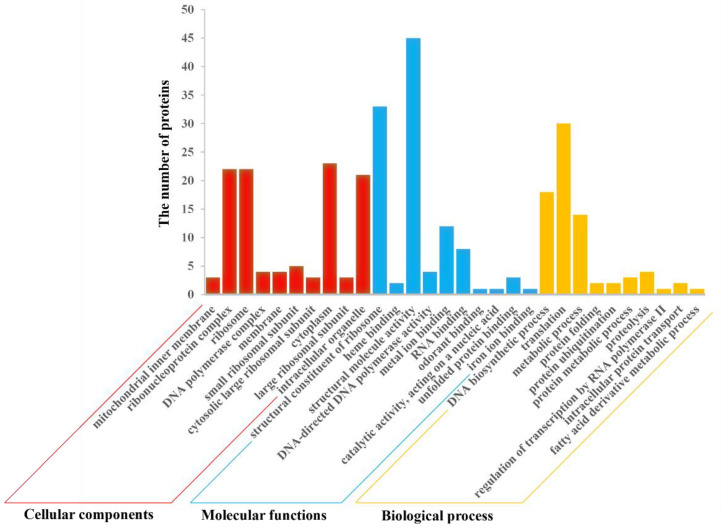
Differential expressed proteins classified based on known cellular components, biological processes, and molecular functions.

The results of the GO functional enrichment analysis of the differential proteins are shown in Figure 9. In the cellular components, the DEPs were mainly enriched in the mitochondrial inner membrane, ribonucleoprotein complex, ribosome, DNA polymerase complex, membrane, small ribosomal subunit, cytosolic large ribosomal subunit, cytoplasm, large ribosomal subunit, and intracellular organelle. The molecular functions were mainly enriched in a structural constituent of ribosome, heme binding, structural molecule activity, DNA-directed DNA polymerase activity, metal–ion binding, RNA binding, odorant binding, catalytic activity, acting on nucleic acid, unfolded protein binding, and iron–ion binding. The biological processes involved were mainly enriched in DNA biosynthetic process, translation, metabolic process, protein folding, protein ubiquitination, protein metabolic process, proteolysis, regulation of transcription by RNA polymerase II, intracellular protein transport, and fatty acid derivative metabolic process.

#### 2.7.2. KEGG Classification Analysis

KEGG functional enrichment results showed that differential proteins in the ribosome, fatty acid metabolism, drug metabolism–other enzymes, oxidative phosphorylation, drug metabolism–cytochrome P450, metabolism of xenobiotics by cytochrome P450, glutathione metabolism, biosynthesis of amino acids, glycolysis/gluconeogenesis, phagosome (top 10) were significantly enriched in these pathways. These pathways play important roles in insect development, detoxification, and metabolism, suggesting that compound **32** can act by regulating a variety of biological processes. As shown in Table 4, a total of three proteins were enriched to the oxidative phosphorylation pathway in the treated and control groups, in which mitochondrial cytochrome C protein expression was reduced, vacuolar ATP synthethase subunit e and ATP synthase subunit d protein expression were unchanged. Cytochrome C is an essential part of the mitochondrial respiratory chain, which is generated from two inactive precursor molecules, procytochrome C, and heme [33]. Since cytochrome C has a ferrous heme group, it can transfer electrons between respiratory chain complex enzyme III (cytochrome reductase) and respiratory chain complex enzyme IV (cytochrome oxidase) [34,35]. When cytochrome C is deficient in insects, the electron transport chain is blocked, ATP synthesis is reduced, and reactive oxygen species (ROS) accumulate excessively due to incomplete oxidation, thus affecting the oxidative phosphorylation process [36,37]. Thus, we speculate that compound **32**, by down-regulating the expression of mitochondrial cytochrome C protein, prevents normal electron transport of the respiratory chain and the accumulation of ROS, which eventually causes iron death [38]. On the other hand, cytochrome C is a key substance for mitochondria to initiate the apoptotic program [39], and apoptosis plays a vital role in maintaining the normal physiological homeostasis of the organism and removing senescent cells from the organism [40]. Reduced expression of mitochondrial cytochrome C protein inhibited the apoptotic effect and affected the normal growth and development of *Plutella xylostella*. Taken together, compound **32** may act on the GABA receptor of *Plutella xylostella*, thus releasing the inhibitory neurotransmitter GABA, which transmits the respiratory inhibition signals and eventually leads to the death of *Plutella xylostella* due to respiratory inhibition.

## 3. Experimental

### 3.1. Instruments and Chemicals

All solvents and reagents used in these experiments were purchased from domestic suppliers and used directly without further purification. Melting point data of the compounds were measured by Shanghai Yice WRX-4 melting point instrument and the temperature without correction. The yields were not optimized. The ^1^H NMR and ^13^C NMR of the compounds were recorded using an ECX-500 (JEOL, Tokyo, Japan) or an Ascend-400 spectrometer (Bruker, Billerica, MA, USA), tetramethylsilane (TMS) as the internal standard, and deuterated chloroform (CDCl_3_) or deuterated dimethyl sulfoxide (DMSO-*d_6_*) as the solvent. High-resolution mass spectrometry (HRMS) data of the target compounds were obtained from Thermo Scientific Q Exactive (Thermo, Waltham, MA, USA).

### 3.2. Synthesis

#### 3.2.1. Synthesis of Intermediate B

At room temperature, methyl-4-formyl-2-methylbenzoate **A** (56.16 mmol), hydroxylamine hydrochloride (67.39 mmol), and anhydrous ethanol (30 mL) were added to the reaction flask. Then, the system was adjusted to neutral with saturated Na_2_CO_3_ solution, and the reaction process was followed by TLC. Ethyl acetate was added to the system and then extracted; the organic layer was collected and recrystallized with anhydrous ethanol to obtain compound **B**.

#### 3.2.2. Synthesis of Intermediate C

Compound **B** (25.88 mmol), *N*-chlorosuccinimide (38.82 mmol), and DMF (30 mL) were added to a three-necked flask. The system was stirred at 40 °C for 40 min, then cooled to room temperature, and 1,3-dichloro-5-(3,3,3-trifluoroprop-1-en-2-yl)benzene (31.06 mmol) and Et_3_N (38.82 mmol) were added under ice bath conditions. Monitoring of reaction completion by TLC, ethyl acetate was added to the crude products and then extracted, and the organic layer was collected. Intermediate **C** was obtained after purification by column chromatography (petroleum ether: ethyl acetate = 5:1 (*v*/*v*)).

#### 3.2.3. Preparation of Intermediates D

An aqueous solution of 4 mol/L NaOH (69.41 mmol) was added to a reaction flask containing compound **C** (23.14 mmol) and ethanol (30 mL), and the system was stirred at 80 °C. The completion of the system was followed by TLC and then concentrated. The concentrated mixture was added to ice water (50 mL) and then acidified with concentrated hydrochloric acid to pH 3–4. Intermediate **D** was obtained by filtration and drying the filter cake.

#### 3.2.4. Preparation of Target Compounds **1**–**32**

Compound **D** (0.72 mmol), dichlorosulfoxide (21.52 mmol), and two drops of DMF were added to the flask, and the system was heated and stirred at 50 °C for 4 h. The mixture was dried by distillation under reduced pressure and dissolved in a solution of dry acetonitrile (5 mL), and set aside. KSCN (2.23 mmol) was added to the mixture of dry acetonitrile (15 mL), two drops of PEG-400 were added and stirred at room temperature for 5 min to homogenize the mixture; then, the above solution was added dropwise. After stirring for 40 min at room temperature, substituted aniline (1.08 mmol) was added, and the crude product was extracted with ethyl acetate after stirring for 3–4 h at room temperature, and the organic layer was collected and purified by column chromatography (petroleum ether: ethyl acetate = 3:1 (*v*/*v*)) to obtain the target compounds **1**–**32**. The title compound **1** data are as shown:

*4-(5-(3,5-dichlorophenyl)-5-(trifluoromethyl)-4,5-dihydroisoxazol-3-yl)-2-methyl-N-(phenylcarbamothioyl)benzamide(**1**)*. Yellow solid; yield: 47.6%; m.p. 93.9–95.8 °C; ^1^H NMR (400 MHz, DMSO-*d*_6_) *δ* 10.41 (s, 1H, -NH-), 7.82 (t, *J* = 1.8 Hz, 1H, Ar-H), 7.74 (d, *J* = 7.8 Hz, 2H, Ar-H), 7.66 (dd, *J* = 11.1, 5.0 Hz, 4H, Ar-H), 7.59 (d, *J* = 7.8 Hz, 1H, Ar-H), 7.35 (t, *J* = 7.9 Hz, 2H, Ar-H), 7.11 (t, *J* = 7.4 Hz, 1H, Ar-H), 4.37 (q, *J* = 18.4 Hz, 2H, isoxazoline-H), 2.43 (s, 3H, -CH_3_). ^13^C NMR (101 MHz, DMSO-*d*_6_) *δ* 167.44, 157.93, 139.87, 139.50, 139.14, 136.60, 135.13, 130.06, 129.73, 129.19, 128.70, 128.34, 126.24, 124.85, 124.22, 120.21, 87.25, 86.96, 43.35, 19.67. HRMS (ESI) m/z [M-H]^-^ calcd for C_25_H_17_Cl_2_F_3_N_3_O_2_S: 550.0365, found: 550.0377.

### 3.3. Insecticidal Activity Test

The biological activity of the target compounds was determined against the second instar larvae of the diamondback moths by the leaf-dip method [41,42]. The procedure was as follows: appropriate amounts of DMSO were used to dissolve the target compound, and a certain amount of 0.05% (*w*/*v*) Triton X-100 buffer solution was added to prepare a master solution of 5000 mg/L; finally, the prepared master mixes were serially diluted to different concentrations with buffer solution. Kale leaves were immersed in solutions with different insecticide concentrations for 15 seconds; blank control leaves were treated with 0.05% Triton X-100 and DMSO solutions, and positive controls were ethiprole and avermectin. Treated leaves were dried at room temperature for 2–3 h and placed in a petri dish lined with filter paper. Each group of concentrations was repeated three times with ten-second instar larvae in each replicate. Finally, the petri dishes were kept at 26 °C, 85% RH (relative humidity), and in the 8 h:16 h (dark: light) incubator. Larvae were observed and recorded for mortality by lightly touching them with a fine brush at 48 h of treatment, and failure to crawl normally was considered death. The larval mortality rate was calculated as follows:

(1) $$\mathrm{Corrected}\;\mathrm{mortality}\;\mathrm{rate}\;\left(\%\right)=\frac{\left(T-C\right)\times 100}{\left(100\%-C\right)}$$

T was the mortality rate of the tested compound group, and C was the mortality rate of the blank control group (both T and C were expressed as percentages). Relevant parameters were calculated using SPSS 25.0 software (IBM, Inc., Armonk, NY, USA).

### 3.4. 3D-QSAR Models

Twenty-five target compounds were selected as the training set, and six as the test set in the absence of a specific case, and a 3D-QSAR model was constructed using SYBYL-X 2.0 software (Tripos, St. Louis, MO, USA). The CoMFA and CoMSIA models were developed, and the predictive power of the models was evaluated. The activity values pLC_50_ used are represented by the (−l g LC_50_) variation. The energy of all compound molecules was minimized and stacked with compound **7** as the template molecule. CoMFA and CoMSIA models were developed using partial least squares (PLS) methods to correlate the insecticidal activity of the target compounds with structural features. To obtain the optimal number of components (ONC) and the *q*^2^, the leave-one-out (LOO) method was used for cross-validation of the training set. The *r*^2^ and the standard error of estimate (SEE) and *F*-value were obtained after non-cross-validation of the training set. Finally, the CoMFA and CoMSIA models were used to anticipate the insecticidal activity of compounds in the test set.

### 3.5. Enzyme Activity Assays

The second instar larvae of the moth were treated with compound **32** (0.26 mg/L) and placed in an artificial climate incubator. The poisoned larvae were collected at 12, 24, and 36 h, respectively, and stored in a −80 °C refrigerator. Finally, enzymatic assay kits were used to detect the activities of GABA, Ca^2+^Mg^2+^-ATP, Na^+^K^+^-ATP, GST, CarE, and CYP-ECOD. All experiments were repeated three times.

### 3.6. Molecular Docking

The amino acid sequence registration number of the GABA receptor RDL subunit of Drosophila was acquired in the database Uniprot (https://www.uniprot.org, accessed on 5 April 2023), and the human α1-β2-γ2 type GABA receptor was selected as the template protein using the Drosophila RDL subunit (Q75NA5) as the target sequence. MOE was used to calculate the optimal protein active sites and constructed and optimized compound structures using MMFF94 force fields and charges. Finally, the lowest-scoring docking conformation was used to further analyze the binding conformation and pattern of the molecule to the receptor.

### 3.7. Proteomics

The test insects in the blank group were diamondback moths continuously reared indoors without exposure to any agent, and the test group was diamondback moths with intoxication after treatment with compound **32** (0.26 mg/ L). The collected test worms were snap-frozen and stored at −80 °C. The test worms were subjected to total protein extraction and mass spectrometry analysis according to the reported methods [43].

## 4. Conclusions

Overall, a series of novel acylthiourea-containing isoxazoline derivatives were generated by introducing the acylthiourea moiety into the C part of the isoxazoline compounds, and these compounds exhibited good insecticidal activity. A 3D-QSRA model was built using the results of the LC_50_ test of the compounds against *Plutella xylostella*, and compound **32** was synthesized on the basis of the optimization proposal of the 3D-QSRA model. Bioassay results showed that compound **32** showed good insecticidal activity against diamondback moths, which was superior to the positive control, ethiprole, avermectin, and compounds **1**–**31**. In addition, insect GABA ELISA showed that compound **32** could up-regulate the GABA content in *Plutella xylostella*, which had the same effect as the insecticide acting on the GABA receptor, indicating that compound **32** might act on the GABA receptor. Moreover, molecular docking assay further illustrated the mode of action of compound **32** with GABA receptor, and all these experimental results were similar to the mode of action of ethiprole. In the proteomics analysis, the differential proteins were mainly enriched in biological processes such as translation of genetic information, drug metabolism, energy metabolism, and protein metabolism, indicating that compound **32** can act by regulating multiple biological processes. In the oxidative phosphorylation pathway, compound **32** can block electron transfer in the respiratory chain of the mitochondrial energy metabolic system of *Plutella xylostella*, ultimately leading to the death of the insect. We believe that this study will provide important reference suggestions for the control of the moths and contribute to the development of new insecticides that are effective against *Plutella xylostella* in the future.

## Figures and Tables

**Table 1:** Insecticidal activity of compounds against the second instar larvae of *Plutella xylostella* (48 h).

Comp.	*Plutella xylostella*			Toxic Regression Equation	*r* ^2^	95% Confidence Interval (mg/L)
	**100 (mg/L) (%)**	**10 (mg/L) (%)**	**LC_50_ (mg/L)**			
**1**	100	100	2.53	y = 3.5287 + 3.6553x	0.95	2.08–3.07
**2**	100	93.3 ± 1.9	3.12	y = 3.5907 + 2.8481x	0.98	2.52–3.88
**3**	100	100	1.62	y = 4.4044 + 2.8452x	0.95	1.26–2.07
**4**	100	86.7 ± 1.9	5.21	y = 2.5900 + 3.3612x	0.98	4.29–6.33
**5**	100	0	22.33	y = 0.0224 + 3.6903x	0.99	18.41–27.07
**6**	100	63.3 ± 5.1	5.43	y = −2.6610 + 3.1839x	0.99	4.46–6.61
**7**	100	100	0.51	y = 6.0002 + 3.4342x	0.97	0.42–0.62
**8**	100	60.0 ± 3.3	10.94	y = 1.5668 + 3.3045x	0.99	8.99–13.31
**9**	100	100	2.53	y = 3.5287 + 3.6553x	0.95	2.08–3.07
**10**	100	0	47.51	y = 0.7868 + 2.5127x	0.94	35.05–64.40
**11**	100	100	3.50	y = 3.3107 + 3.1018x	0.96	2.80–4.39
**12**	100	50.0 ± 3.3	4.79	y = 2.5889 + 3.5447x	0.96	3.94–5.82
**13**	100	43.3 ± 1.9	29.15	y = −2.1821 + 4.9038x	0.94	24.63–34.49
**14**	100	80.0 ± 3.3	9.28	y = 1.6960 + 3.4151x	0.97	7.63–11.28
**15**	100	93.3 ± 1.9	7.31	y = 2.2710 + 3.1592x	0.95	5.79–9.22
**16**	100	60.0 ± 3.3	4.07	y = 3.0852 + 3.1422x	0.96	3.29–5.03
**17**	100	33.3 ± 1.9	11.69	y = 1.2932 + 3.4718x	0.96	9.53–14.33
**18**	100	63.3 ± 3.9	13.63	y = 1.4488 + 3.1302x	0.96	10.98–16.93
**19**	100	3.3 ± 1.9	34.23	y = −1.4041 + 4.1737x	0.97	29.40–39.86
**20**	100	0	42.00	y = −1.0729 + 3.7413x	0.95	35.27–50.01
**21**	100	100	2.55	y = 3.7949 + 2.9582x	0.99	2.10–3.11
**22**	100	100	0.89	y = 5.1782 + 3.4151x	0.97	0.73–1.08
**23**	100	100	2.11	y = 3.9477 + 3.2530x	0.97	1.73–2.57
**24**	100	90.0 ± 3.3	5.70	y = 2.5702 + 3.2151x	0.99	4.67–6.95
**25**	100	83.3 ± 3.9	6.11	y = 2.6955 + 2.9329x	0.92	4.70–7.94
**26**	100	53.3 ± 1.9	9.90	y = 1.3615 + 3.6553x	0.95	8.13–12.04
**27**	100	0	37.06	y = −1.0217 + 3.8381x	0.96	31.55–43.54
**28**	100	90.0 ± 3.3	5.34	y = 2.4222 + 3.5447x	0.96	4.39–6.49
**29**	100	100	1.02	y = 4.9732 + 3.3045x	0.97	0.84–1.24
**30**	100	53.3 ± 1.9	15.77	y = 1.4866 + 2.9329x	0.93	12.25–20.31
**31**	100	0	59.89	y = −11.5058 + 9.2866x	0.99	55.83–64.25
**32**	100	100	0.26	y = 6.7176 + 2.9582x	0.99	0.22–0.32
**Ethiprole**	100	53.3 ± 1.9	3.81	y = −1.0781 + 1.8553x	0.97	2.47–6.06
**Avermectin**	100	30.0 ± 3.3	12.32	y = −4.7237+4.3311x	0.96	9.70–16.24

**Table 2:** Statistical results of the CoMFA and CoMSIA models.

Statistical Parameter	CoMFA	CoMSIA	Verification Standard
*q* ^2a^	0.751	0.697	>0.5
ONC^b^	4	10	
*r* ^2c^	0.931	0.977	>0.8
SEE^d^	0.163	0.106	
*F* ^e^	90.860	90.132	
Fraction of Field Contributions			
steric	0.527	0.095	
electrostatic	0.473	0.291	
hydrophobic		0.226	
hydrogen-bond acceptor		0.291	
hydrogen-bond donor		0.097	

*q*^2a^ = cross-validation correlation coefficient. ONC^b^ = optimum number of principal components. *r*^2c^ = non-cross-validation coefficient. SEE^d^ = standard error of estimate. *F*^e^ = Fisher statistic.

**Table 3:** Experimental and predicted results of pLC_50_ for the CoMFA and CoMSIA models.

Comp.	Experimental (pLC_50_)	CoMFA		CoMSIA	
		Predict *^a^*	Residual *^b^*	Predict *^a^*	Residual *^b^*
**1**	5.338	5.397	0.059	5.344	0.006
**2**	5.260	5.233	−0.027	5.258	−0.002
**3**	5.545	5.957	0.412	5.674	0.129
**4**	5.038	4.923	−0.115	5.043	0.005
**5**	4.418	4.723	0.305	4.479	0.061
**6**	5.032	4.977	−0.055	5.081	0.049
**7**	6.059	5.932	−0.127	6.005	−0.054
**8**	4.759	4.899	0.140	4.887	0.128
**9**	5.395	5.388	−0.007	5.455	0.060
**10** *^c^*	4.114	4.101	−0.013	4.126	0.012
**11**	5.247	5.009	−0.238	5.176	−0.071
**12** *^c^*	5.111	4.924	−0.187	5.205	0.094
**13**	4.287	4.431	0.144	4.413	0.126
**14**	4.784	4.554	−0.230	4.678	−0.106
**15** *^c^*	4.898	5.007	0.109	4.866	−0.032
**16**	5.153	4.927	−0.226	5.131	−0.022
**17**	4.696	4.703	0.007	4.646	−0.050
**18**	4.668	4.812	0.144	4.827	0.159
**19**	4.245	4.247	0.002	4.217	−0.028
**20**	4.187	4.187	−0.000	4.166	−0.021
**21**	5.362	5.379	0.017	5.366	0.004
**22**	5.819	5.943	0.124	5.792	−0.027
**23**	5.444	5.472	0.028	5.391	−0.053
**24**	5.025	4.923	−0.102	5.018	−0.007
**25**	4.995	4.86	−0.135	4.687	−0.308
**26** *^c^*	4.772	4.826	0.054	4.687	−0.085
**27**	4.198	4.224	0.026	4.242	0.044
**28** *^c^*	5.025	4.918	−0.107	5.083	0.058
**29** *^c^*	5.779	5.812	0.033	5.734	−0.045
**30**	4.590	4.693	0.103	4.535	−0.055
**31**	4.021	4.132	0.111	4.06	0.039
**32** *^d^*	6.360	6.113	−0.247	6.348	−0.012

*^a^* Predicted by CoMFA and CoMSIA. *^b^* Residual error (predicted−experimental). *^c^* The sample of the test set. *^d^* Compound was synthesized on the basis of CoMFA and CoMSIA.

**Table 4:** DEPs involved in pathways of oxidative phosphorylation.

Protein ID	Protein Names	Gene Names	Sig
A0A023HN92_PLUXY	Mitochondrial cytochrome C	PLXY2_LOCUS4707	down
Q60FR7_PLUXY	Vacuolar ATP synthethase subunit e		no
D5LN47_PLUXY	ATP synthase subunit d	PLXY2_LOCUS145	no

## Data Availability

All the data in this research were presented in the manuscript and Supplementary Material.

## Supplementary Materials

The following supporting information can be downloaded at: https://www.mdpi.com/article/10.3390/molecules28083300/s1, File S1: ^1^H NMR, ^13^C NMR, and HRMS data of target compounds **1**–**32** are shown in (PDF). File S2: All DEPs are shown in (XLSX). Supplementary data associated with this article can be found in the online version.

## Sample Availability

Samples of the compounds are available from the authors.
