# Supplementary material for: Discovery of a Novel Class of Acylthiourea-Containing Isoxazoline Insecticides against Plutella xylostella
Source: Molecules. 2023 Apr 7;28(8):3300. doi: 10.3390/molecules28083300 (PMC10144074; doi:10.3390/molecules28083300)
Supplement: Supplementary file 1 [file molecules-28-03300-s001.zip › File S1.pdf]

File A

Discovery of a Novel Class of Acylthiourea-Containing Isoxazoline Insecticides Against *Plutella Xylostella*

Fangyi Li[1], Biaobiao Jiang[1], Yuqin Luo[1], Siqi He[1], Di Feng[1], Deyu Hu[1,*] and Runjiang Song[1,*]

[1]National Key Laboratory of Green Pesticide, Key Laboratory of Green Pesticide and Agricultural Bioengineering, Ministry of Education, Center for R&D of Fine Chemicals of Guizhou University, Guiyang 550025, P. R. China.

[*]Author to whom correspondence should be addressed.

Contents

1. Figure S1 1

2. Characterization of target compounds **1-32**. 2

3. ^{1}H NMR, ^{13}C NMR, and HRMS spectrum of the compounds **1-32**. 15

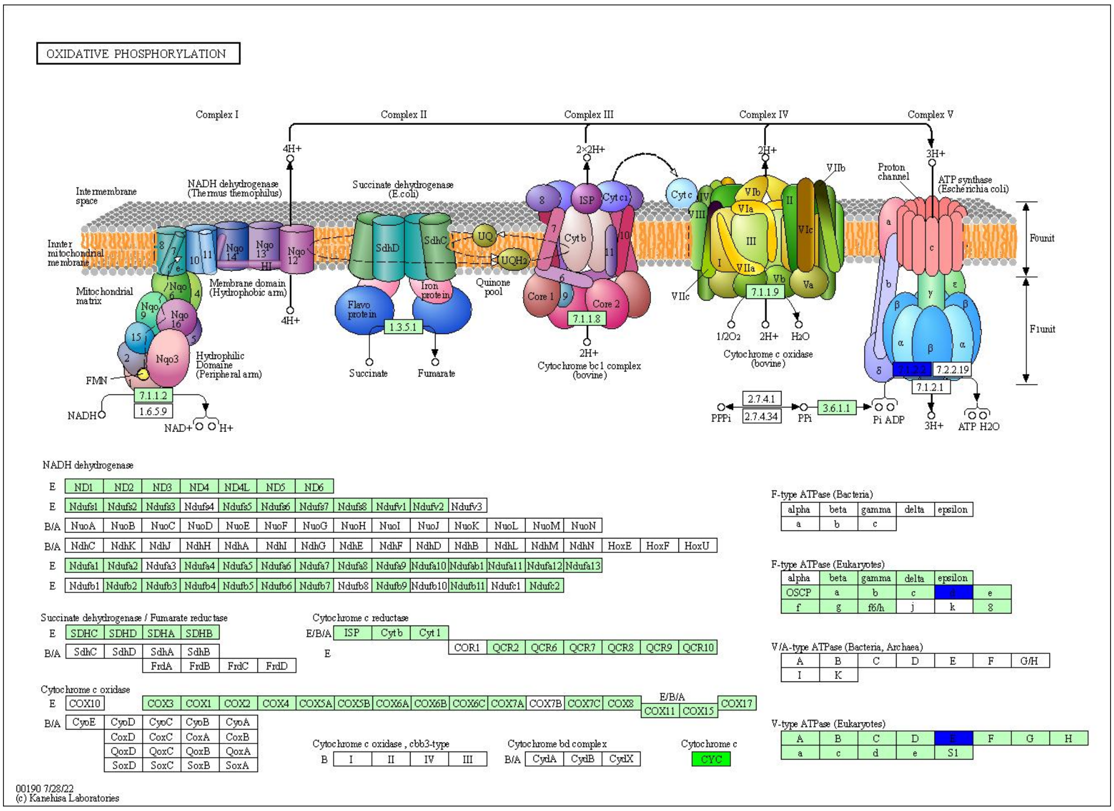

Figure S1. Differentially expressed proteins involved in the oxidative phosphorylation pathway of diamondback moth, shown in green as down-regulated and blue as unchanged

Characterization of target compounds 1-32

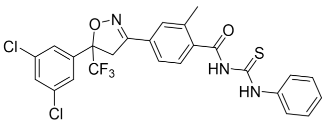

4-(5-(3,5-dichlorophenyl)-5-(trifluoromethyl)-4,5-dihydroisoxazol-3-yl)-2-methyl-*N*-(phenylcarbamothioyl)benzamide(1). Yellow solid; yield: 47.6%; m.p. 93.9-95.8 °C; ^{1}H NMR (400 MHz, DMSO-d_6) δ 10.41 (s, 1H, -NH-), 7.82 (t, J = 1.8 Hz, 1H, Ar-H), 7.74 (d, J = 7.8 Hz, 2H, Ar-H), 7.66 (dd, J = 11.1, 5.0 Hz, 4H, Ar-H), 7.59 (d, J = 7.8 Hz, 1H, Ar-H), 7.35 (t, J = 7.9 Hz, 2H, Ar-H), 7.11 (t, J = 7.4 Hz, 1H, Ar-H), 4.37 (q, J = 18.4 Hz, 2H, isoxazoline-H), 2.43 (s, 3H, -CH_3). ^{13}C NMR (101 MHz, DMSO-d_6) δ 167.44, 157.93, 139.87, 139.50, 139.14, 136.60, 135.13, 130.06, 129.73, 129.19, 128.70, 128.34, 126.24, 124.85, 124.22, 120.21, 87.25, 86.96, 43.35, 19.67. HRMS (ESI) m/z $[M-H]^-$ calcd for $C_{25}H_{17}Cl_2F_3N_3O_2S$: 550.0365, found: 550.0377.

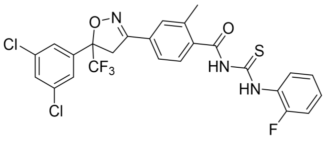

4-(5-(3,5-dichlorophenyl)-5-(trifluoromethyl)-4,5-dihydroisoxazol-3-yl)-*N*-((2-fluorophenyl)carbamothioyl)-2-methylbenzamide(2). Yellow solid; yield: 46.3%; m.p. 150.7-152.2 °C; ^{1}H NMR (400 MHz, DMSO-d_6) δ 10.22 (s, 1H, -NH-), 7.83 (t, J = 1.9 Hz, 1H, Ar-H), 7.76 – 7.59 (m, 6H, Ar-H), 7.27 (m, 3H, Ar-H), 4.38 (q, J = 18.4 Hz, 2H, isoxazoline-H), 2.46 (s, 3H, -CH_3). ^{13}C NMR (101 MHz, DMSO-d_6) δ 167.64, 157.94, 156.96, 154.51, 139.18, 139.15, 136.80, 135.12, 134.43, 130.06, 129.75, 128.83, 128.49, 127.71, 127.34, 126.90, 126.24, 125.91, 125.79, 124.85, 124.81, 116.39, 116.20, 87.26, 86.96, 43.34, 19.75. HRMS (ESI) m/z $[M-H]^-$ calcd for $C_{25}H_{16}Cl_2F_4N_3O_2S$: 568.0271, found: 568.0287.

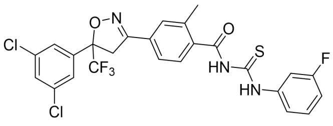

4-(5-(3,5-dichlorophenyl)-5-(trifluoromethyl)-4,5-dihydroisoxazol-3-yl)-*N*-((3-fluorophenyl)carbamothioyl)-2-methylbenzamide(3). White solid; yield: 31.7%; m.p.

174.6-175.5 °C; ^{1}H NMR (400 MHz, DMSO-d_6) δ 10.62 (s, 1H, -NH-), 7.84 – 7.80 (m, 1H, Ar-H), 7.77 – 7.58 (m, 6H, Ar-H), 7.49 (d, J = 8.2 Hz, 1H, Ar-H), 7.45 – 7.35 (m, 1H, Ar-H), 6.95 (td, J = 8.5, 2.5 Hz, 1H, Ar-H), 4.38 (q, J = 18.4 Hz, 2H, isoxazoline-H), 2.43 (s, 3H, -CH$_3$). ^{13}C NMR (101 MHz, DMSO-d_6) δ 167.68, 163.77, 161.37, 157.90, 141.25, 141.15, 139.40, 139.14, 136.73, 135.13, 130.90, 130.81, 130.05, 129.80, 128.94, 128.40, 126.23, 124.87, 115.95, 110.79, 110.58, 107.06, 106.80, 87.29, 86.99, 43.34, 19.66. HRMS (ESI) m/z [M-H]$^-$ calcd for $C_{25}H_{16}Cl_2F_4N_3O_2S$: 568.0271, found: 568.0286.

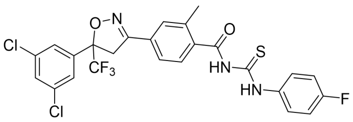

4-(5-(3,5-dichlorophenyl)-5-(trifluoromethyl)-4,5-dihydroisoxazol-3-yl)-*N*-((4-fluorophenyl)carbamothioyl)-2-methylbenzamide(4). Yellow solid; yield: 48.8%; m.p. 205.4-207.5 °C; ^{1}H NMR (400 MHz, DMSO-d_6) δ 12.34 (s, 1H, -NH-), 11.89 (s, 1H, -NH-), 7.83 (t, J = 1.8 Hz, 1H, Ar-H), 7.70 (dd, J = 8.7, 4.7 Hz, 3H, Ar-H), 7.67 – 7.53 (m, 4H, Ar-H), 7.32 – 7.23 (m, 2H, Ar-H), 4.38 (q, J = 18.4 Hz, 2H, isoxazoline-H), 2.47 (s, 3H, -CH$_3$). ^{13}C NMR (101 MHz, DMSO-d_6) δ 179.84, 170.05, 157.90, 139.10, 137.33, 136.70, 135.13, 130.08, 129.77, 129.24, 127.38, 126.21, 124.65, 115.98, 115.76, 87.38, 43.27, 19.82. HRMS (ESI) m/z [M-H]$^-$ calcd for $C_{25}H_{16}Cl_2F_4N_3O_2S$: 568.0271, found: 568.0281.

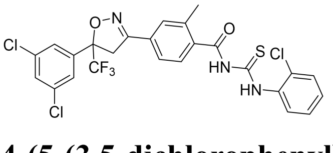

4-(5-(3,5-dichlorophenyl)-5-(trifluoromethyl)-4,5-dihydroisoxazol-3-yl)-*N*-((2-chlorophenyl)carbamothioyl)-2-methylbenzamide(5). Yellow solid; yield: 33.3%; m.p. 155.9-156.7 °C; ^{1}H NMR (500 MHz, DMSO-d_6) δ 10.12 (s, 1H, -NH-), 7.78 (t, J = 1.8 Hz, 1H, Ar-H), 7.64 (d, J = 4.8 Hz, 2H, Ar-H), 7.60 (d, J = 1.6 Hz, 4H, Ar-H), 7.51 (dd, J = 7.9, 0.9 Hz, 1H, Ar-H), 7.35 (t, J = 7.2 Hz, 1H, Ar-H), 7.26 (t, J = 7.6 Hz, 1H, Ar-H), 4.33 (dd, J = 43.4, 18.4 Hz, 2H, isoxazoline-H), 2.44 (s, 3H, -CH$_3$). ^{13}C NMR (126 MHz, DMSO-d_6) δ 167.75, 157.99, 139.20, 136.94, 135.14, 130.13, 129.82, 128.90, 128.50, 128.11, 126.29, 125.52, 124.88, 123.26, 87.29, 87.05, 43.40, 19.91. HRMS (ESI) m/z [M-H]$^-$ calcd for $C_{25}H_{16}Cl_3F_3N_3O_2S$: 583.9975, found:

583.9992.

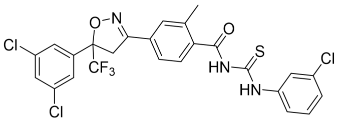

4-(5-(3,5-dichlorophenyl)-5-(trifluoromethyl)-4,5-dihydroisoxazol-3-yl)-*N*-((3-chlorophenyl)carbamothioyl)-2-methylbenzamide(6). Yellow solid; yield: 33.3%; m.p. 96.5-97.7 °C; ^{1}H NMR (400 MHz, DMSO-d_6) δ 10.60 (s, 1H, -NH-), 7.95 (s, 1H, Ar-H), 7.82 (t, J = 1.8 Hz, 1H, Ar-H), 7.69 (d, J = 8.7 Hz, 2H, Ar-H), 7.65 – 7.57 (m, 4H, Ar-H), 7.38 (t, J = 8.1 Hz, 1H, Ar-H), 7.17 (dd, J = 8.0, 1.3 Hz, 1H, Ar-H), 4.38 (q, J = 18.4 Hz, 2H, isoxazoline-H), 2.43 (s, 3H, -CH_3). ^{13}C NMR (101 MHz, DMSO-d_6) δ 167.70, 157.90, 140.92, 139.23, 136.76, 135.13, 133.53, 130.93, 130.06, 129.80, 128.95, 128.41, 126.24, 124.88, 123.94, 119.63, 118.59, 87.28, 86.99, 43.33, 19.67. HRMS (ESI) m/z [M-H]$^-$ calcd for $C_{25}H_{16}Cl_3F_3N_3O_2S$: 583.9975, found: 583.9991.

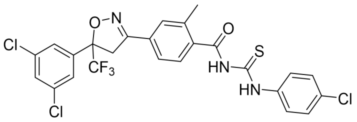

4-(5-(3,5-dichlorophenyl)-5-(trifluoromethyl)-4,5-dihydroisoxazol-3-yl)-*N*-((4-chlorophenyl)carbamothioyl)-2-methylbenzamide(7). White solid; yield: 50.0%; m.p. 156.7-157.3 °C; ^{1}H NMR (500 MHz, DMSO-d_6) δ 10.52 (s, 1H, -NH-), 7.78 (t, J = 1.8 Hz, 1H, Ar-H), 7.73 (d, J = 8.9 Hz, 2H, Ar-H), 7.65 – 7.59 (m, 4H, Ar-H), 7.55 (d, J = 7.9 Hz, 1H, Ar-H), 7.37 (d, J = 8.8 Hz, 2H, Ar-H), 4.33 (dd, J = 40.8, 18.4 Hz, 2H, isoxazoline-H), 2.38 (s, 3H, -CH_3). ^{13}C NMR (126 MHz, DMSO-d_6) δ 167.56, 157.98, 139.54, 139.18, 138.51, 136.77, 135.18, 130.12, 129.84, 129.19, 128.92, 128.46, 127.85, 126.31, 124.92, 121.76, 87.29, 87.05, 43.37, 19.74. HRMS (ESI) m/z [M-H]$^-$ calcd for $C_{25}H_{16}Cl_3F_3N_3O_2S$: 583.9975, found: 584.9977.

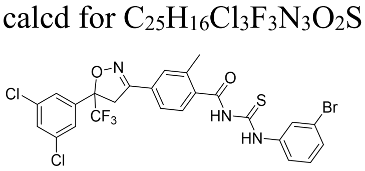

4-(5-(3,5-dichlorophenyl)-5-(trifluoromethyl)-4,5-dihydroisoxazol-3-yl)-*N*-((3-bromophenyl)carbamothioyl)-2-methylbenzamide(8). Yellow solid; yield: 57.7%; m.p. 176.6-178.5 °C; ^{1}H NMR (500 MHz, DMSO-d_6) δ 12.40 (s, 1H, -NH-), 11.91 (s, 1H, -NH-), 8.04 (s, 1H, Ar-H), 7.80 – 7.76 (m, 1H, Ar-H), 7.66 – 7.55 (m, 6H, Ar-H),

7.45 – 7.42 (m, 1H, Ar-H), 7.35 (t, J = 8.0 Hz, 1H, Ar-H), 4.34 (dd, J = 43.0, 18.4 Hz, 2H, isoxazoline-H), 2.42 (s, 3H, $-CH_3$). ^{13}C NMR (126 MHz, DMSO-d_6) δ 179.67, 170.06, 157.94, 140.03, 139.15, 137.46, 136.64, 135.19, 131.12, 130.13, 129.89, 129.85, 129.61, 129.34, 127.76, 127.45, 126.28, 124.70, 124.14, 121.52, 87.17, 43.32, 19.89. HRMS (ESI) m/z $[M-H]^-$ calcd for $C_{25}H_{16}BrCl_2F_3N_3O_2S$: 627.9470, found: 627.9481.

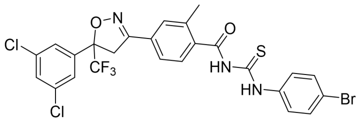

4-(5-(3,5-dichlorophenyl)-5-(trifluoromethyl)-4,5-dihydroisoxazol-3-yl)-*N*-((4-bromophenyl)carbamothioyl)-2-methylbenzamide(9). White solid; yield: 31.1%; m.p. 157.0-157.6 °C; ^1H NMR (500 MHz, DMSO-d_6) δ 10.50 (s, 1H, -NH-), 7.77 (t, J = 1.9 Hz, 1H, Ar-H), 7.68 (d, J = 8.9 Hz, 2H, Ar-H), 7.63 (dd, J = 10.2, 2.1 Hz, 2H, Ar-H), 7.60 (d, J = 1.7 Hz, 2H, Ar-H), 7.55 (d, J = 7.9 Hz, 1H, Ar-H), 7.51 – 7.48 (m, 2H, Ar-H), 4.33 (dd, J = 41.6, 18.4 Hz, 2H, isoxazoline-H), 2.38 (s, 3H, $-CH_3$). ^{13}C NMR (126 MHz, DMSO-d_6) δ 167.58, 157.97, 139.54, 139.18, 138.92, 136.77, 135.18, 132.09, 130.12, 129.84, 128.93, 128.45, 126.30, 125.52, 124.92, 123.25, 122.15, 115.93, 87.30, 87.06, 43.39, 19.74. HRMS (ESI) m/z $[M-H]^-$ calcd for $C_{25}H_{16}BrCl_2F_3N_3O_2S$: 627.9470, found: 627.9443.

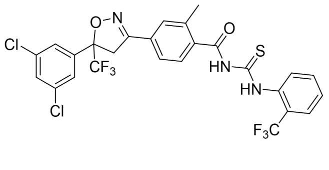

4-(5-(3,5-dichlorophenyl)-5-(trifluoromethyl)-4,5-dihydroisoxazol-3-yl)-2-methyl-*N*-((2-(trifluoromethyl)phenyl)carbamothioyl)benzamide(10). White solid; yield: 38.9%; m.p. 133.8-135.0 °C; ^1H NMR (400 MHz, DMSO-d_6) δ 10.23 (s, 1H, -NH-), 7.82 (dd, J = 5.8, 4.0 Hz, 2H, Ar-H), 7.76 (t, J = 7.6 Hz, 1H, Ar-H), 7.70 (d, J = 7.0 Hz, 2H, Ar-H), 7.65 (d, J = 1.6 Hz, 2H, Ar-H), 7.61 – 7.53 (m, 3H, Ar-H), 4.38 (q, J = 18.4 Hz, 2H, isoxazoline-H), 2.46 (s, 3H, $-CH_3$). ^{13}C NMR (101 MHz, DMSO-d_6) δ 168.62, 157.92, 139.10, 136.89, 135.64, 135.13, 133.69, 131.71, 130.05, 129.81, 128.85, 128.23, 126.23, 124.82, 87.26, 86.97, 43.36, 19.57. HRMS (ESI) m/z $[M-H]^-$ calcd for $C_{26}H_{16}Cl_2F_6N_3O_2S$: 618.0239, found: 618.0255.

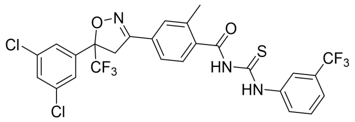

4-(5-(3,5-dichlorophenyl)-5-(trifluoromethyl)-4,5-dihydroisoxazol-3-yl)-2-methyl-*N*-((3-(trifluoromethyl)phenyl)carbamothioyl)benzamide(11). Yellow solid; yield: 45.5%; m.p. 70.5-72.8 °C; ^{1}H NMR (400 MHz, DMSO-d_6) δ 10.80 (s, 1H, -NH-), 8.30 (s, 1H, Ar-H), 8.00 (d, J = 8.3 Hz, 1H, Ar-H), 7.87 (t, J = 1.9 Hz, 1H, Ar-H), 7.78 – 7.72 (m, 2H, Ar-H), 7.71 – 7.63 (m, 4H, Ar-H), 7.52 (d, J = 7.8 Hz, 1H, Ar-H), 4.43 (q, J = 18.4 Hz, 2H, isoxazoline-H), 2.49 (s, 3H, -CH_3). ^{13}C NMR (101 MHz, DMSO-d_6) δ 167.86, 157.90, 140.25, 139.15, 136.86, 135.13, 130.48, 130.06, 129.81, 129.03, 128.48, 126.23, 125.93, 124.88, 123.75, 123.23, 120.54, 116.25, 87.29, 87.00, 43.33, 19.70. HRMS (ESI) m/z [M-H]$^-$ calcd for $C_{26}H_{16}Cl_2F_6N_3O_2S$: 618.0239, found: 618.0255.

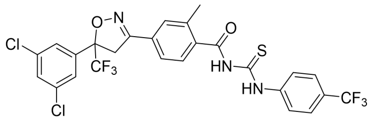

4-(5-(3,5-dichlorophenyl)-5-(trifluoromethyl)-4,5-dihydroisoxazol-3-yl)-2-methyl-*N*-((4-(trifluoromethyl)phenyl)carbamothioyl)benzamide(12). White solid; yield: 55.1%; m.p. 80.0-81.6 °C; ^{1}H NMR (400 MHz, DMSO-d_6) δ 10.78 (s, 1H, -NH-), 7.96 (d, J = 8.5 Hz, 2H, Ar-H), 7.82 (t, J = 1.8 Hz, 1H, Ar-H), 7.74 – 7.70 (m, 3H, Ar-H), 7.69 – 7.54 (m, 4H, Ar-H), 4.38 (q, J = 18.4 Hz, 2H, isoxazoline-H), 2.44 (s, 3H, -CH_3). ^{13}C NMR (101 MHz, DMSO-d_6) δ 167.92, 157.90, 143.06, 139.17, 136.79, 135.13, 130.06, 129.82, 129.03, 128.47, 126.53, 126.23, 124.88, 124.38, 124.07, 123.48, 120.09, 87.29, 87.00, 43.33, 19.67. HRMS (ESI) m/z [M-H]$^-$ calcd for $C_{26}H_{16}Cl_2F_6N_3O_2S$: 618.0239, found: 618.0255.

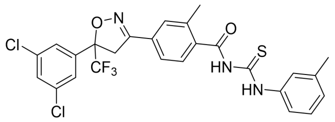

4-(5-(3,5-dichlorophenyl)-5-(trifluoromethyl)-4,5-dihydroisoxazol-3-yl)-2-methyl-*N*-(m-tolylcarbamothioyl)benzamide(13). Yellow solid; yield: 35.0%; m.p. 197.3-199.2 °C; ^{1}H NMR (500 MHz, DMSO-d_6) δ 12.41 (s, 1H, -NH-), 11.82 (s, 1H, -NH-),, 7.79 (t, J = 1.9 Hz, 1H, Ar-H), 7.66 – 7.57 (m, 5H, Ar-H), 7.53 – 7.44 (m, 2H, Ar-H), 7.27 (t, J = 7.9 Hz, 1H, Ar-H), 7.05 (d, J = 7.6 Hz, 1H, Ar-H), 4.43 – 4.25 (m,

2H, isoxazoline-H), 2.42 (s, 3H, $-CH_3$), 2.29 (s, 3H, $-CH_3$). ^{13}C NMR (126 MHz, DMSO-d_6) δ 179.16, 170.25, 157.96, 139.15, 138.69, 138.31, 137.37, 136.77, 135.19, 130.14, 129.82, 129.31, 129.06, 127.56, 126.28, 125.14, 124.70, 121.81, 87.39, 87.16, 43.32, 21.46, 19.88. HRMS (ESI) m/z $[M-H]^-$ calcd for $C_{26}H_{19}Cl_2F_3N_3O_2S$: 564.0522, found: 564.0543.

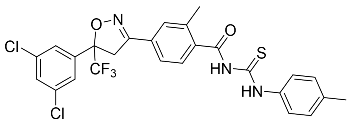

4-(5-(3,5-dichlorophenyl)-5-(trifluoromethyl)-4,5-dihydroisoxazol-3-yl)-2-methyl-*N*-(p-tolylcarbamothioyl)benzamide(14). Yellow solid; yield: 31.7%; m.p. 169.1-170.7 °C; ^1H NMR (400 MHz, DMSO-d_6) δ 12.46 (s, 1H, -NH-), 11.87 (s, 1H, -NH-), 7.83 (t, J = 1.8 Hz, 1H, Ar-H), 7.69 (dd, J = 10.9, 4.9 Hz, 5H, Ar-H), 7.61 (d, J = 8.3 Hz, 2H, Ar-H), 7.26 (d, J = 8.3 Hz, 2H, Ar-H), 4.43 (t, J = 19.0 Hz, 2H, isoxazoline-H), 2.50 (s, 3H, $-CH_3$), 2.35 (s, 3H, $-CH_3$). ^{13}C NMR (101 MHz, DMSO-d_6) δ 179.14, 170.17, 157.85, 139.11, 137.32, 136.72, 136.16, 135.81, 135.13, 130.04, 129.82 – 129.48, 129.23, 126.20, 125.72, 124.61, 122.89, 87.38, 87.08, 43.29, 21.07, 19.82. HRMS (ESI) m/z $[M-H]^-$ calcd for $C_{26}H_{19}Cl_2F_3N_3O_2S$: 564.0522, found: 564.0536.

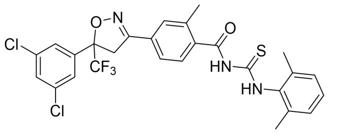

4-(5-(3,5-dichlorophenyl)-5-(trifluoromethyl)-4,5-dihydroisoxazol-3-yl)-*N*-((2,6-dimethylphenyl)carbamothioyl)-2-methylbenzamide(15). Yellow solid; yield: 45.2%; m.p. 71.0-72.1 °C; ^1H NMR (500 MHz, DMSO-d_6) δ 11.84 (s, 1H, -NH-), 11.68 (s, 1H, -NH-), 7.76 (s, 1H, Ar-H), 7.65 (s, 1H, Ar-H), 7.61 (d, J = 7.8 Hz, 4H, Ar-H), 7.08 (d, J = 8.2 Hz, 3H, Ar-H), 4.38 (d, J = 18.3 Hz, 1H, isoxazoline-H), 4.29 (d, J = 18.4 Hz, 1H, isoxazoline-H), 2.42 (s, 3H, $-CH_3$), 2.18 (s, 6H, $-2CH_3$). ^{13}C NMR (126 MHz, DMSO-d_6) δ 180.47, 169.99, 157.94, 139.18, 137.19, 136.96, 136.76, 135.62, 135.18, 130.11, 129.77, 129.19, 128.42, 126.27, 124.76, 87.39, 87.16, 43.34, 19.86, 18.37. HRMS (ESI) m/z $[M-H]^-$ calcd for $C_{27}H_{21}Cl_2F_3N_3O_2S$: 578.0678, found: 578.0693.

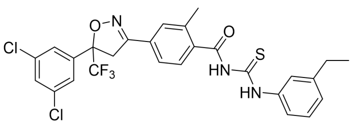

4-(5-(3,5-dichlorophenyl)-5-(trifluoromethyl)-4,5-dihydroisoxazol-3-yl)-*N*-((3-ethylphenyl)carbamothioyl)-2-methylbenzamide(16). Yellow solid; yield: 35.7%; m.p. 62.8-63.5 °C; ^{1}H NMR (400 MHz, DMSO-*d*$_6$) δ 10.39 (s, 1H, -NH-), 7.88 (t, J = 1.9 Hz, 1H, Ar-H), 7.76 – 7.66 (m, 5H, Ar-H), 7.61 (dd, J = 13.4, 8.0 Hz, 2H, Ar-H), 7.31 (t, J = 7.8 Hz, 1H, Ar-H), 7.01 (d, J = 7.6 Hz, 1H, Ar-H), 4.43 (q, J = 18.4 Hz, 2H, isoxazoline-H), 2.65 (q, J = 7.6 Hz, 2H, -CH$_2$-), 2.48 (s, 3H, -CH$_3$), 1.24 (t, J = 7.6 Hz, 3H, -CH$_3$). ^{13}C NMR (101 MHz, DMSO-*d*$_6$) δ 167.39, 157.94, 144.75, 139.94, 139.51, 139.15, 136.59, 135.13, 130.05, 129.72, 129.08, 128.67, 128.33, 126.24, 124.83, 123.71, 119.57, 117.67, 87.25, 86.95, 43.35, 28.77, 19.69, 16.02. HRMS (ESI) m/z [M-H]$^-$ calcd for $C_{27}H_{21}Cl_2F_3N_3O_2S$: 578.0678, found: 578.0699.

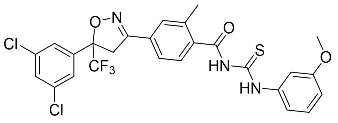

4-(5-(3,5-dichlorophenyl)-5-(trifluoromethyl)-4,5-dihydroisoxazol-3-yl)-*N*-((3-methoxyphenyl)carbamothioyl)-2-methylbenzamide(17). Yellow solid; yield: 35.7%; m.p. 53.6-54.8°C; ^{1}H NMR (400 MHz, DMSO-*d*$_6$) δ 10.39 (s, 1H, -NH-), 7.83 (t, J = 1.8 Hz, 1H, Ar-H), 7.66 (dd, J = 10.1, 5.2 Hz, 4H, Ar-H), 7.58 (d, J = 7.8 Hz, 1H, Ar-H), 7.44 (s, 1H, Ar-H), 7.27 (dt, J = 16.0, 8.1 Hz, 2H, Ar-H), 6.69 (dd, J = 8.0, 1.4 Hz, 1H, Ar-H), 4.37 (q, J = 18.4 Hz, 2H, isoxazoline-H), 3.75 (s, 3H, -CH$_3$), 2.43 (s, 3H, -CH$_3$). ^{13}C NMR (101 MHz, DMSO-*d*$_6$) δ 167.47, 159.96, 157.93, 140.67, 139.83, 139.14, 136.60, 135.13, 130.03, 129.74, 128.73, 128.33, 126.24, 124.84, 112.45, 109.66, 105.96, 87.26, 86.96, 55.48, 43.34, 19.67. HRMS (ESI) m/z [M-H]$^-$ calcd for $C_{26}H_{19}Cl_2F_3N_3O_3S$: 580.0471, found: 580.0488.

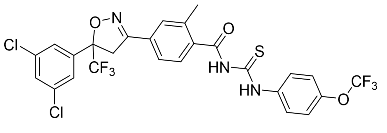

4-(5-(3,5-dichlorophenyl)-5-(trifluoromethyl)-4,5-dihydroisoxazol-3-yl)-2-methyl-*N*-((4-(trifluoromethoxy)phenyl)carbamothioyl)benzamide(18). Yellow solid; yield: 56.5%; m.p. 173.4-175.7 °C; ^{1}H NMR (400 MHz, DMSO-*d*$_6$) δ 12.43 (s, 1H, -NH-), 11.92 (s, 1H, -NH-), 7.89 – 7.79 (m, 3H, Ar-H), 7.72 – 7.59 (m, 5H, Ar-H),

7.40 (dd, J = 25.8, 8.7 Hz, 2H, Ar-H), 4.48 – 4.29 (m, 2H, isoxazoline-H), 2.47 (s, 3H, -CH_3). ^{13}C NMR (101 MHz, DMSO-d_6) δ 179.77, 170.02, 157.88, 146.39, 139.11, 137.57, 137.38, 136.64, 135.13, 130.07, 129.78, 129.27, 128.40, 126.89, 126.21, 124.65, 122.12, 121.79, 121.56, 87.39, 87.09, 43.28, 19.82. HRMS (ESI) m/z $[M-H]^-$ calcd for $C_{26}H_{16}Cl_2F_6N_3O_3S$: 634.0188, found: 634.0203.

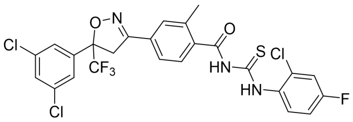

4-(5-(3,5-dichlorophenyl)-5-(trifluoromethyl)-4,5-dihydroisoxazol-3-yl)-*N*-((2-chloro-4-fluorophenyl)carbamothioyl)-2-methylbenzamide(19). Yellow solid; yield: 65.1%; m.p. 172.2-174.7 °C; ^1H NMR (400 MHz, DMSO-d_6) δ 12.32 (s, 1H, -NH-), 12.10 (s, 1H, -NH-), 7.95 (dd, J = 9.0, 5.8 Hz, 1H, Ar-H), 7.83 (t, J = 1.8 Hz, 1H, Ar-H), 7.69 (s, 1H, Ar-H), 7.66 (s, 2H, Ar-H), 7.65 – 7.60 (m, 3H, Ar-H), 7.32 (td, J = 8.6, 2.9 Hz, 1H, Ar-H), 4.38 (q, J = 18.4 Hz, 2H, isoxazoline-H), 2.47 (s, 3H, -CH_3). ^{13}C NMR (101 MHz, DMSO-d_6) δ 180.96, 170.26, 157.89, 139.10, 137.35, 136.48, 135.13, 130.49, 130.18 – 129.68, 129.29, 126.22, 124.68, 117.35, 117.09, 115.12, 114.90, 87.09, 43.28, 19.82. HRMS (ESI) m/z $[M-H]^-$ calcd for $C_{25}H_{15}Cl_3F_4N_3O_2S$: 601.9881, found: 601.9894.

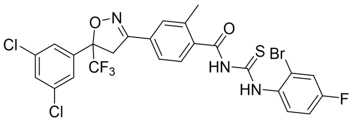

4-(5-(3,5-dichlorophenyl)-5-(trifluoromethyl)-4,5-dihydroisoxazol-3-yl)-*N*-((2-bromo-4-fluorophenyl)carbamothioyl)-2-methylbenzamide(20). Yellow solid; yield: 52.4%; m.p. 128.7-130.0 °C; ^1H NMR (400 MHz, DMSO-d_6) δ 12.25 (s, 1H, -NH-), 12.10 (s, 1H, -NH-), 7.90 – 7.81 (m, 2H, Ar-H), 7.74 (dd, J = 8.3, 2.9 Hz, 1H, Ar-H), 7.69 (s, 1H, Ar-H), 7.68 – 7.54 (m, 4H, Ar-H), 7.36 (td, J = 8.6, 2.9 Hz, 1H, Ar-H), 4.38 (q, J = 18.4 Hz, 2H, isoxazoline-H), 2.47 (s, 3H, -CH_3). ^{13}C NMR (101 MHz, DMSO-d_6) δ 181.08, 170.25, 157.89, 139.10, 137.32, 136.50, 135.13, 130.99, 130.08, 129.83, 129.24, 126.21, 124.68, 120.23, 119.98, 115.62, 115.39, 87.40, 43.26, 19.82. HRMS (ESI) m/z $[M-H]^-$ calcd for $C_{25}H_{15}BrCl_2F_4N_3O_2S$: 645.9376, found: 645.9388.

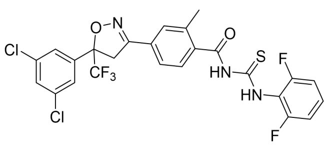

4-(5-(3,5-dichlorophenyl)-5-(trifluoromethyl)-4,5-dihydroisoxazol-3-yl)-*N*-((2,6-di fluorophenyl)carbamothioyl)-2-methylbenzamide(21). White solid; yield: 61.9%; m.p. 77.9-78.6 °C; ^{1}H NMR (500 MHz, DMSO-d_6) δ 10.17 (s, 1H, -NH-), 7.74 (s, 1H, Ar-H), 7.67 (s, 2H, Ar-H), 7.63 – 7.56 (m, 3H, Ar-H), 7.37 (s, 1H, Ar-H), 7.18 (d, J = 6.7 Hz, 2H, Ar-H), 4.34 (dd, J = 41.6, 18.3 Hz, 2H, isoxazoline-H), 2.44 (s, 3H, -CH$_3$). ^{13}C NMR (126 MHz, DMSO-d_6) δ 167.81, 159.57, 157.91, 157.59, 139.23, 138.60, 136.99, 135.18, 130.00, 129.04, 128.47, 126.27, 125.51, 124.94, 123.25, 114.69, 112.45, 87.58, 87.34, 87.11, 43.42, 19.76. HRMS (ESI) m/z [M+K]$^+$ calcd for $C_{25}H_{16}Cl_2F_5N_3O_2SK$: 625.9892, found: 625.9923.

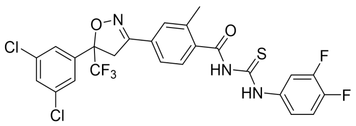

4-(5-(3,5-dichlorophenyl)-5-(trifluoromethyl)-4,5-dihydroisoxazol-3-yl)-*N*-((3,4-di fluorophenyl)carbamothioyl)-2-methylbenzamide(22). Yellow solid; yield: 64.3%; m.p. 143.0-143.8 °C; ^{1}H NMR (400 MHz, DMSO-d_6) δ 10.64 (s, 1H, -NH-), 7.91 (dd, J = 9.8, 7.3, 1.8 Hz, 1H, Ar-H), 7.82 (t, J = 1.8 Hz, 1H, Ar-H), 7.69 (d, J = 8.4 Hz, 2H, Ar-H), 7.62 (dd, J = 16.0, 4.7 Hz, 3H, Ar-H), 7.49 – 7.38 (m, 2H, Ar-H), 4.37 (q, J = 18.4 Hz, 2H, isoxazoline-H), 2.43 (s, 3H, -CH$_3$). ^{13}C NMR (101 MHz, DMSO-d_6) δ 167.55, 157.89, 148.22, 139.16, 136.80, 136.53, 135.13, 130.06, 129.83, 128.99, 128.40, 126.23, 124.87, 117.95, 116.52, 109.27, 109.05, 87.29, 86.99, 43.33, 19.67. HRMS (ESI) m/z [M-H]$^-$ calcd for $C_{25}H_{15}Cl_2F_5N_3O_2S$: 586.0177, found: 586.0197.

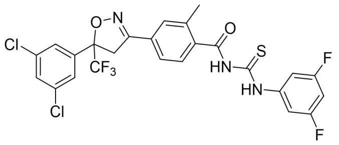

4-(5-(3,5-dichlorophenyl)-5-(trifluoromethyl)-4,5-dihydroisoxazol-3-yl)-*N*-((3,5-di fluorophenyl)carbamothioyl)-2-methylbenzamide(23). White solid; yield: 45.2%; m.p. 77.1-78.8 °C; ^{1}H NMR (500 MHz, DMSO-d_6) δ 10.75 (s, 1H, -NH-), 7.77 (t, J = 1.9 Hz, 1H, Ar-H), 7.65 (d, J = 9.9 Hz, 2H, Ar-H), 7.60 (d, J = 1.7 Hz, 2H, Ar-H),

7.57 (d, *J* = 7.9 Hz, 1H, Ar-H), 7.43 (dt, *J* = 6.7, 3.3 Hz, 2H, Ar-H), 6.93 (tt, *J* = 9.3, 2.3 Hz, 1H, Ar-H), 4.33 (dd, *J* = 42.0, 18.4 Hz, 2H, isoxazoline-H), 2.38 (s, 3H, -CH_3). ^{13}C NMR (126 MHz, DMSO-d_6) δ 167.92, 163.91, 161.98, 157.93, 141.98, 139.17, 138.97, 136.95, 135.18, 130.11, 129.93, 129.21, 128.50, 126.29, 125.51, 124.95, 123.24, 103.04, 99.52, 99.40 – 99.22, 99.21, 87.33, 87.10, 43.37, 19.72. HRMS (ESI) m/z [M-H]$^-$ calcd for $C_{25}H_{15}Cl_2F_5N_3O_2S$: 586.0177, found: 586.0190.

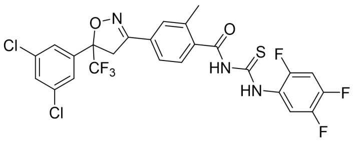

4-(5-(3,5-dichlorophenyl)-5-(trifluoromethyl)-4,5-dihydroisoxazol-3-yl)-2-methyl-*N*-((2,4,5-trifluorophenyl)carbamothioyl)benzamide(24). Yellow solid; yield: 62.8%; m.p. 56.1-57.8 °C; ^{1}H NMR (400 MHz, DMSO-d_6) δ 10.44 (s, 1H, -NH-), 7.99 – 7.92 (m, 1H, Ar-H), 7.86 (t, *J* = 1.9 Hz, 1H, Ar-H), 7.73 (d, *J* = 7.4 Hz, 3H, Ar-H), 7.69 (d, *J* = 1.6 Hz, 2H, Ar-H), 7.66 (d, *J* = 8.3 Hz, 1H, Ar-H), 4.44 (t, *J* = 17.5 Hz, 2H, isoxazoline-H), 2.49 (s, 3H, -CH_3). ^{13}C NMR (101 MHz, DMSO-d_6) δ 167.76, 157.90, 139.14, 138.60, 136.95, 135.12, 130.04, 129.78, 129.06, 128.59, 126.22, 125.74, 124.79, 122.79, 122.64 – 122.57, 114.74, 114.52, 106.81, 106.57, 106.33, 87.29, 86.99, 43.34, 19.74. HRMS (ESI) m/z [M-H]$^-$ calcd for $C_{25}H_{14}Cl_2F_6N_3O_2S$: 604.0082, found: 604.0098.

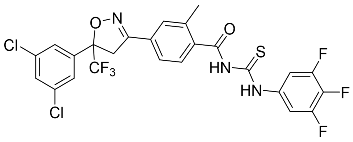

4-(5-(3,5-dichlorophenyl)-5-(trifluoromethyl)-4,5-dihydroisoxazol-3-yl)-2-methyl-*N*-((3,4,5-trifluorophenyl)carbamothioyl)benzamide(25). White solid; yield: 40.2%; m.p. 182.4-183.1 °C; ^{1}H NMR (500 MHz, DMSO-d_6) δ 10.73 (s, 1H, -NH-), 7.77 (t, *J* = 1.8 Hz, 1H, Ar-H), 7.65 (d, *J* = 9.0 Hz, 2H, Ar-H), 7.62 – 7.59 (m, 4H, Ar-H), 7.56 (d, *J* = 7.8 Hz, 1H, Ar-H), 4.35 (t, *J* = 21.3 Hz, 2H, isoxazoline-H), 2.38 (s, 3H, -CH_3). ^{13}C NMR (126 MHz, DMSO-d_6) δ 167.84, 157.91, 151.51, 149.59, 139.16, 138.78, 137.00, 135.79, 135.18, 134.50, 130.04, 129.26, 128.49, 126.28, 124.95, 104.58, 104.40, 87.33, 87.09, 43.35, 19.73. HRMS (ESI) m/z [M-H]$^-$ calcd for

$C_{25}H_{14}Cl_2F_6N_3O_2S$: 604.0082, found: 604.0096.

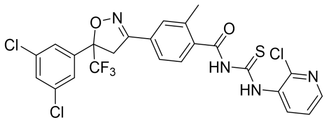

4-(5-(3,5-dichlorophenyl)-5-(trifluoromethyl)-4,5-dihydroisoxazol-3-yl)-*N*-((2-chloropyridin-3-yl)carbamothioyl)-2-methylbenzamide(26). White solid; yield: 66.7%; m.p. 75.1-76.2 °C; ^{1}H NMR (400 MHz, DMSO-d_6) δ 12.53 (s, 1H, -NH-), 12.20 (s, 1H, -NH-), 8.50 – 8.45 (m, 1H, Ar-H), 8.35 (dd, J = 4.6, 1.5 Hz, 1H, Ar-H), 7.82 (s, 1H, Ar-H), 7.69 (d, J = 9.5 Hz, 3H, Ar-H), 7.64 (s, 2H, Ar-H), 7.53 (dd, J = 7.9, 4.7 Hz, 1H, Ar-H), 4.43 (d, J = 18.4 Hz, 1H, isoxazoline-H), 4.34 (d, J = 18.4 Hz, 1H, isoxazoline-H), 2.47 (s, 3H, $-CH_3$). ^{13}C NMR (101 MHz, DMSO-d_6) δ 180.74, 170.29, 157.87, 147.80, 146.06, 139.10, 137.33, 136.35, 135.13, 133.20, 130.25 – 129.65, 129.34, 126.21, 124.77, 123.65, 122.27, 87.39, 87.10, 43.27, 19.83. HRMS (ESI) m/z $[M-H]^-$ calcd for $C_{24}H_{15}Cl_3F_3N_4O_2S$: 584.9928, found: 584.9946.

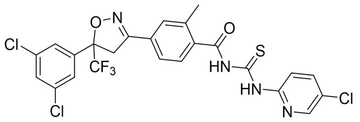

4-(5-(3,5-dichlorophenyl)-5-(trifluoromethyl)-4,5-dihydroisoxazol-3-yl)-*N*-((4-chloropyridin-2-yl)carbamothioyl)-2-methylbenzamide(27). Yellow solid; yield: 38.1%; m.p. 82.5-83.7 °C; ^{1}H NMR (500 MHz, DMSO-d_6) δ 8.44 (s, 1H, Ar-H), 8.00 (dd, J = 8.9, 2.6 Hz, 1H, Ar-H), 7.76 (t, J = 1.8 Hz, 1H, Ar-H), 7.65 – 7.58 (m, 6H, Ar-H), 4.33 (dd, J = 44.3, 18.4 Hz, 2H, isoxazoline-H), 2.41 (s, 3H, $-CH_3$). ^{13}C NMR (126 MHz, DMSO-d_6) δ 178.30, 157.90, 150.23, 147.45, 139.15, 138.34, 137.56, 136.48, 135.18, 130.30 – 129.68, 129.36, 126.26, 124.71, 117.04, 87.41, 87.17, 43.33, 19.87. HRMS (ESI) m/z $[M-H]^-$ calcd for $C_{24}H_{15}Cl_3F_3N_4O_2S$: 584.9928, found: 584.9948.

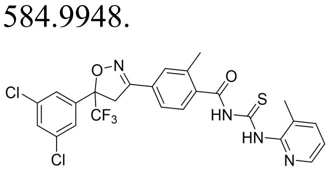

4-(5-(3,5-dichlorophenyl)-5-(trifluoromethyl)-4,5-dihydroisoxazol-3-yl)-2-methyl-*N*-((2-methylpyridin-6-yl)carbamothioyl)benzamide(28). Yellow solid; yield: 39.0%; m.p. 76.1-77.9 °C; ^{1}H NMR (500 MHz, DMSO-d_6) δ 12.08 (s, 1H, -NH-), 11.95 (s, 1H, -NH-), 8.29 (s, 1H, Ar-H), 7.77 (s, 1H, Ar-H), 7.71 (d, J = 7.2 Hz, 1H,

Ar-H), 7.64 (s, 1H, Ar-H), 7.61 (d, *J* = 9.7 Hz, 4H, Ar-H), 7.28 (d, *J* = 4.4 Hz, 1H, Ar-H), 4.38 (d, *J* = 18.3 Hz, 1H, isoxazoline-H), 4.29 (d, *J* = 18.3 Hz, 1H, isoxazoline-H), 2.42 (s, 3H, $-CH_3$), 2.27 (s, 3H, $-CH_3$). ^{13}C NMR (126 MHz, DMSO-d_6) δ 180.45, 157.94, 150.68, 146.78, 140.06, 139.16, 137.36, 136.77, 135.18, 130.11, 129.83, 129.27, 126.27, 124.74, 87.40, 87.16, 43.33, 19.87, 17.69. HRMS (ESI) m/z $[M-H]^-$ calcd for $C_{25}H_{18}Cl_2F_3N_4O_2S$: 565.0474, found: 565.0487.

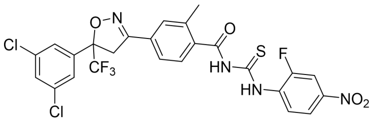

4-(5-(3,5-dichlorophenyl)-5-(trifluoromethyl)-4,5-dihydroisoxazol-3-yl)-*N*-((2-fluoro-4-nitrophenyl)carbamothioyl)-2-methylbenzamide(29). Yellow solid; yield: 34.1%; m.p. 62.5-63.8 °C; ^1H NMR (500 MHz, DMSO-d_6) δ 10.73 (s, 1H, -NH-), 8.23 (t, *J* = 7.0 Hz, 1H, Ar-H), 8.18 (d, *J* = 10.3 Hz, 1H, Ar-H), 8.12 (d, *J* = 8.8 Hz, 1H, Ar-H), 7.76 (s, 1H, Ar-H), 7.64 (d, *J* = 10.6 Hz, 2H, Ar-H), 7.60 (d, *J* = 7.2 Hz, 3H, Ar-H), 4.33 (dd, *J* = 42.4, 18.4 Hz, 2H, isoxazoline-H), 2.40 (s, 3H, $-CH_3$). ^{13}C NMR (126 MHz, DMSO-d_6) δ 168.10, 157.95, 154.28, 152.28, 144.12, 139.17, 138.43, 137.12, 135.18, 133.20, 130.10, 129.85, 129.32, 128.86, 126.28, 125.51, 124.86, 124.62, 123.24, 120.86, 112.42, 112.22, 87.34, 87.10, 43.37, 19.81. HRMS (ESI) m/z $[M-H]^-$ calcd for $C_{25}H_{15}Cl_2F_4N_4O_4S$: 613.0122, found: 613.0136.

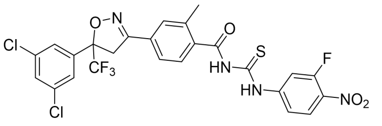

4-(5-(3,5-dichlorophenyl)-5-(trifluoromethyl)-4,5-dihydroisoxazol-3-yl)-*N*-((3-fluoro-4-nitrophenyl)carbamothioyl)-2-methylbenzamide(30). Yellow solid; yield: 40.9%; m.p. 88.1-89.3 °C; ^1H NMR (500 MHz, DMSO-d_6) δ 12.73 (s, 1H, -NH-), 12.10 (s, 1H, -NH-), 8.27 (d, *J* = 13.3 Hz, 1H, Ar-H), 8.17 (t, *J* = 8.2 Hz, 1H, Ar-H), 7.74 (d, *J* = 15.8 Hz, 2H, Ar-H), 7.63 (d, *J* = 8.6 Hz, 2H, Ar-H), 7.59 (s, 3H, Ar-H), 4.33 (dd, *J* = 45.8, 18.4 Hz, 2H, isoxazoline-H), 2.42 (s, 3H, $-CH_3$). ^{13}C NMR (126 MHz, DMSO-d_6) δ 179.58, 169.95, 157.89, 156.38, 154.31, 145.24, 139.14, 137.64, 136.34, 135.18, 133.81, 130.35 – 129.70, 129.44, 127.36, 126.25, 125.48, 124.70, 123.21, 120.04, 112.51, 112.31, 87.42, 87.18, 43.31, 19.91. HRMS (ESI) m/z $[M-H]^-$ calcd for $C_{25}H_{15}Cl_2F_4N_4O_4S$: 613.0122, found: 613.0135.

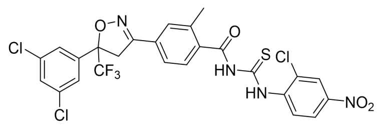

4-(5-(3,5-dichlorophenyl)-5-(trifluoromethyl)-4,5-dihydroisoxazol-3-yl)-*N*-((2-chloro-4-nitrophenyl)carbamothioyl)-2-methylbenzamide(31). Yellow solid; yield: 35.6%; m.p. 63.4-65.3 °C; ^{1}H NMR (500 MHz, DMSO-d_6) δ 10.45 (s, 1H, -NH-), 8.37 (d, J = 2.6 Hz, 1H, Ar-H), 8.23 (dd, J = 9.0, 2.6 Hz, 1H, Ar-H), 8.08 (d, J = 9.0 Hz, 1H, Ar-H), 7.78 (t, J = 1.8 Hz, 1H, Ar-H), 7.68 – 7.64 (m, 3H, Ar-H), 7.60 (d, J = 1.7 Hz, 2H, Ar-H), 4.38 (d, J = 18.4 Hz, 1H, isoxazoline-H), 4.29 (d, J = 18.4 Hz, 1H, isoxazoline-H), 2.45 (s, 3H, -CH$_3$). ^{13}C NMR (126 MHz, DMSO-d_6) δ 167.89, 157.97, 145.05, 141.43, 139.16, 138.42, 137.19, 135.19, 130.14, 130.04, 129.34, 128.77, 128.14, 127.07, 126.29, 125.53, 124.93, 123.51, 87.33, 87.10, 43.36, 19.94. HRMS (ESI) m/z [M-H]$^-$ calcd for $C_{25}H_{15}Cl_3F_3N_4O_4S$: 628.9826, found: 628.9838.

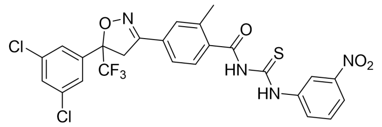

4-(5-(3,5-dichlorophenyl)-5-(trifluoromethyl)-4,5-dihydroisoxazol-3-yl)-2-methyl-*N*-((3-nitrophenyl)carbamothioyl)benzamide(32). Yellow solid; yield: 44.2%; m.p. 76.6-77.3 °C; ^{1}H NMR (500 MHz, DMSO-d_6) δ 10.87 (s, 1H, -NH-), 8.76 (s, 1H, Ar-H), 8.03 (dd, J = 8.2, 1.1 Hz, 1H, Ar-H), 7.93 (dd, J = 8.3, 2.3, 0.7 Hz, 1H, Ar-H), 7.76 (t, J = 1.9 Hz, 1H, Ar-H), 7.66 (d, J = 8.3 Hz, 2H, Ar-H), 7.62 (s, 1H, Ar-H), 7.60 (dd, J = 5.0, 3.2 Hz, 3H, Ar-H), 4.34 (dd, J = 43.2, 18.4 Hz, 2H, isoxazoline-H), 2.41 (s, 3H, -CH$_3$). ^{13}C NMR (126 MHz, DMSO-d_6) δ 168.01, 157.93, 148.44, 140.67, 139.18, 138.93, 137.04, 135.18, 130.70, 130.02, 129.22, 128.58, 126.25, 125.51, 124.95, 123.24, 118.80, 114.32, 87.33, 87.09, 43.38, 19.79. HRMS (ESI) m/z [M-H]$^-$ calcd for $C_{25}H_{16}Cl_2F_3N_4O_4S$: 595.0216, found: 595.0229.

^{1}H NMR, ^{13}C NMR and HRMS spectrum of the compounds 1-32

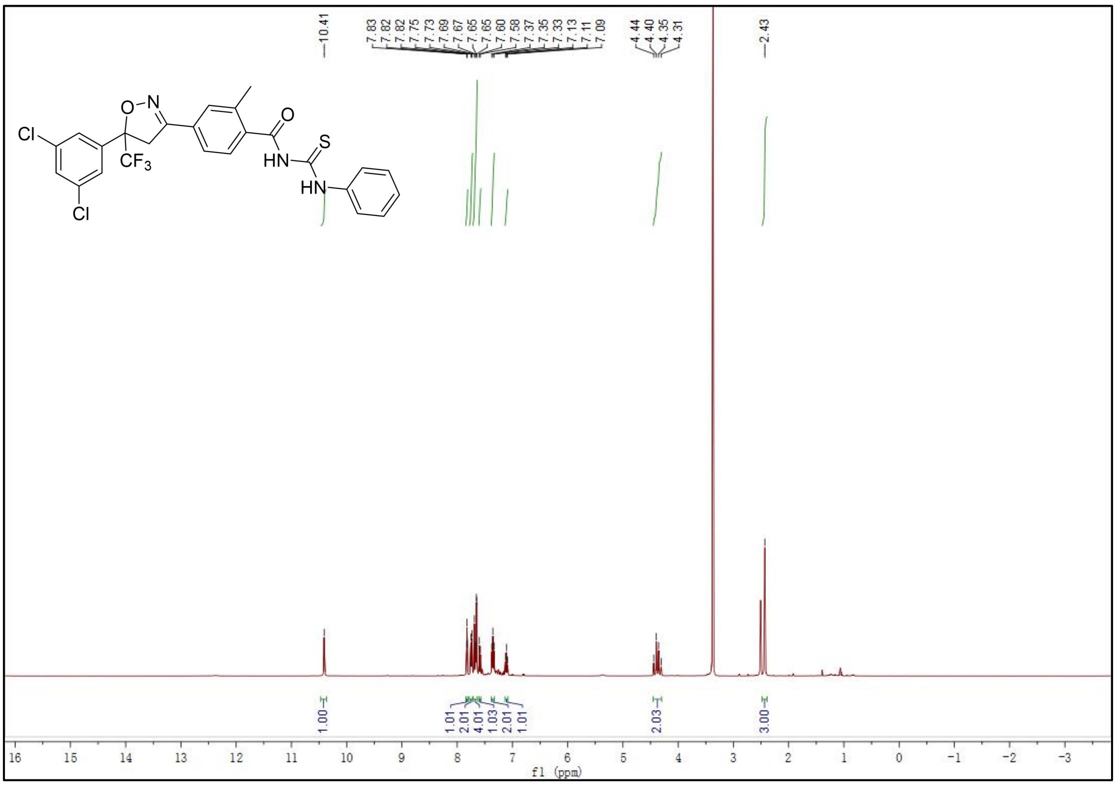

Figure 1. ^{1}H NMR spectrum of compound **1**.

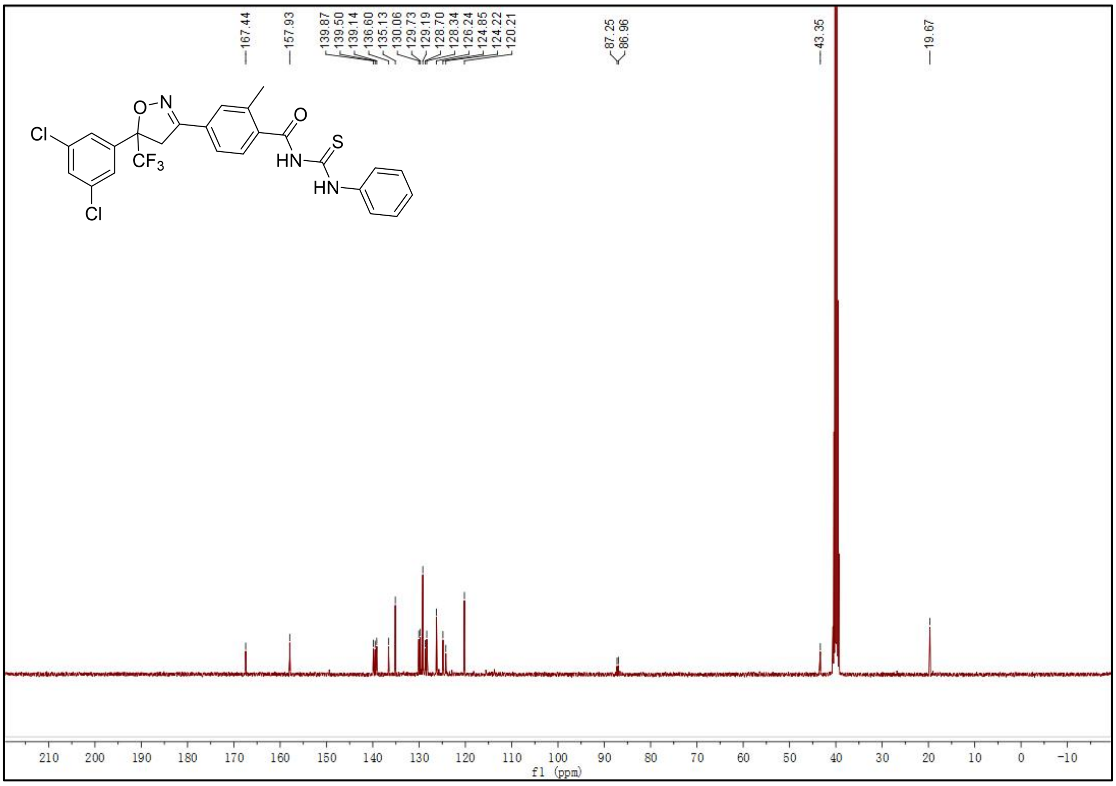

Figure 2. ^{13}C NMR spectrum of compound **1**.

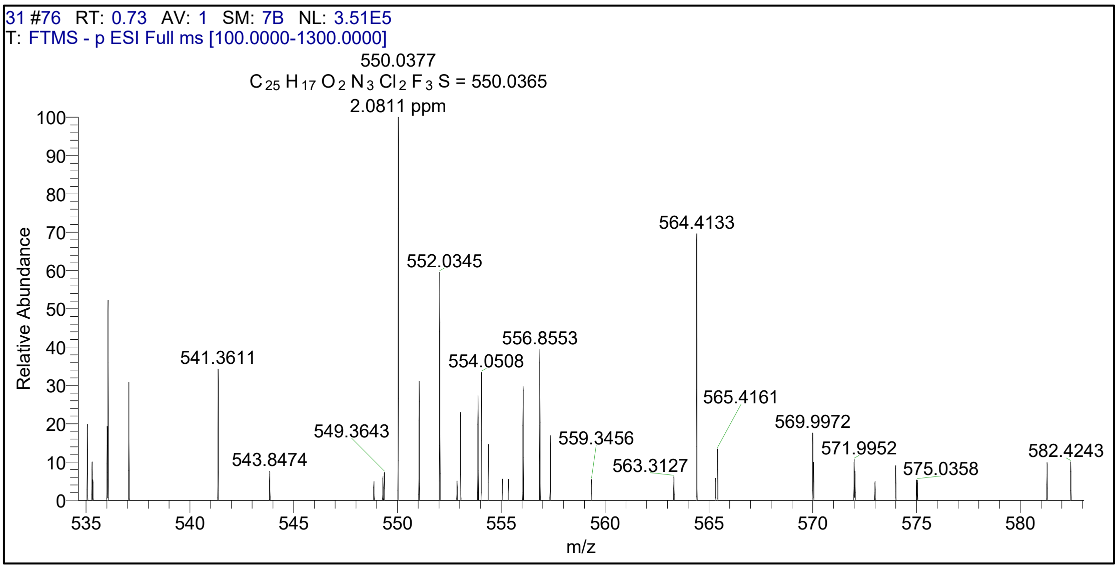

Figure 3. HRMS(ESI) of compound **1.**

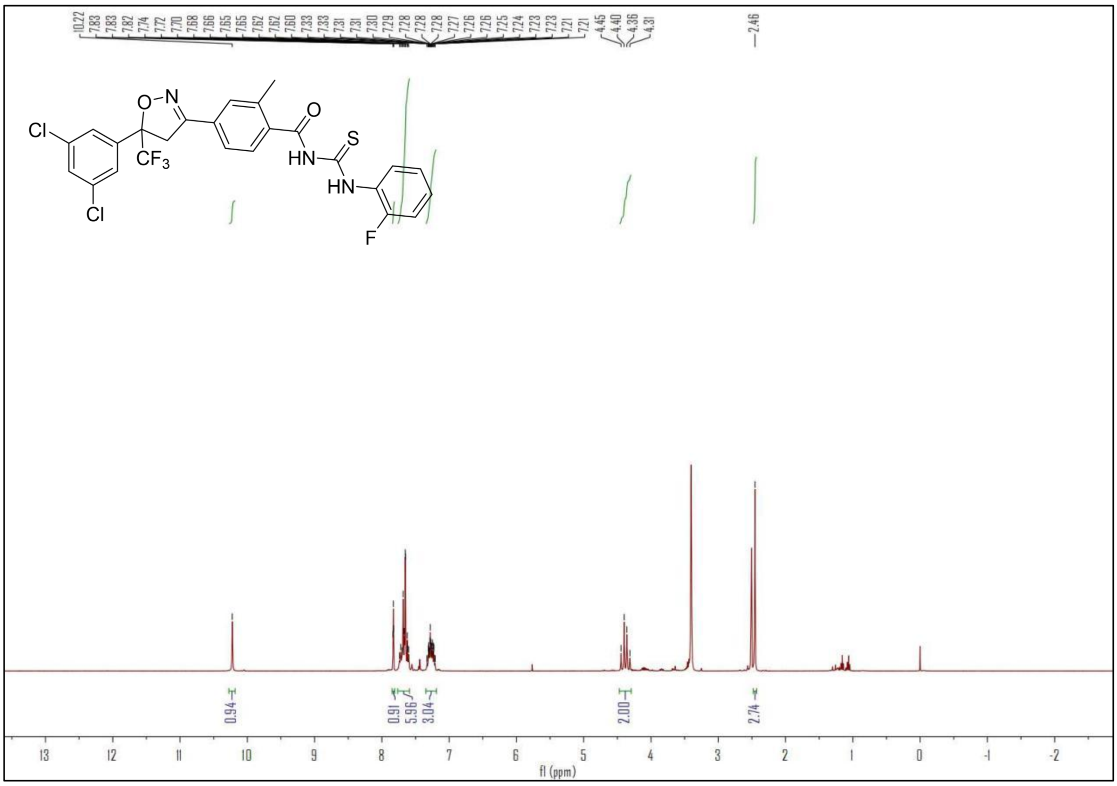

Figure 4. ^{1}H NMR spectrum of compound **2**.

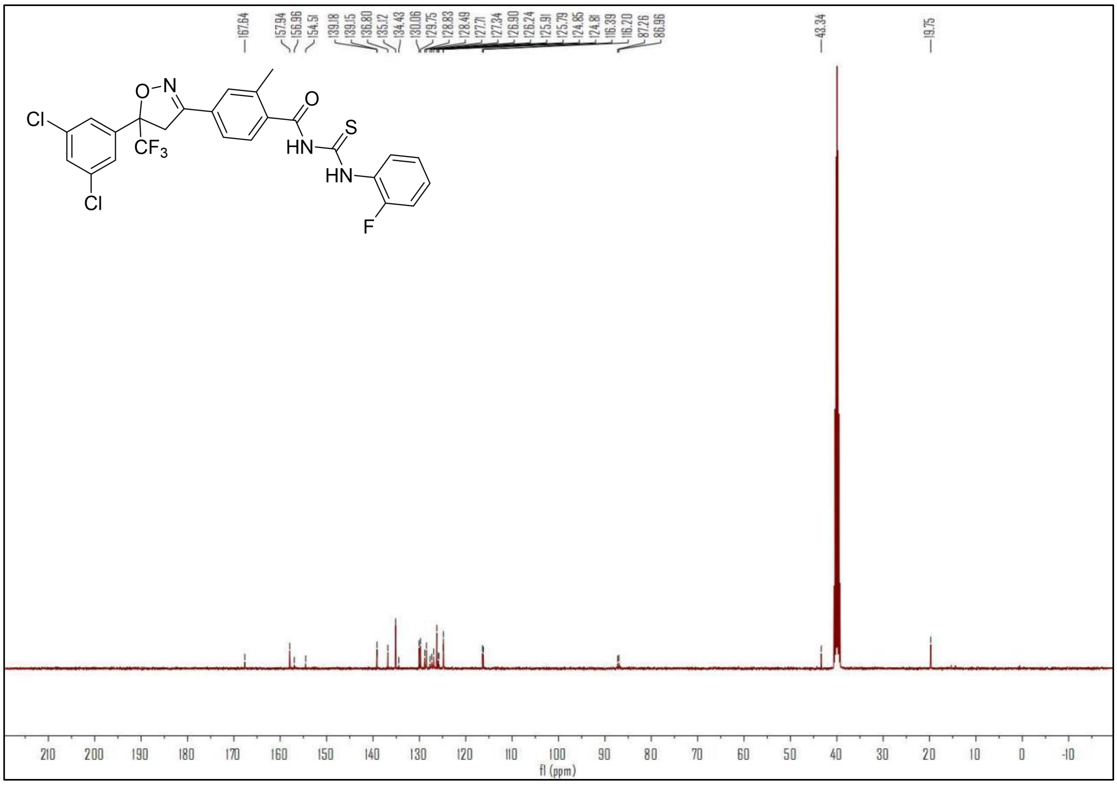

Figure 5. ^{13}C NMR spectrum of compound **2**.

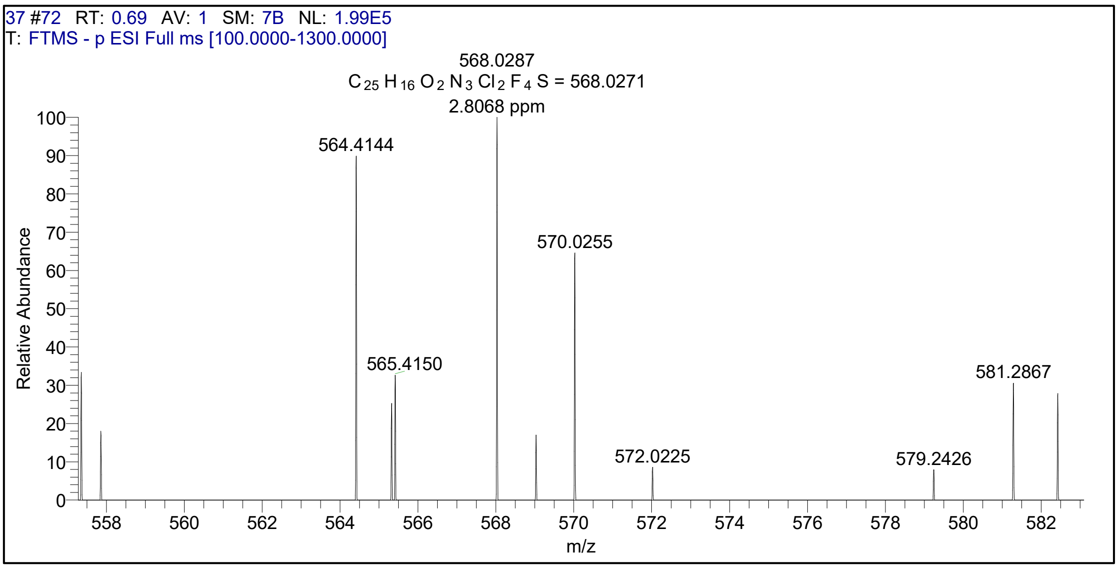

Figure 6. HRMS(ESI) of compound **2.**

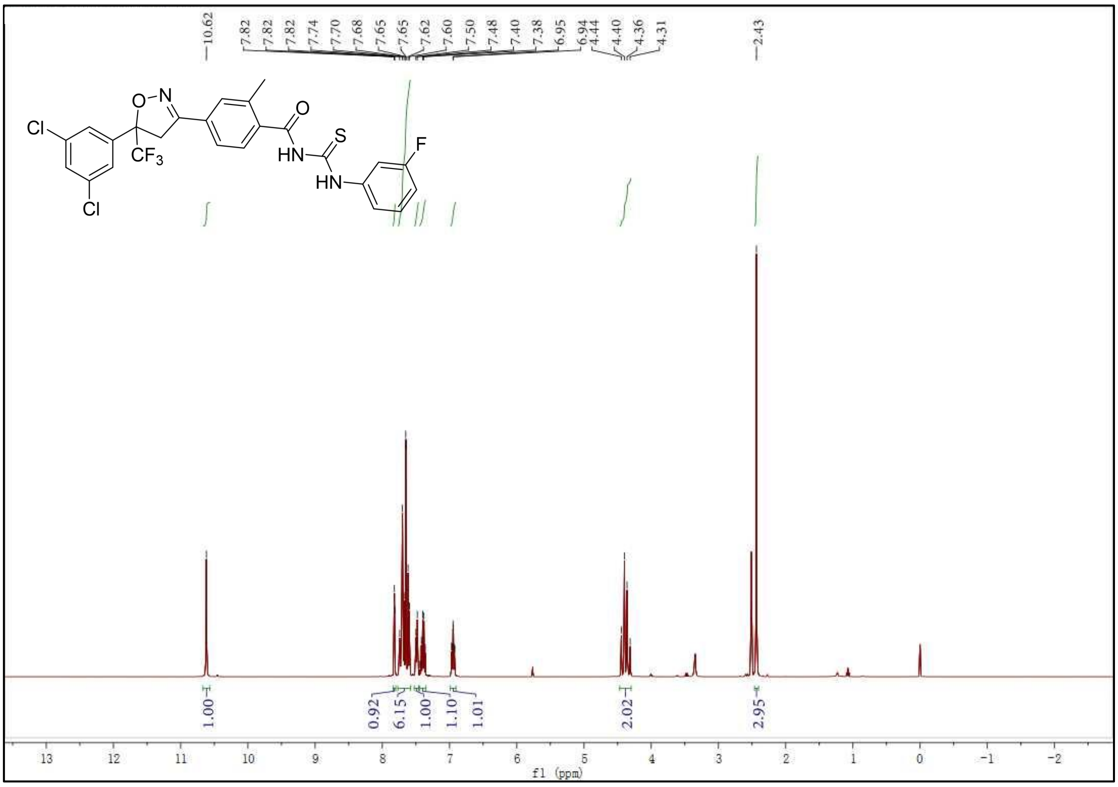

Figure 7. ^{1}H NMR spectrum of compound **3**.

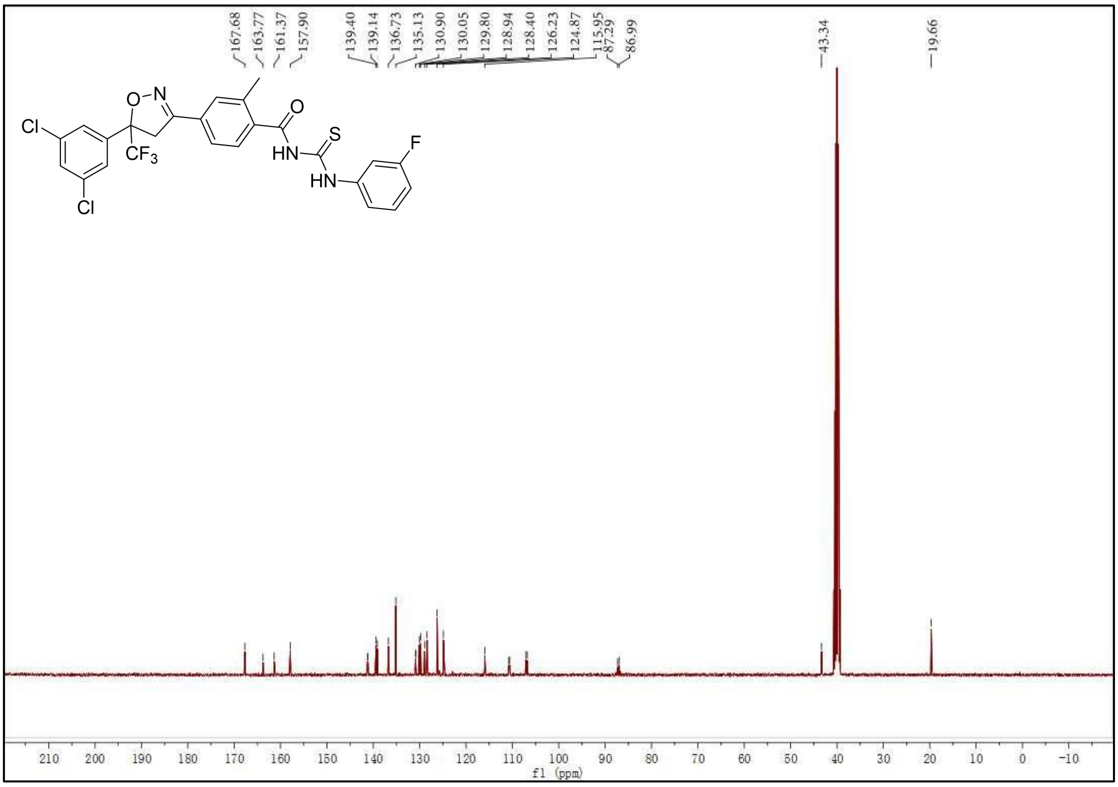

Figure 8. ^{13}C NMR spectrum of compound **3**.

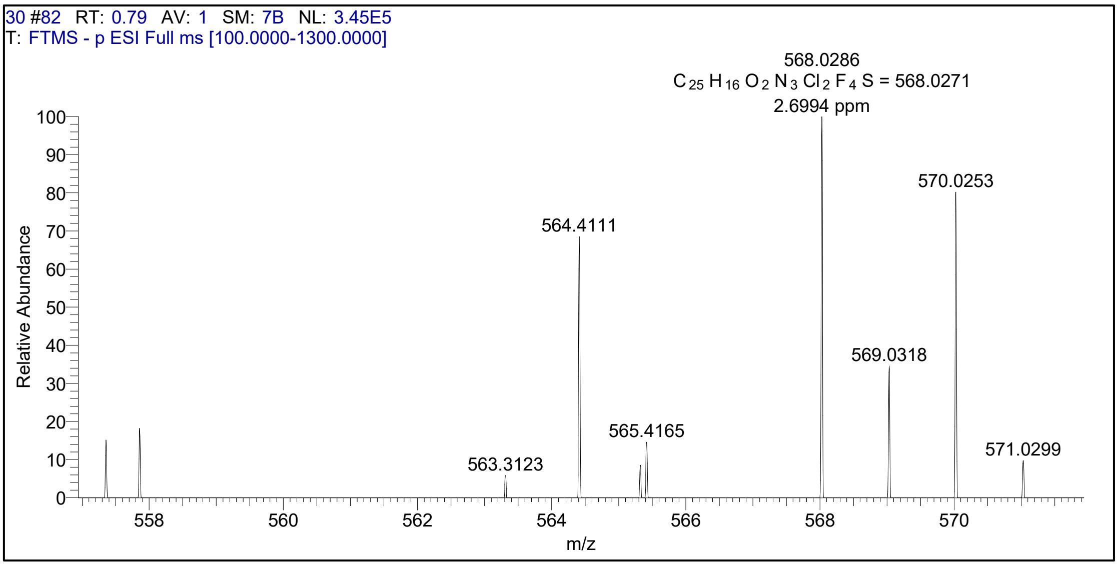

Figure 9. HRMS(ESI) of compound **3.**

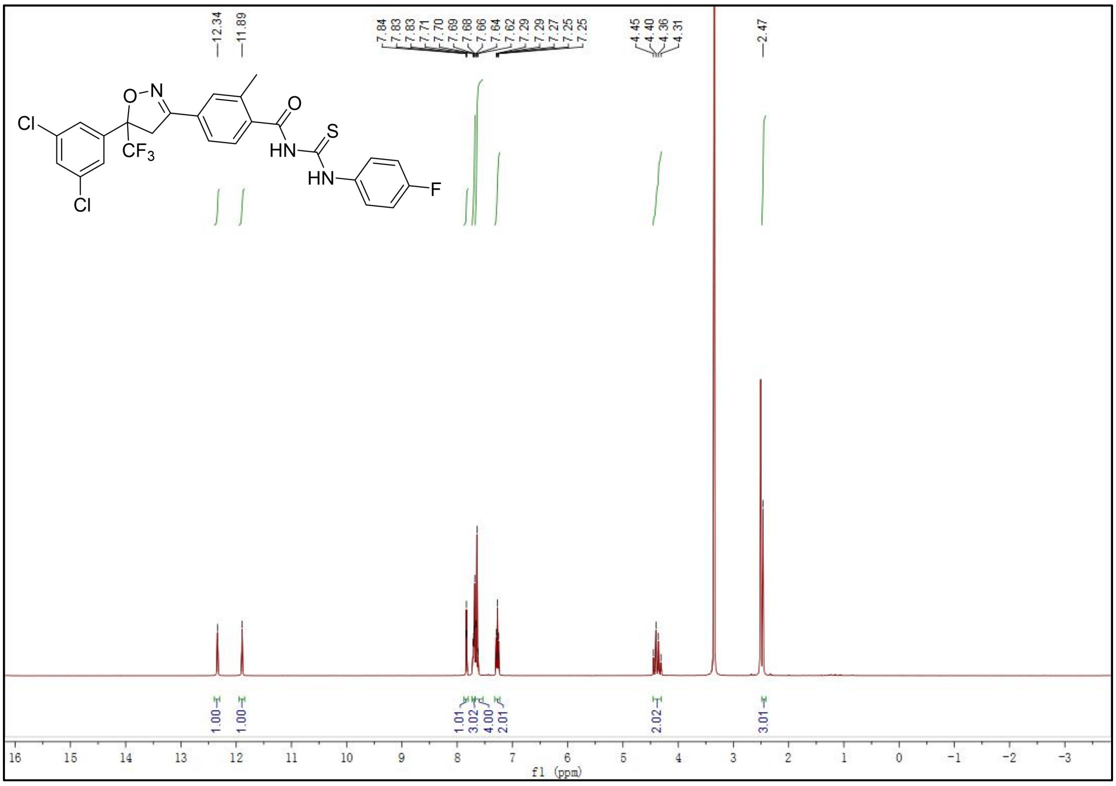

Figure 10. ^{1}H NMR spectrum of compound **4**.

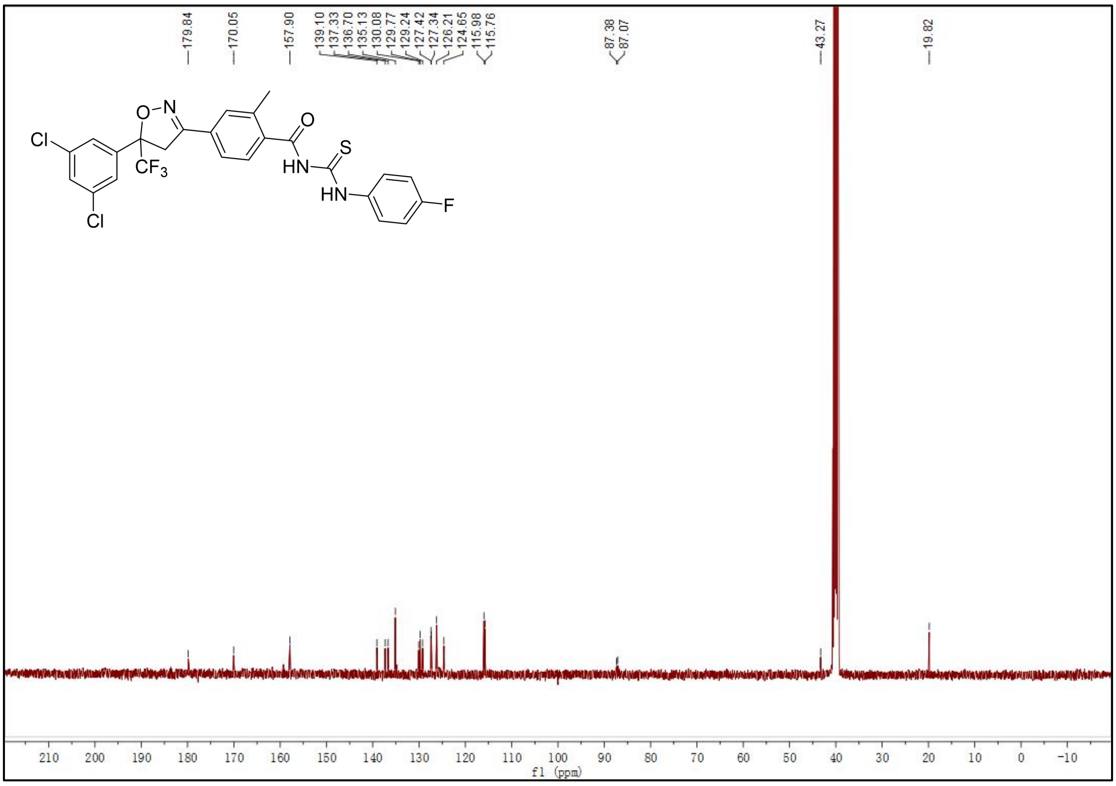

Figure 11. ^{13}C NMR spectrum of compound **4**.

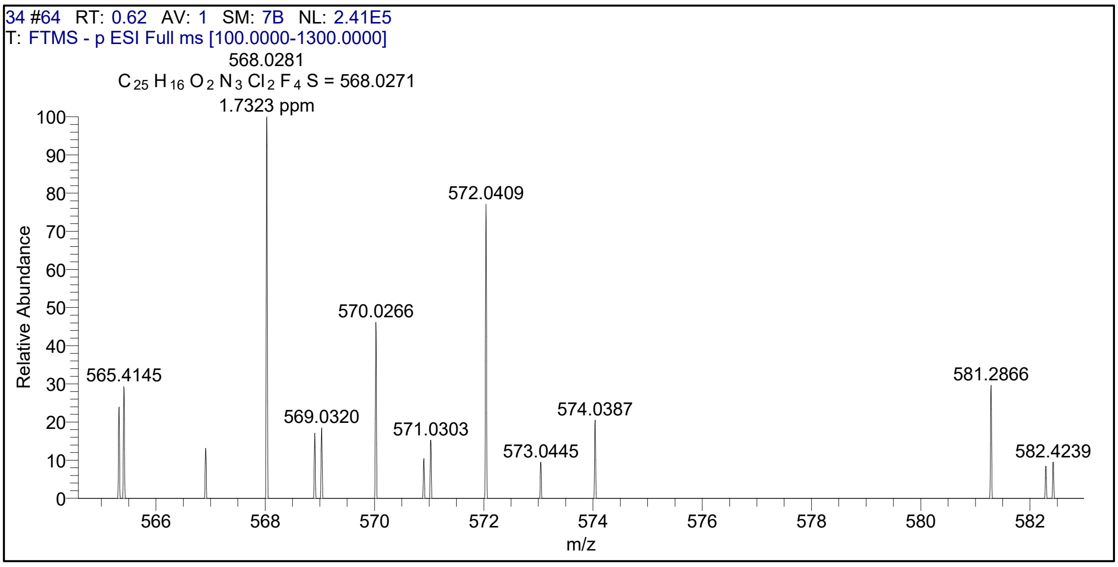

Figure 12. HRMS(ESI) of compound **4.**

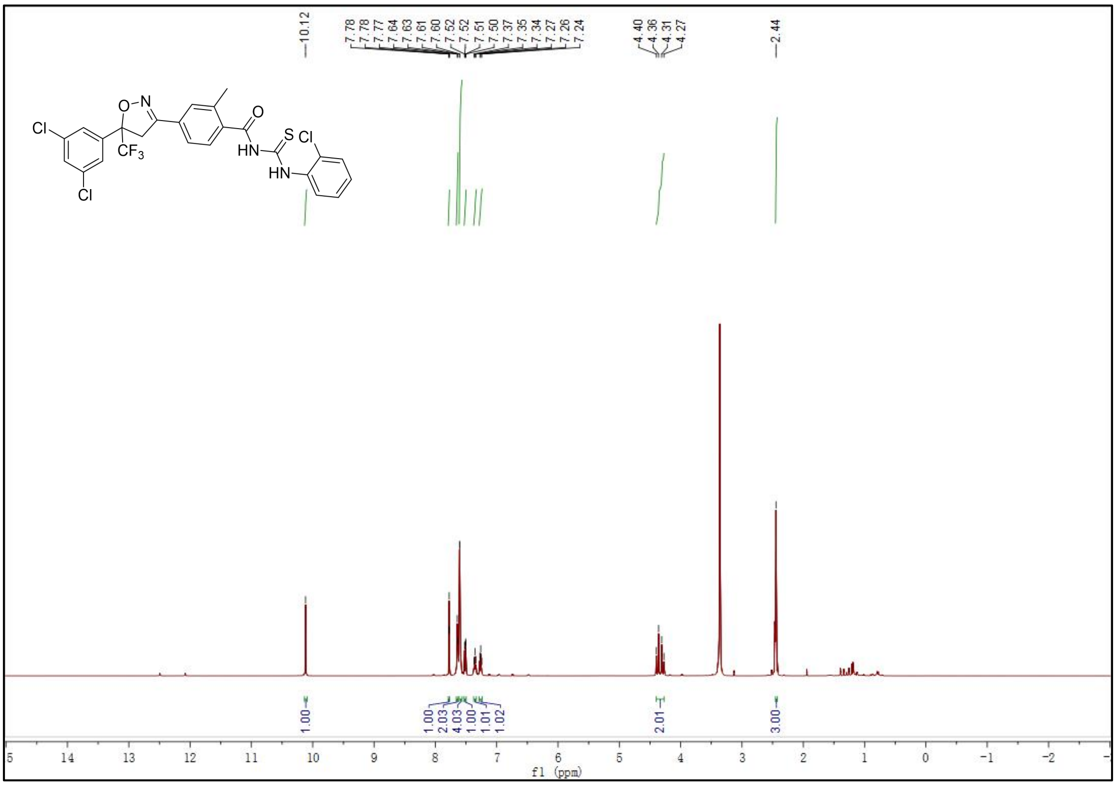

Figure 13. ^{1}H NMR spectrum of compound **5**.

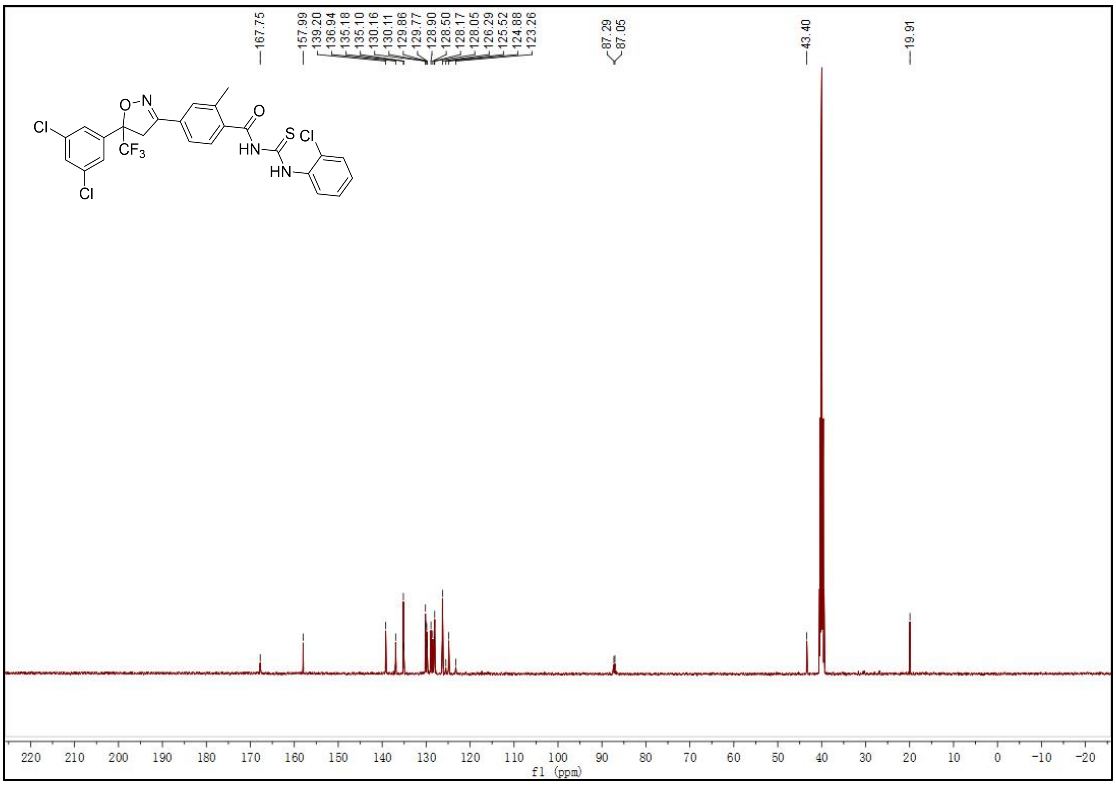

Figure 14. ^{13}C NMR spectrum of compound **5**.

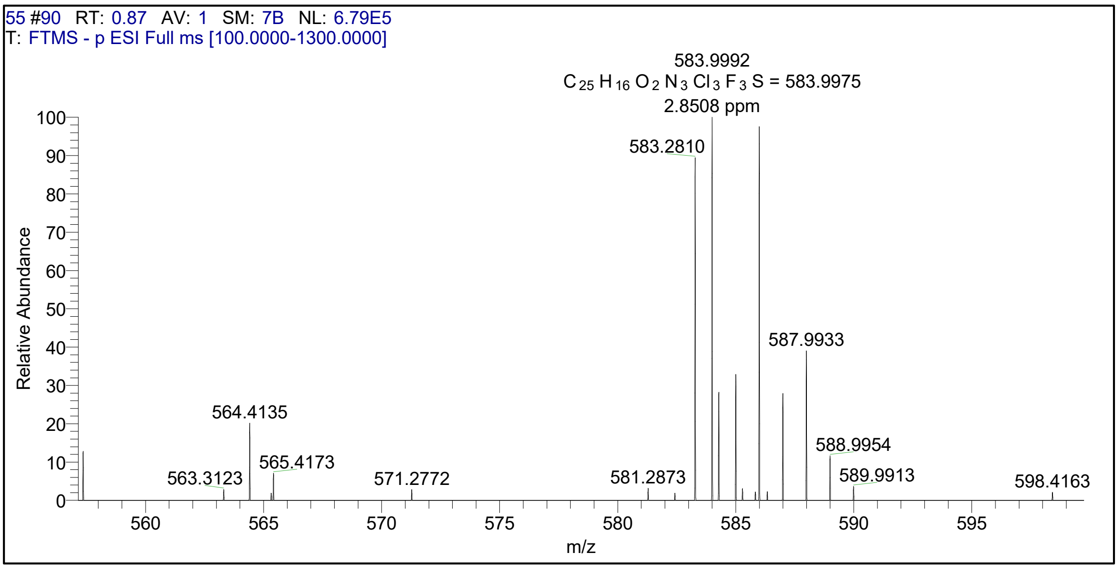

Figure 15. HRMS(ESI) of compound **5.**

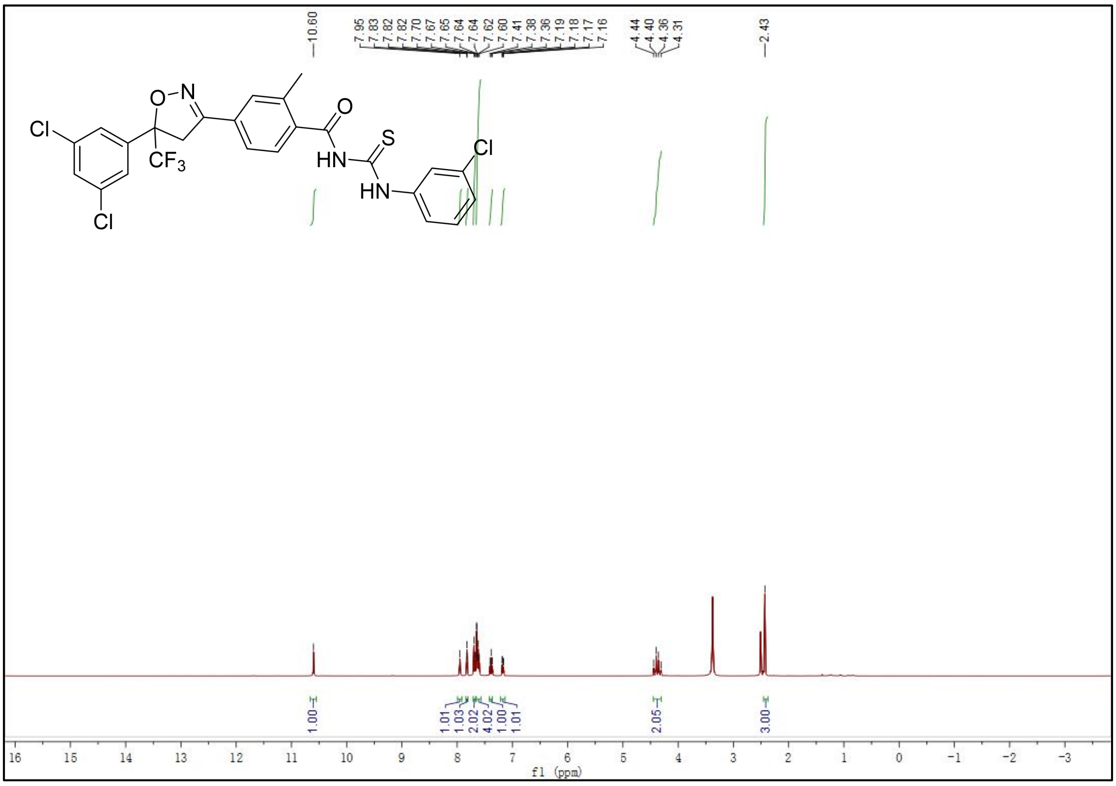

Figure 16. ^{1}H NMR spectrum of compound **6**.

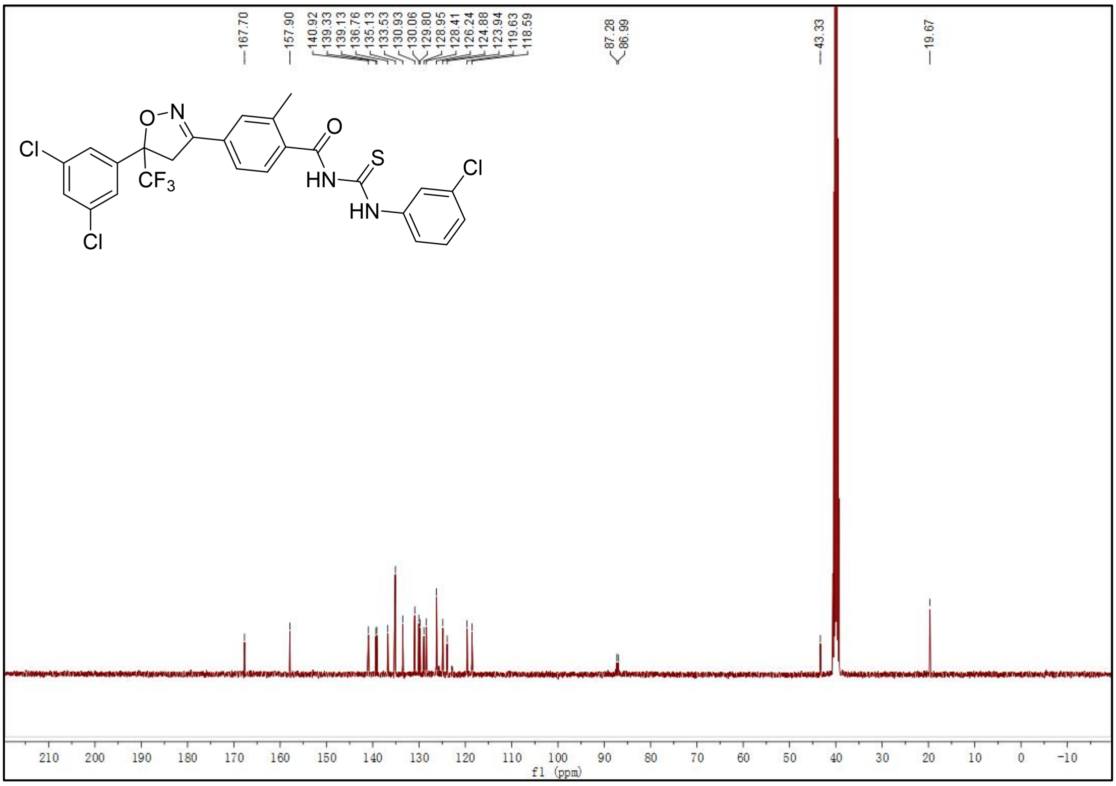

Figure 17. ^{13}C NMR spectrum of compound **6**.

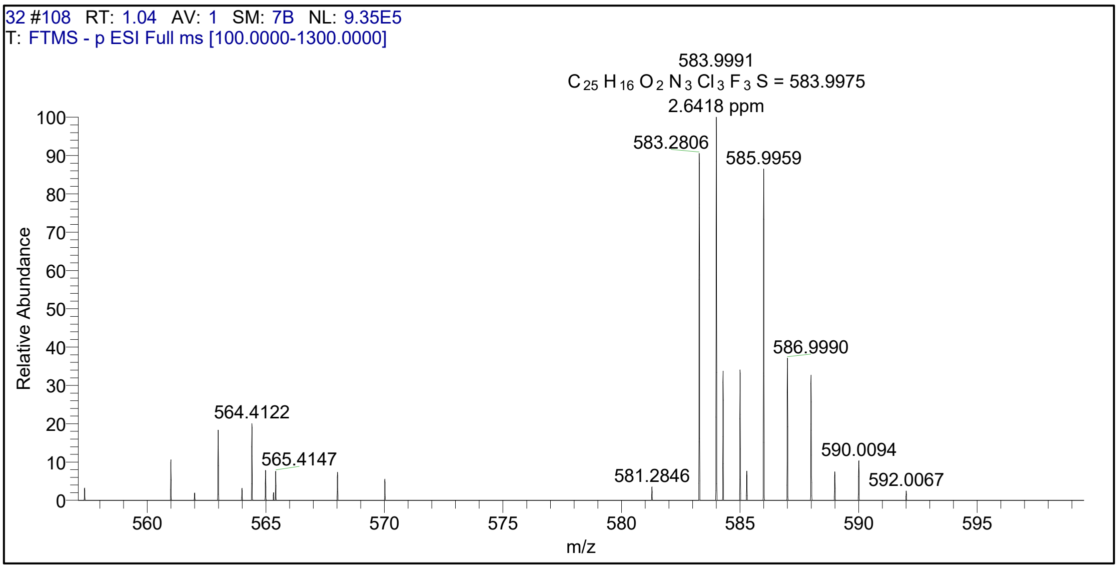

Figure 18. HRMS(ESI) of compound **6.**

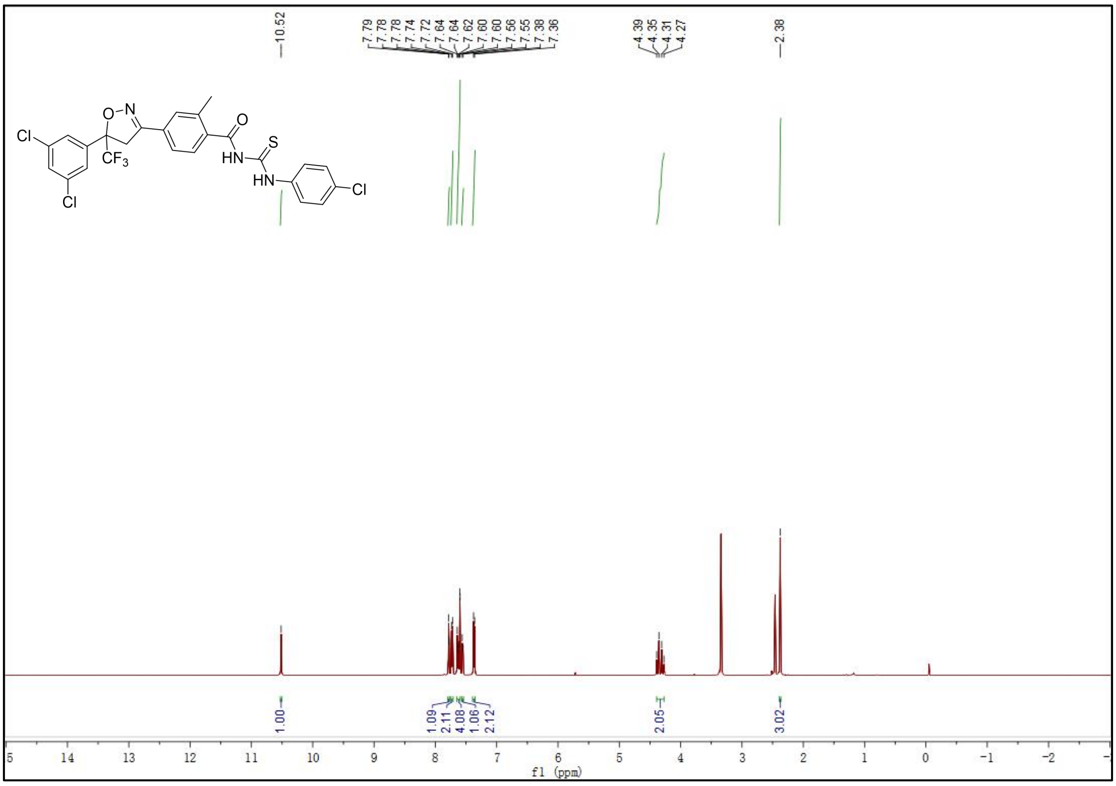

Figure 19. ^{1}H NMR spectrum of compound **7**.

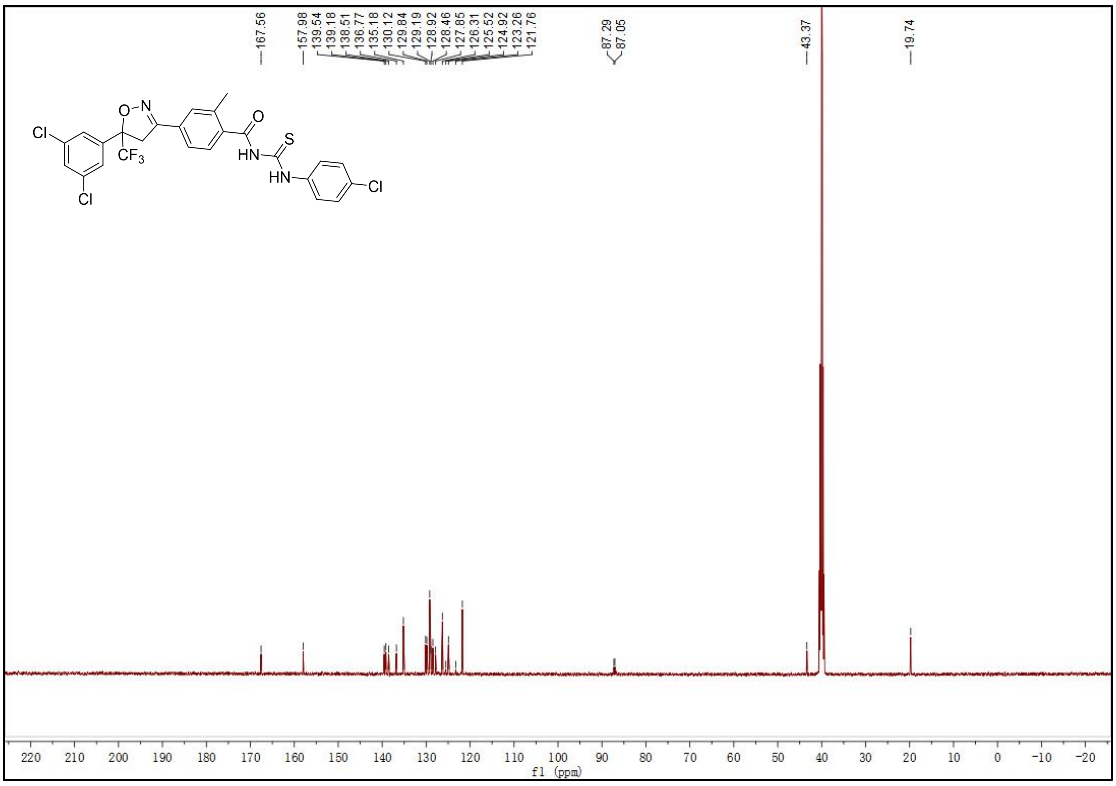

Figure 20. ^{13}C NMR spectrum of compound **7**.

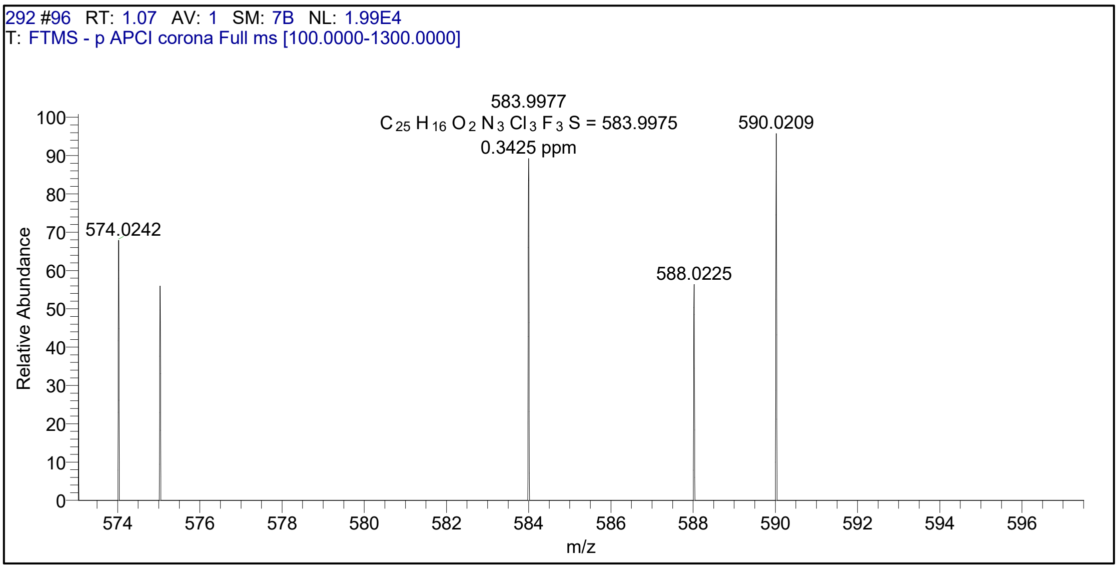

Figure 21. HRMS(ESI) of compound **7.**

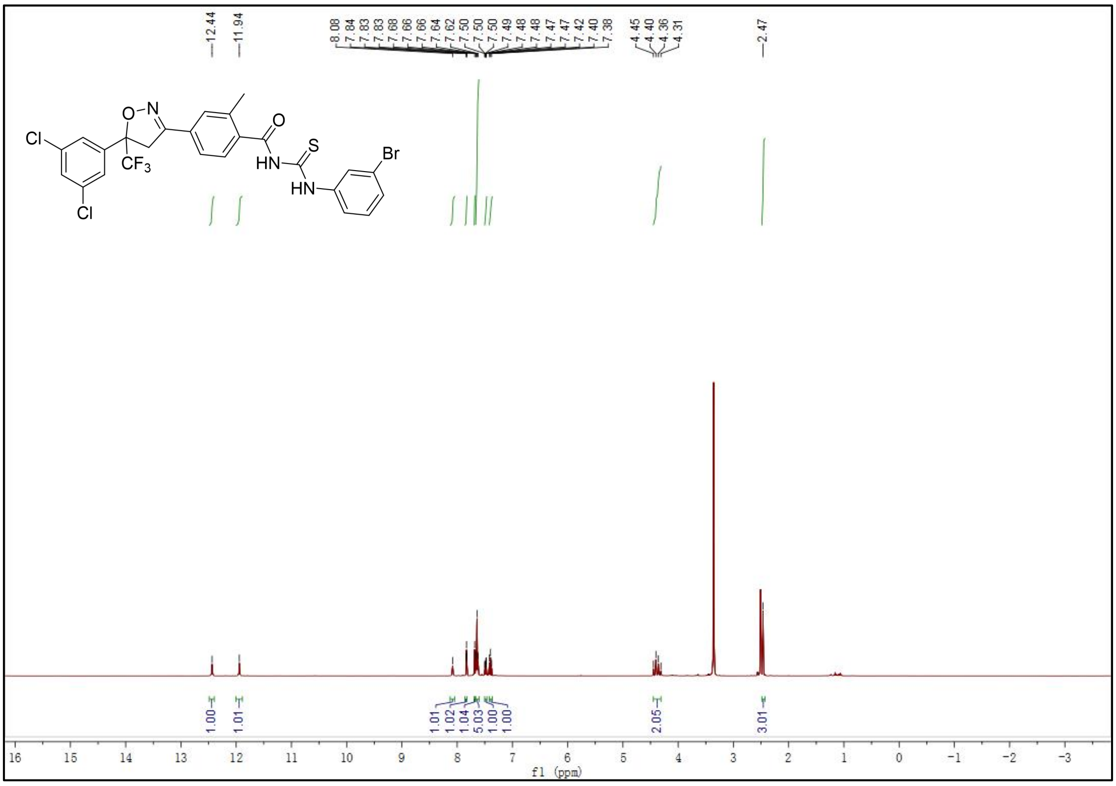

Figure 22. ^{1}H NMR spectrum of compound **8**.

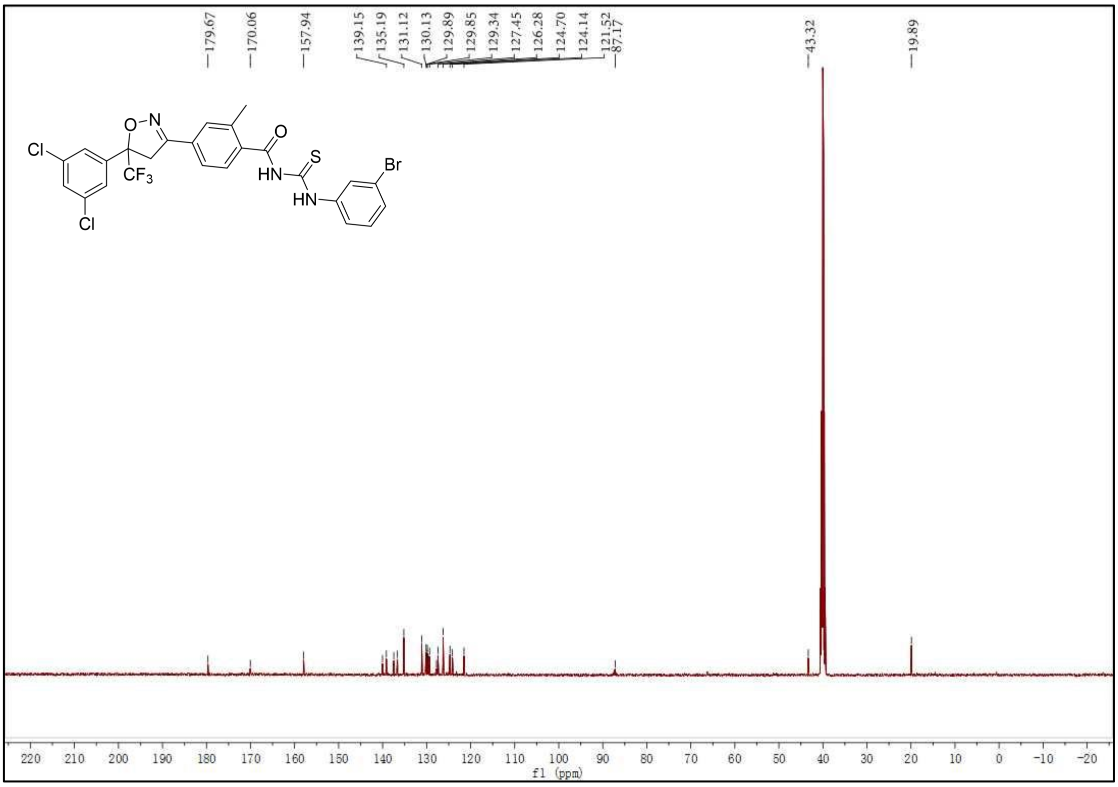

Figure 23. ^{13}C NMR spectrum of compound **8**.

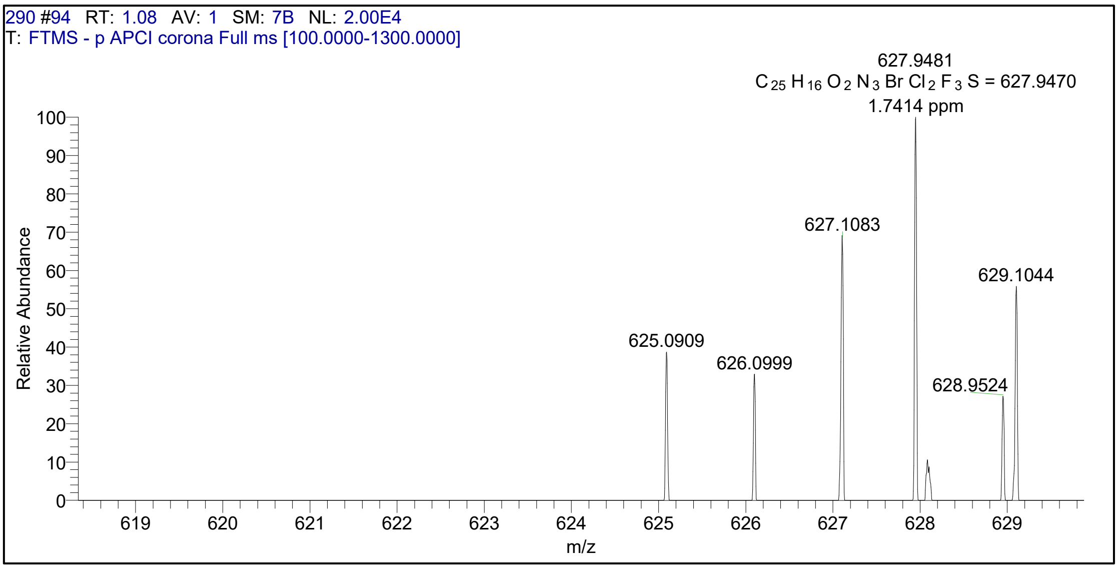

Figure 24. HRMS(ESI) of compound **8.**

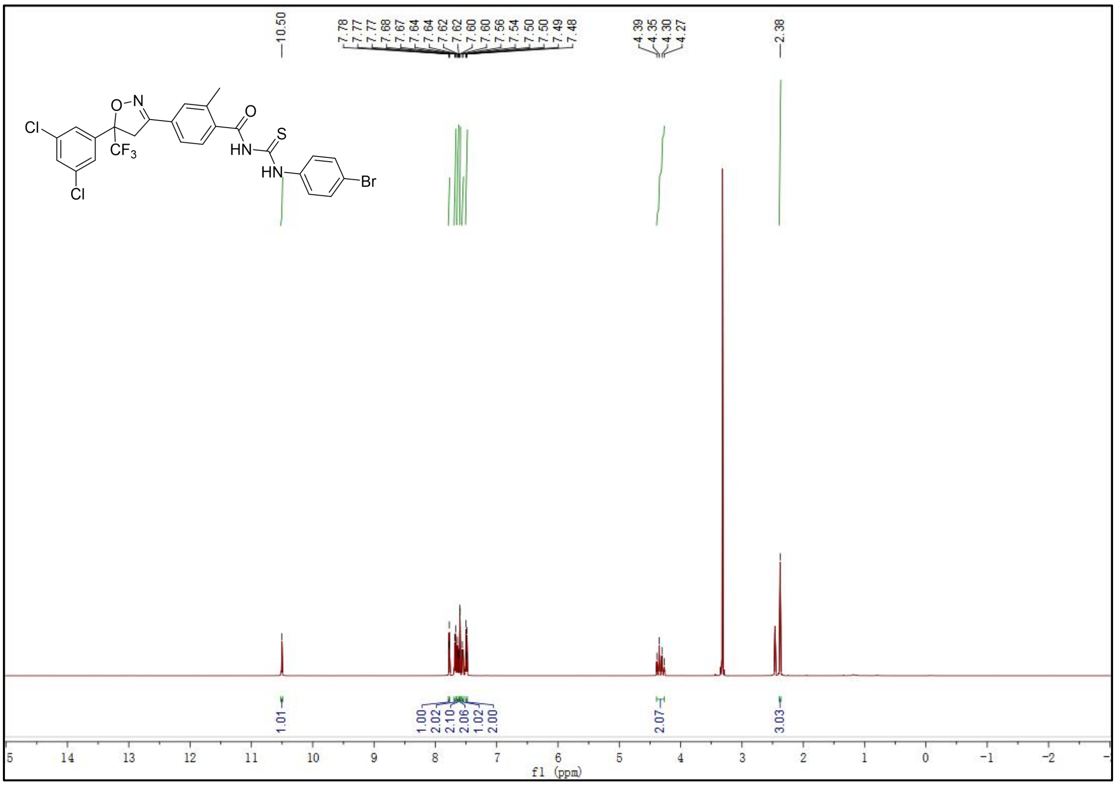

Figure 25. ^{1}H NMR spectrum of compound **9**.

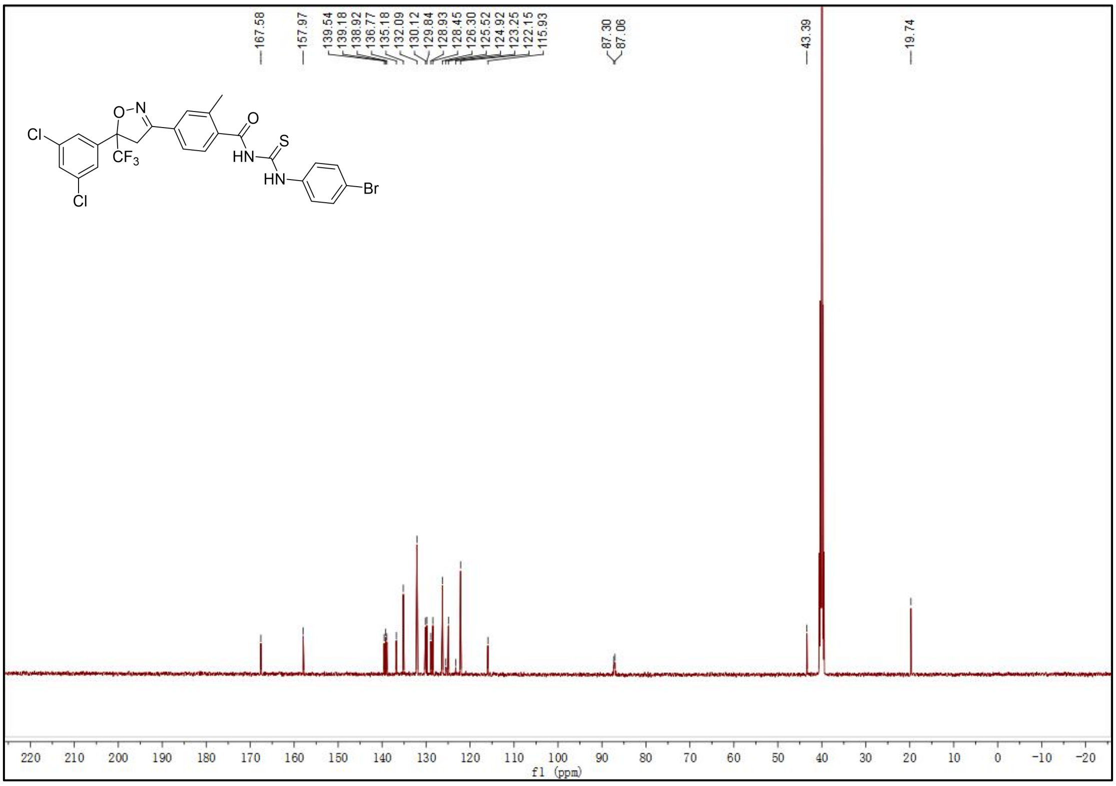

Figure 26. ^{13}C NMR spectrum of compound **9**.

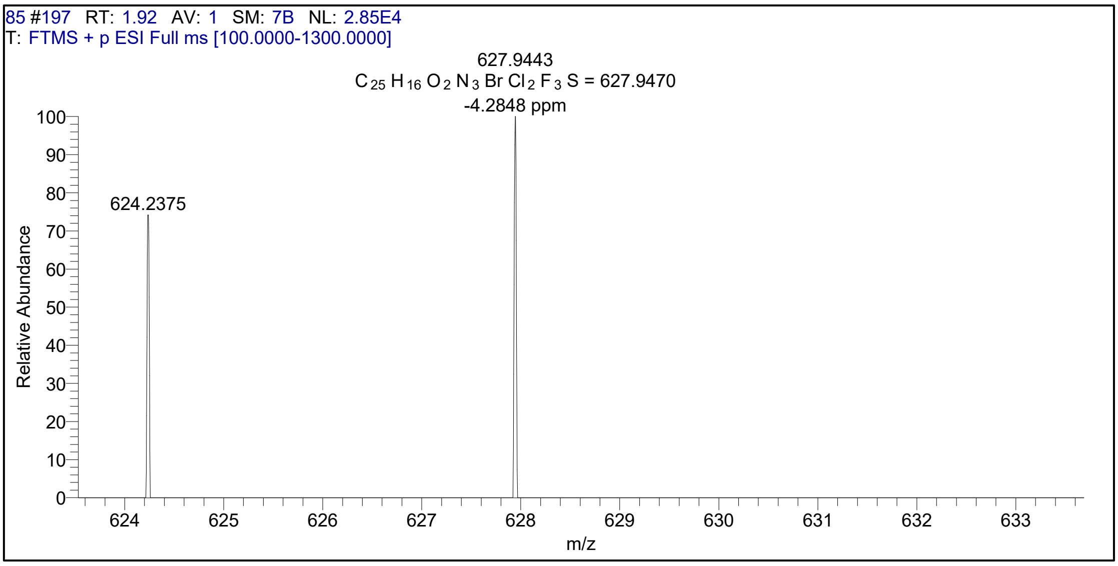

Figure 27. HRMS(ESI) of compound **9.**

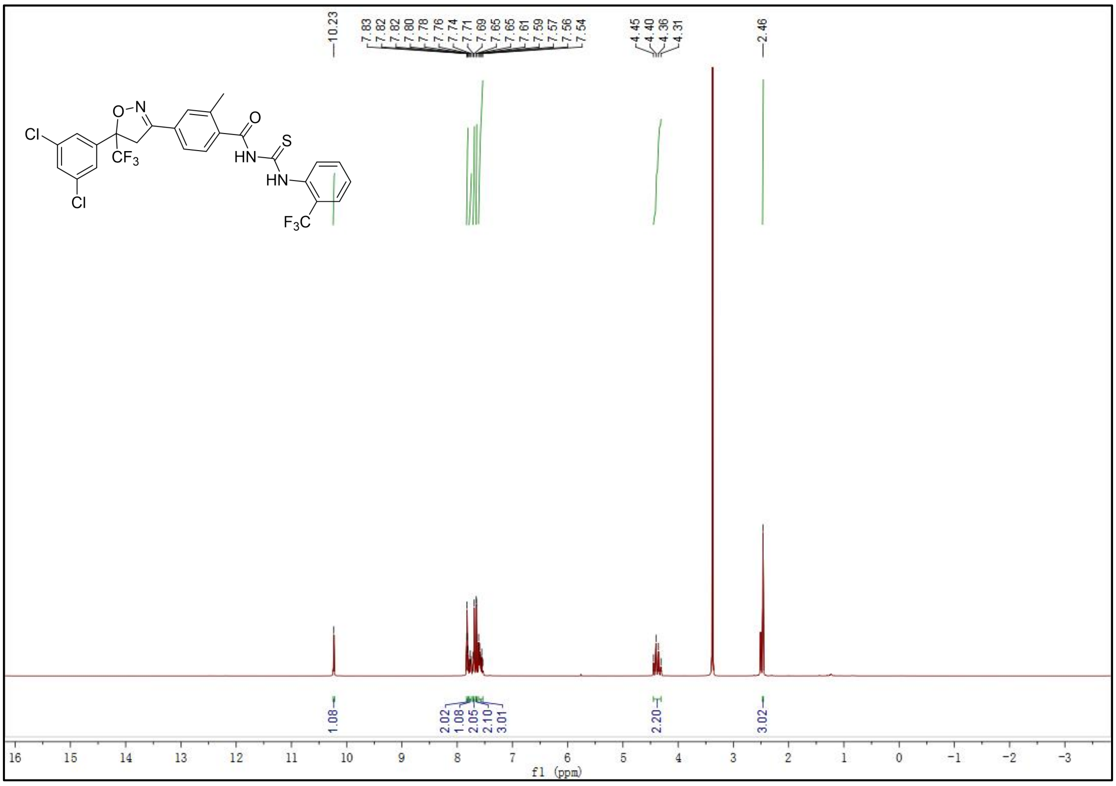

Figure 28. ^{1}H NMR spectrum of compound **10**.

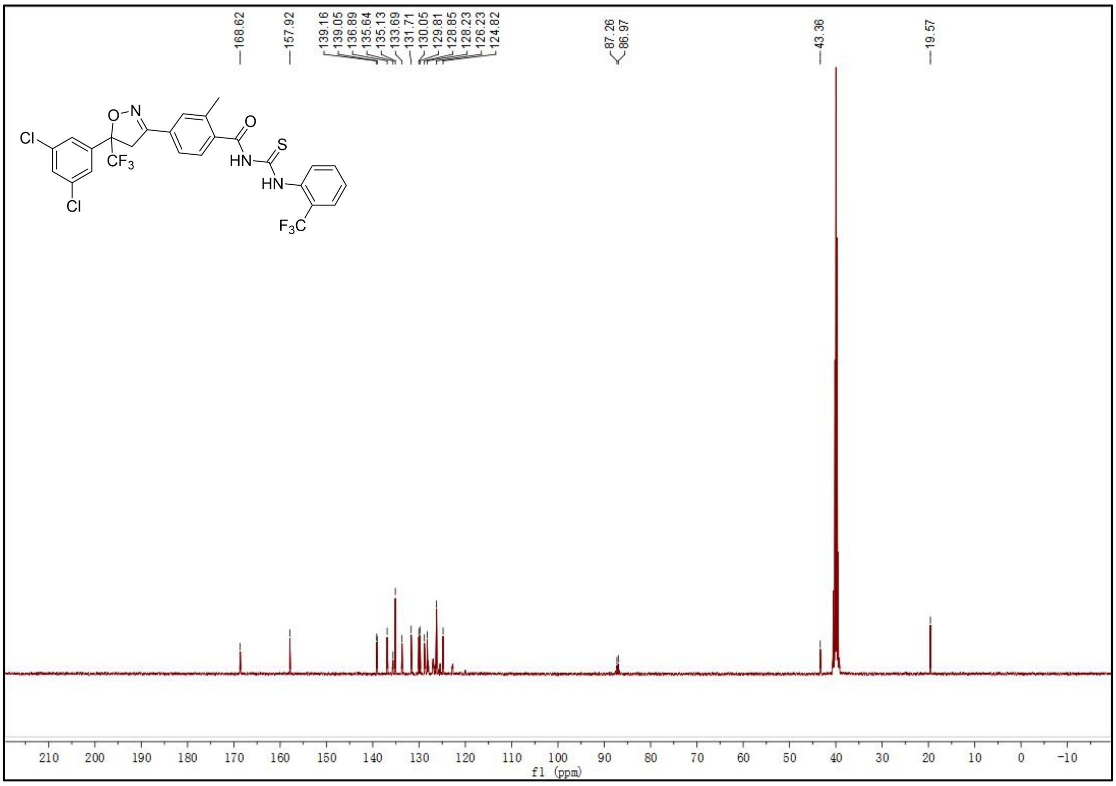

Figure 29. ^{13}C NMR spectrum of compound **10**.

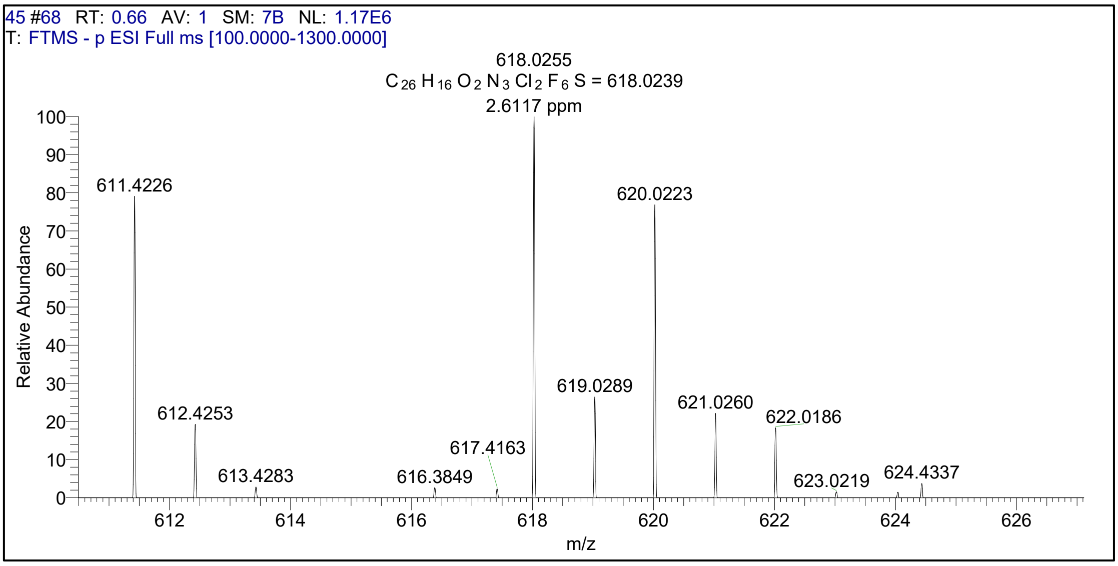

Figure 30. HRMS(ESI) of compound **10.**

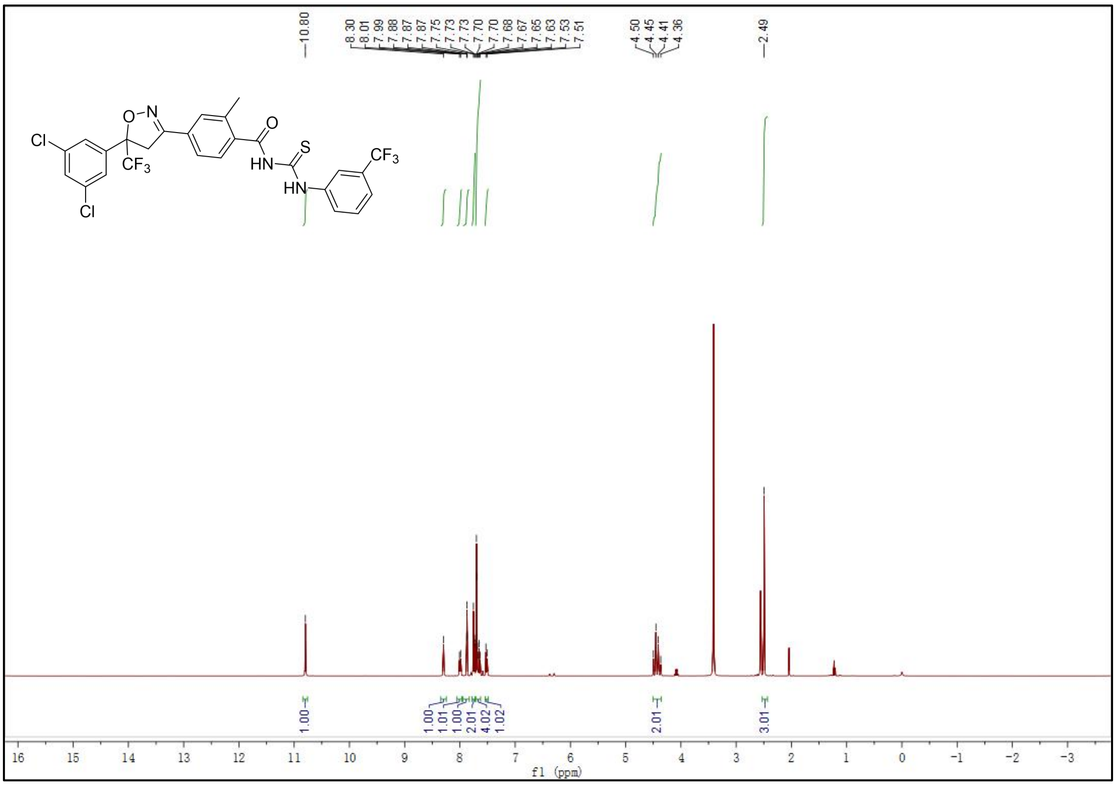

Figure 31. ^{1}H NMR spectrum of compound **11**.

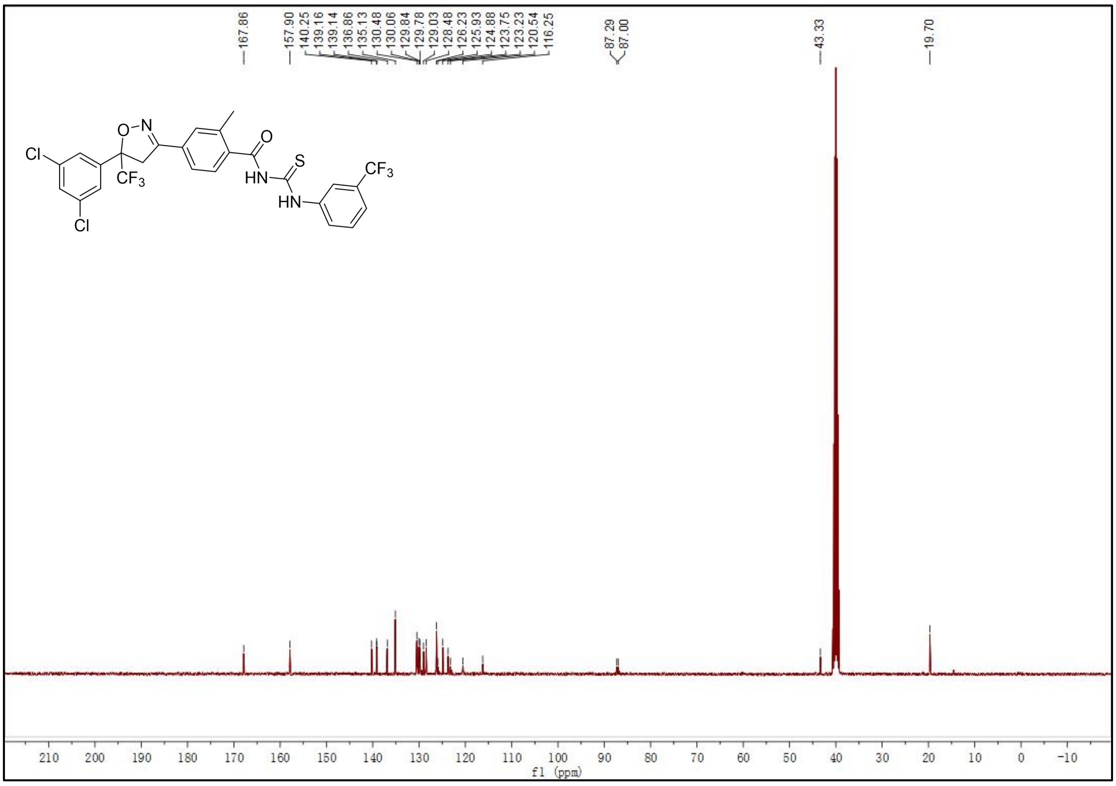

Figure 32. ^{13}C NMR spectrum of compound **11**.

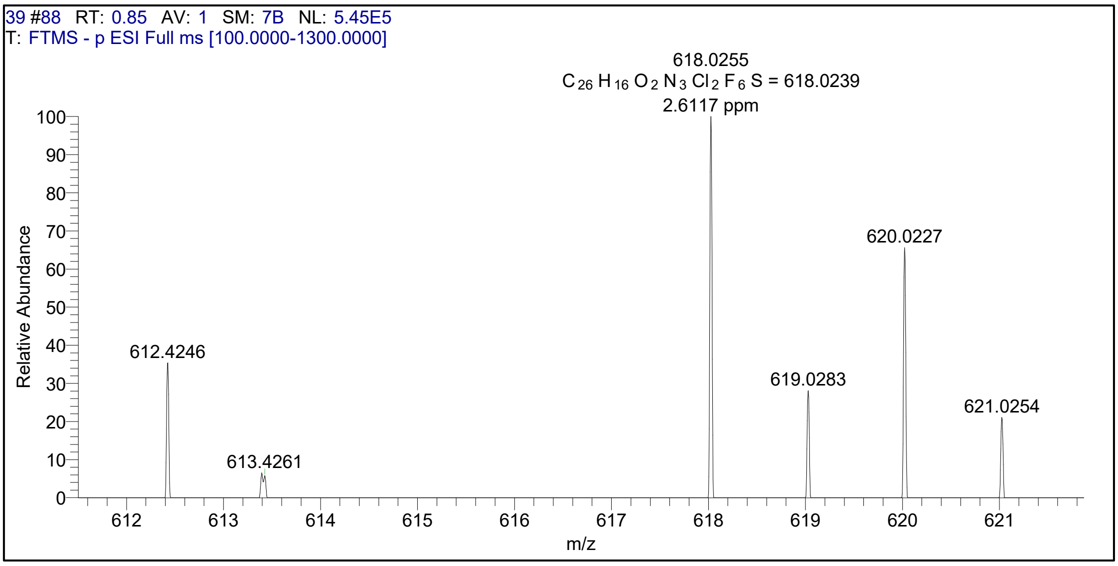

Figure 33. HRMS(ESI) of compound **11.**

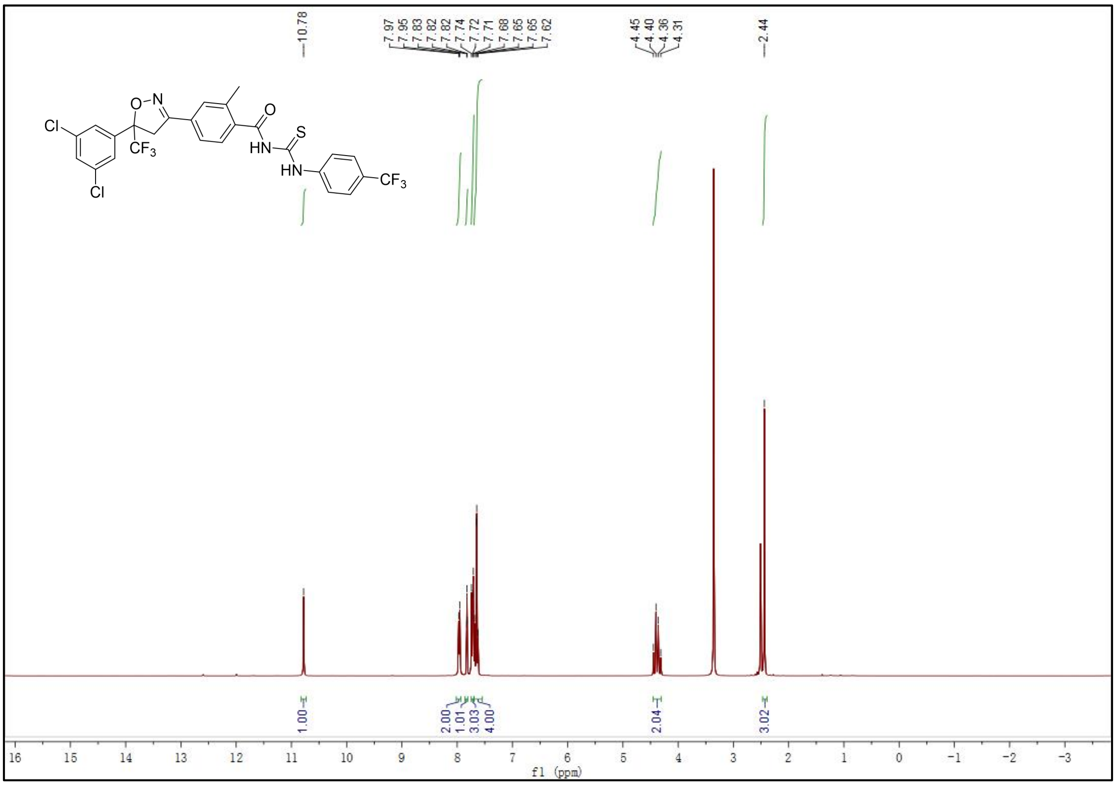

Figure 34. ^{1}H NMR spectrum of compound **12**.

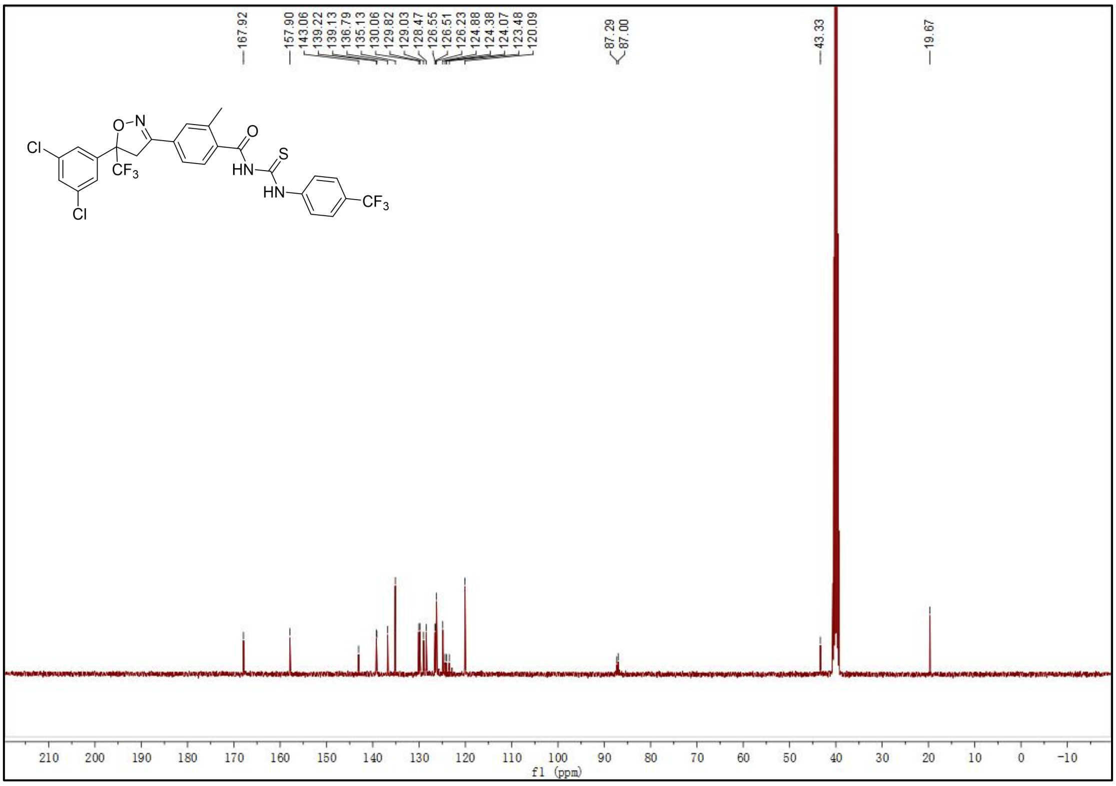

Figure 35. ^{13}C NMR spectrum of compound **12**.

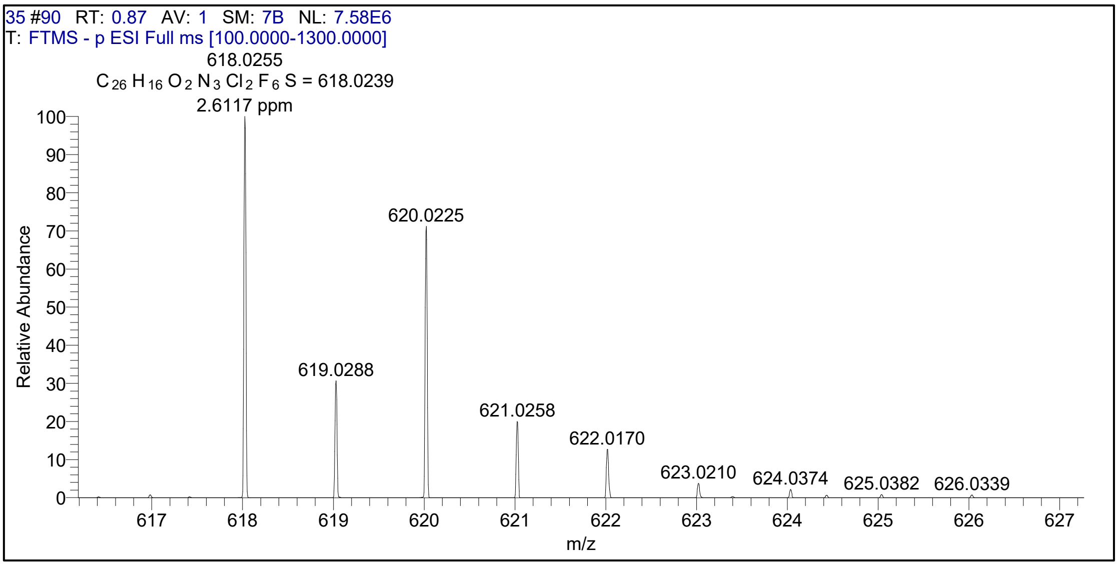

Figure 36. HRMS(ESI) of compound **12.**

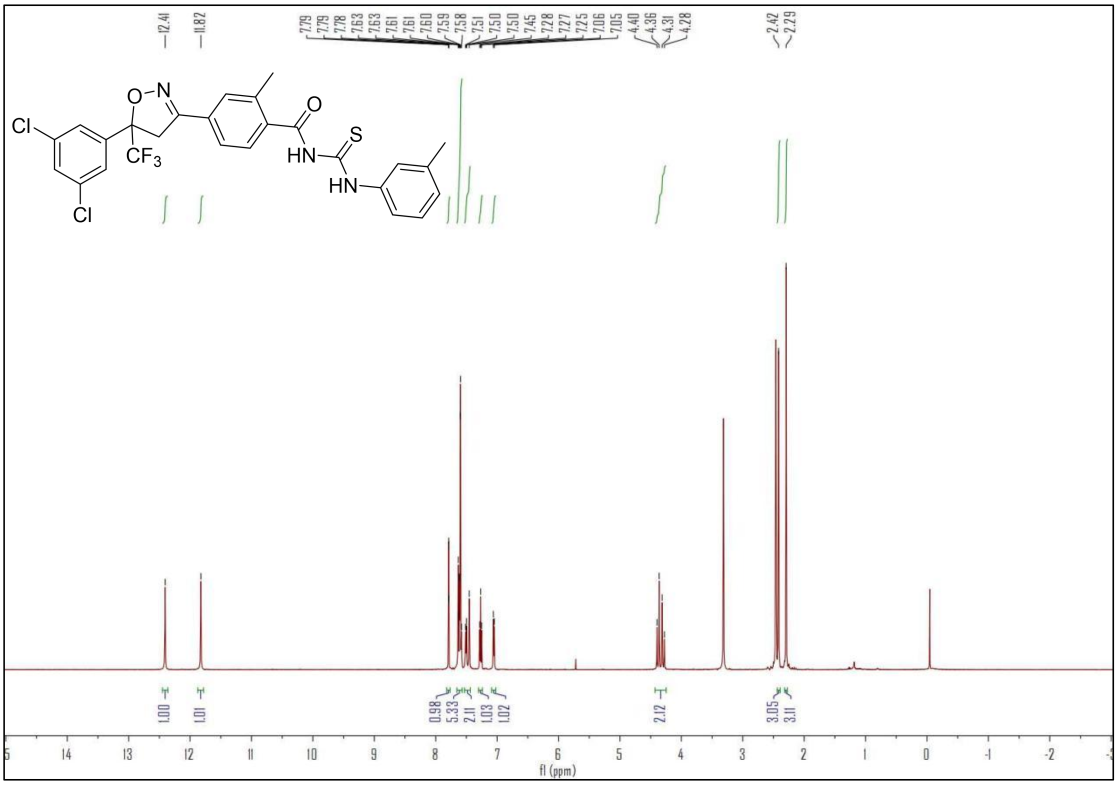

Figure 37. ^{1}H NMR spectrum of compound **13**.

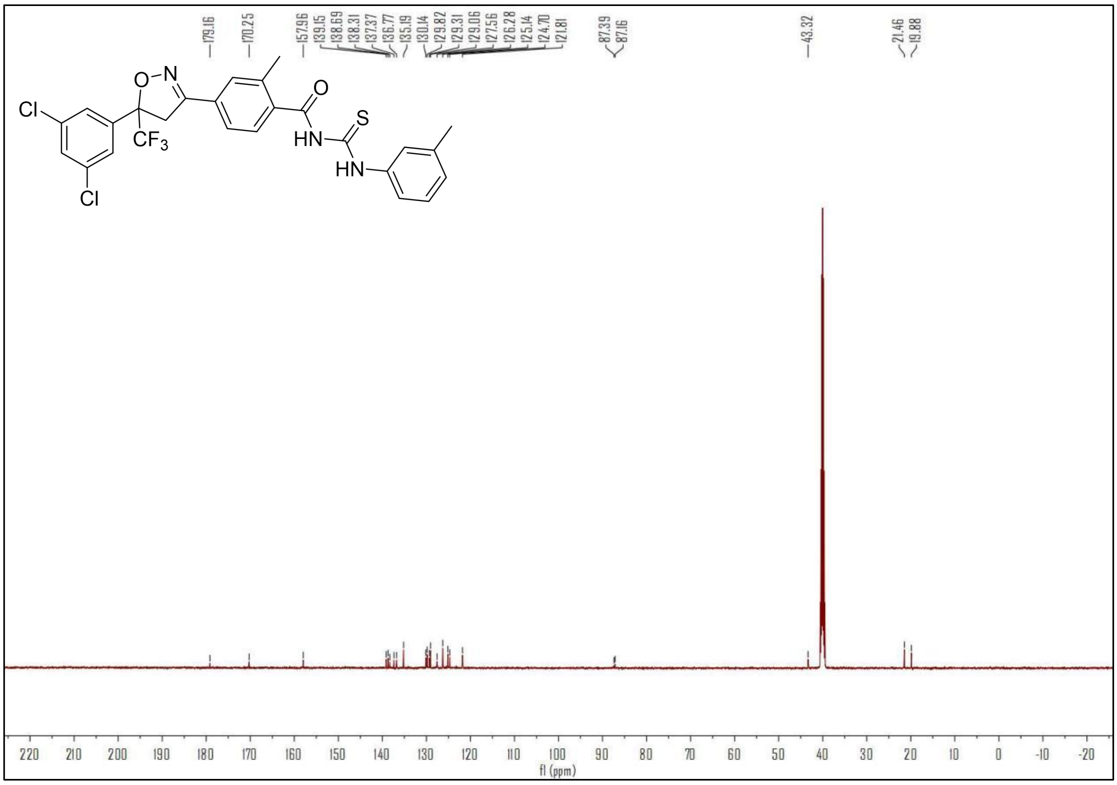

Figure 38. ^{13}C NMR spectrum of compound **13**.

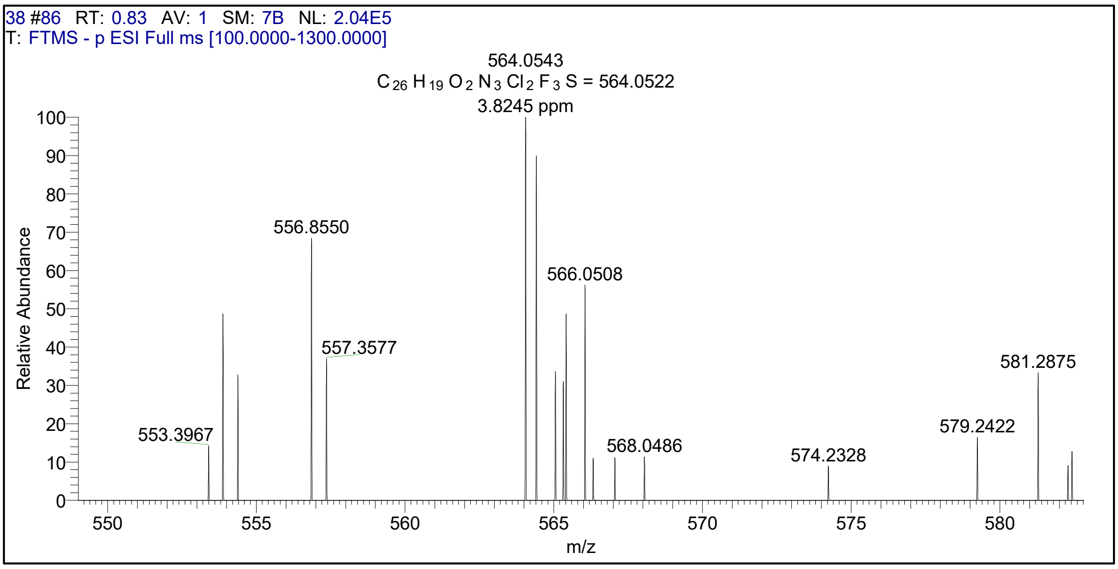

Figure 39. HRMS(ESI) of compound **13.**

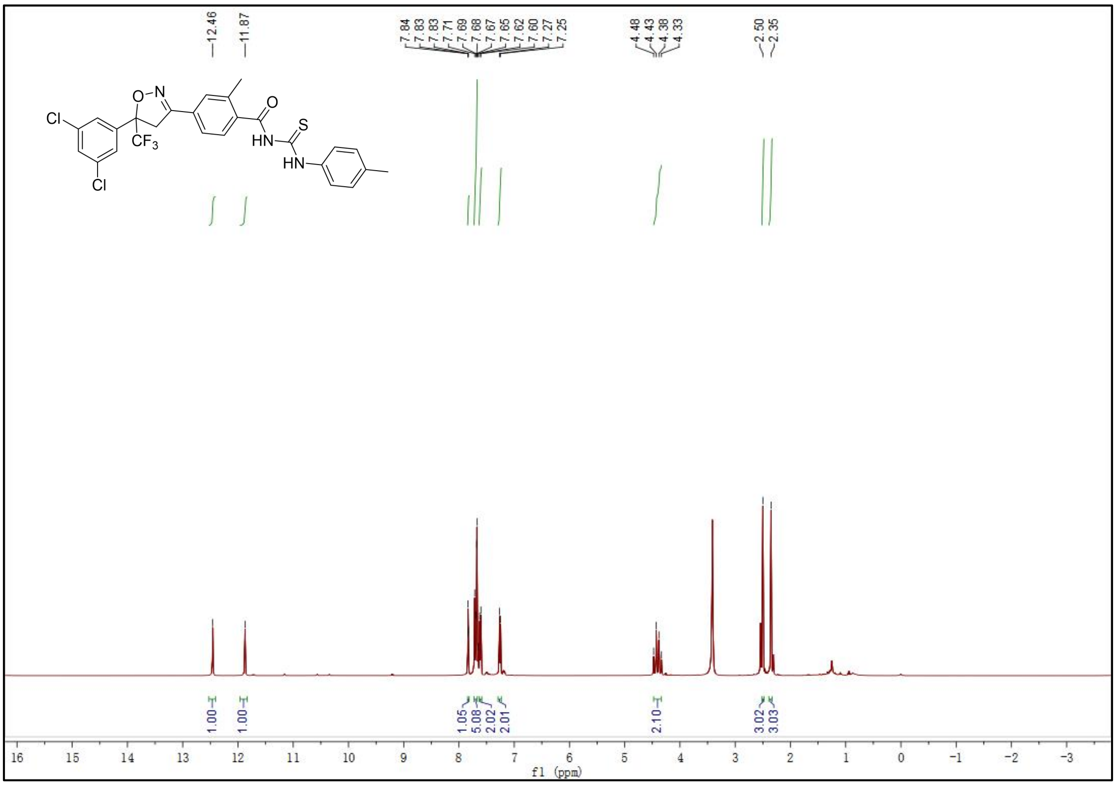

Figure 40. ^{1}H NMR spectrum of compound **14**.

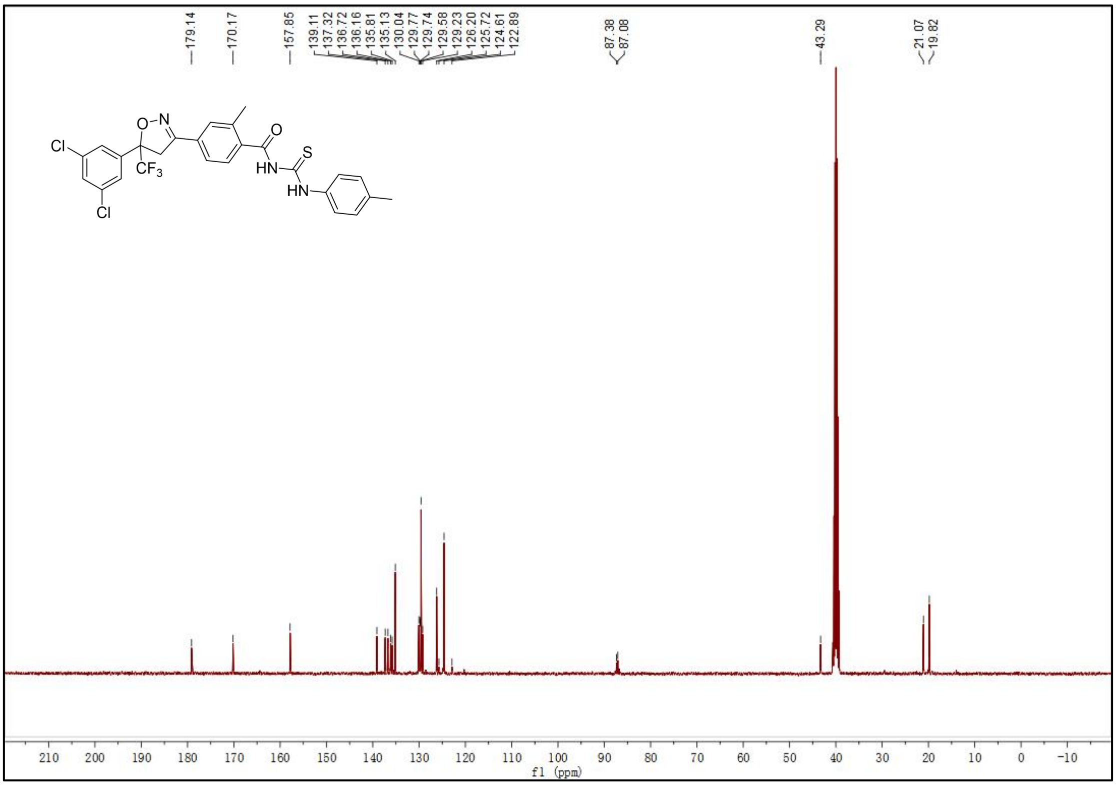

Figure 41. ^{13}C NMR spectrum of compound **14**.

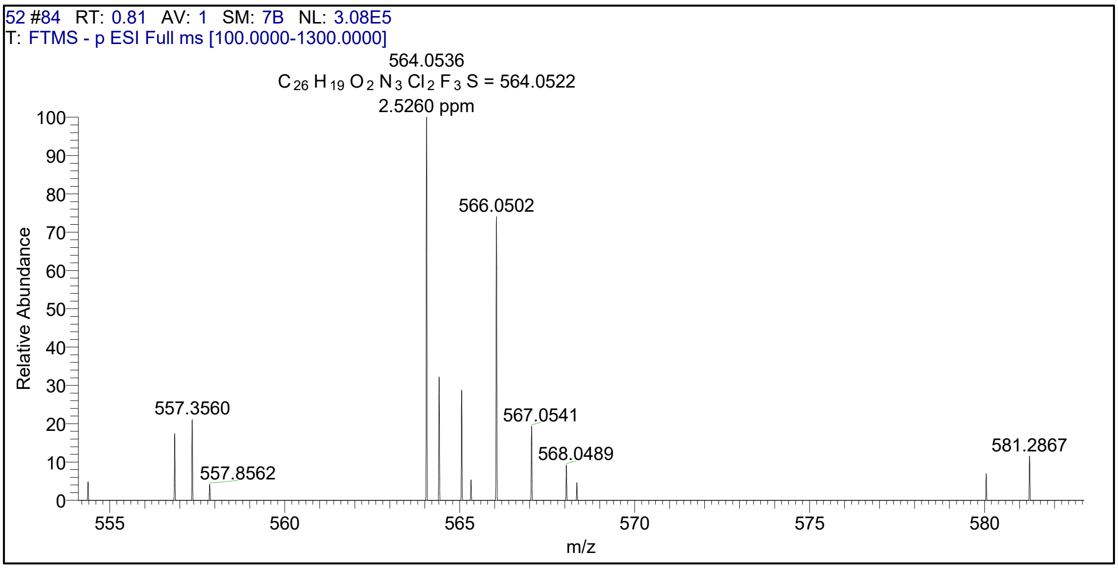

Figure 42. HRMS(ESI) of compound **14.**

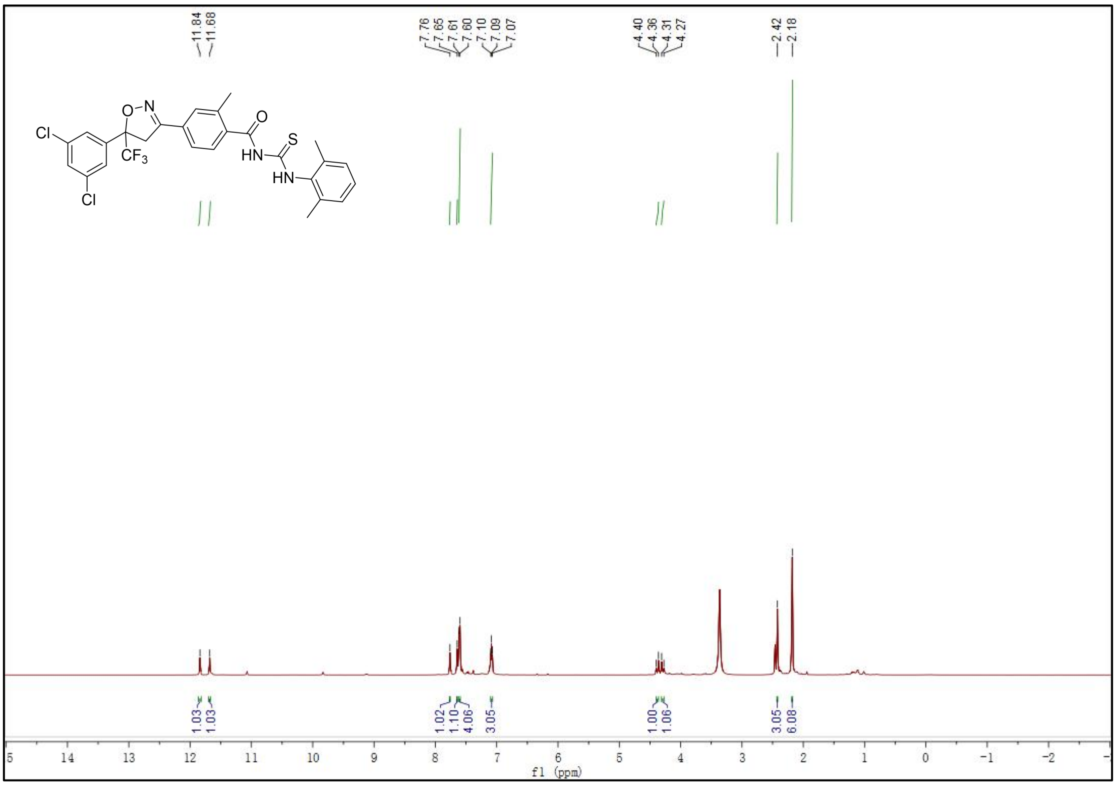

Figure 43. ^{1}H NMR spectrum of compound **15**.

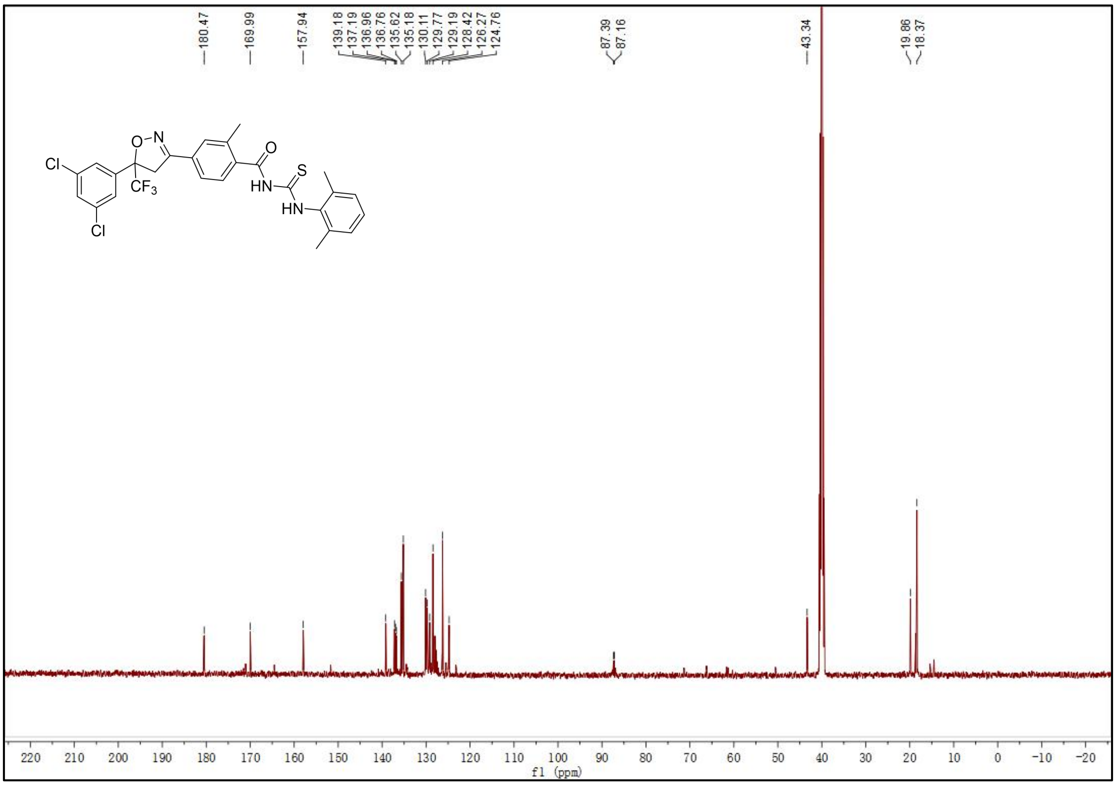

Figure 44. ^{13}C NMR spectrum of compound **15**.

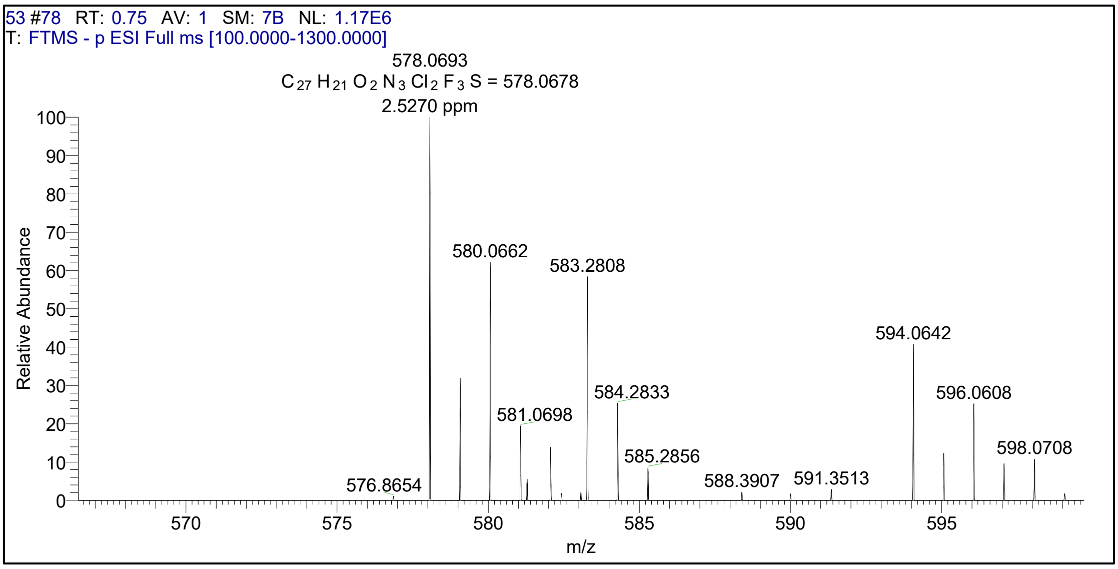

Figure 45. HRMS(ESI) of compound **15.**

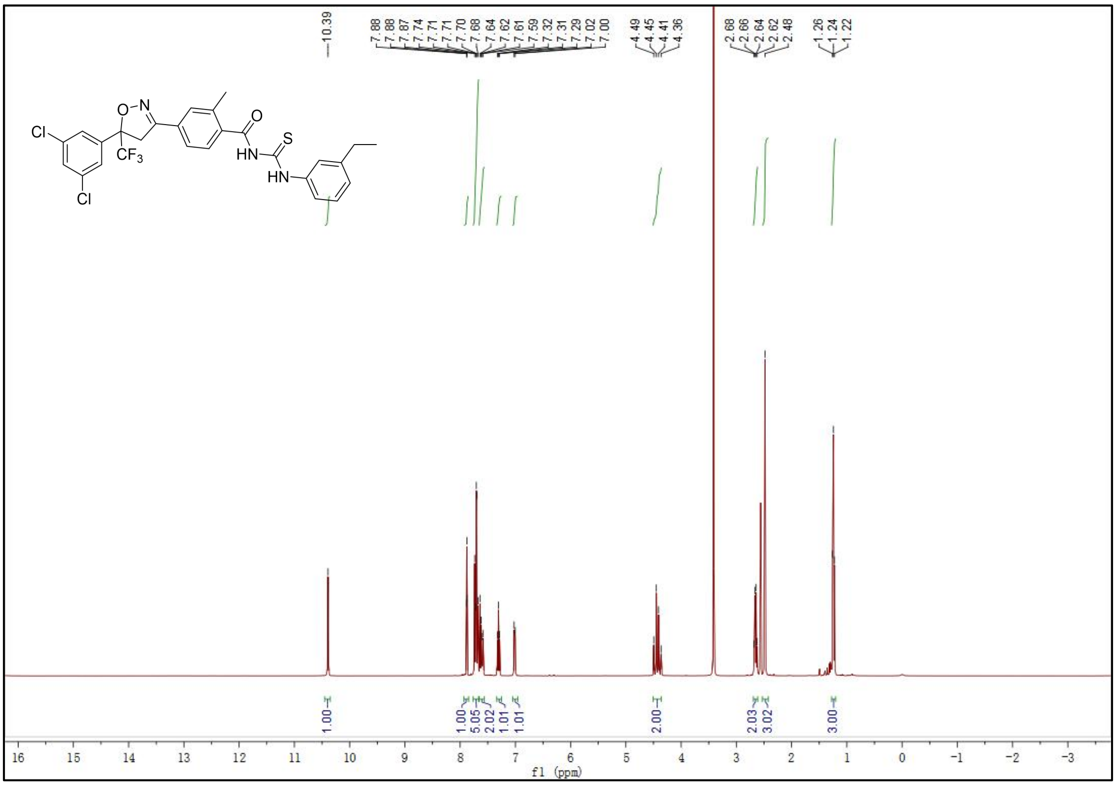

Figure 46. ^{1}H NMR spectrum of compound **16**.

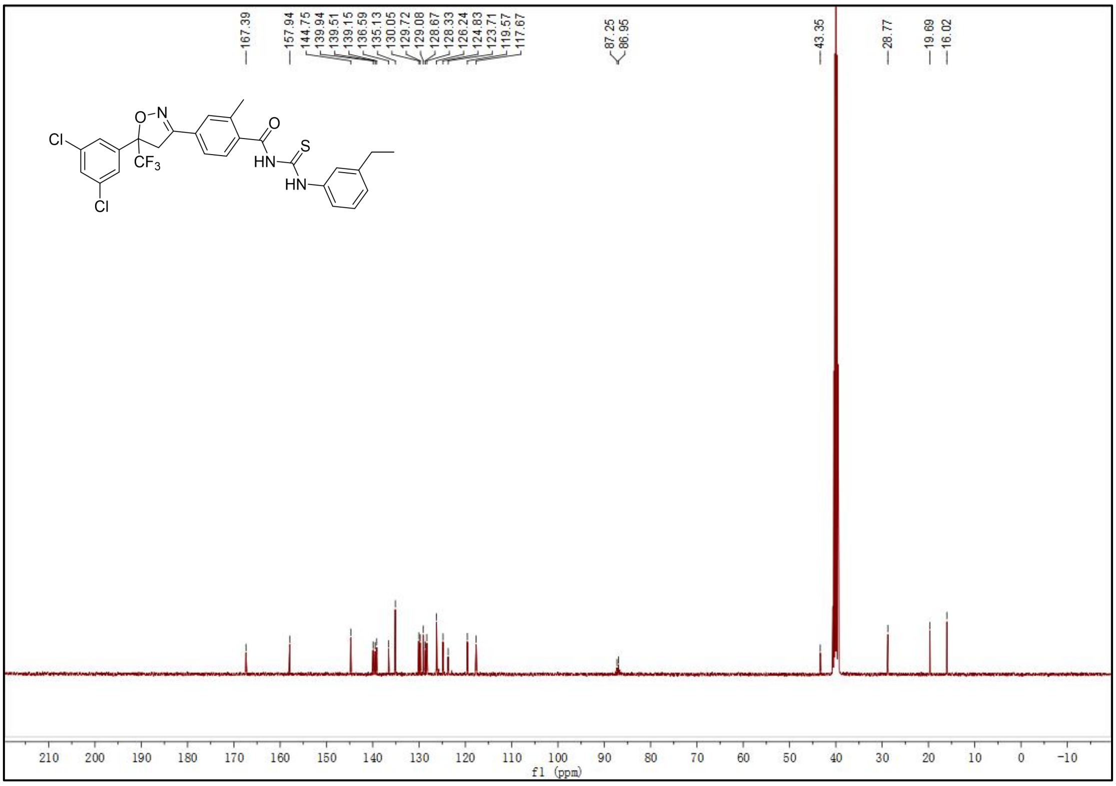

Figure 47. ^{13}C NMR spectrum of compound **16**.

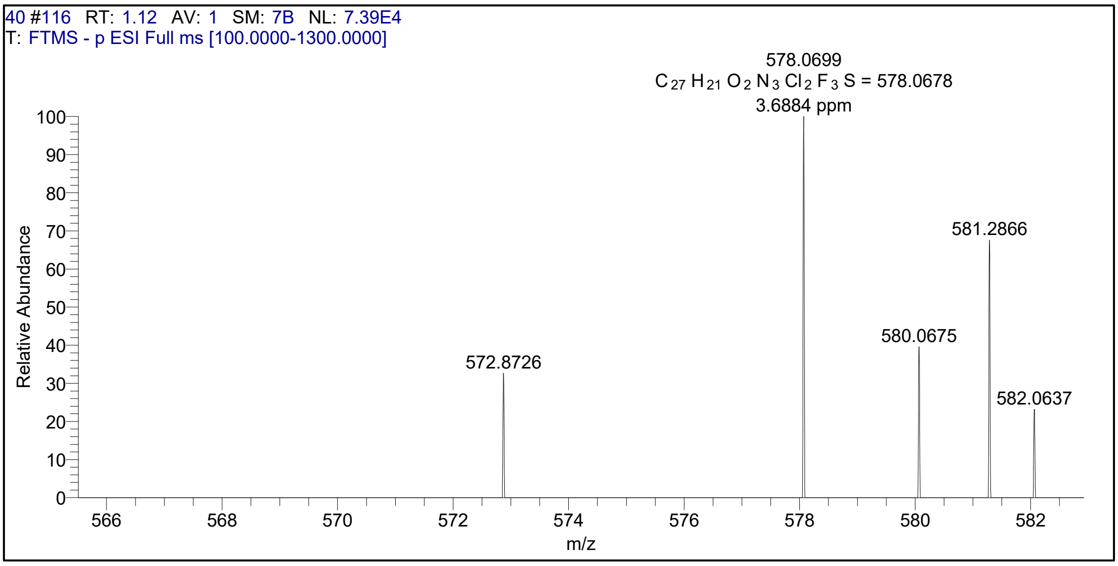

Figure 48. HRMS(ESI) of compound **16.**

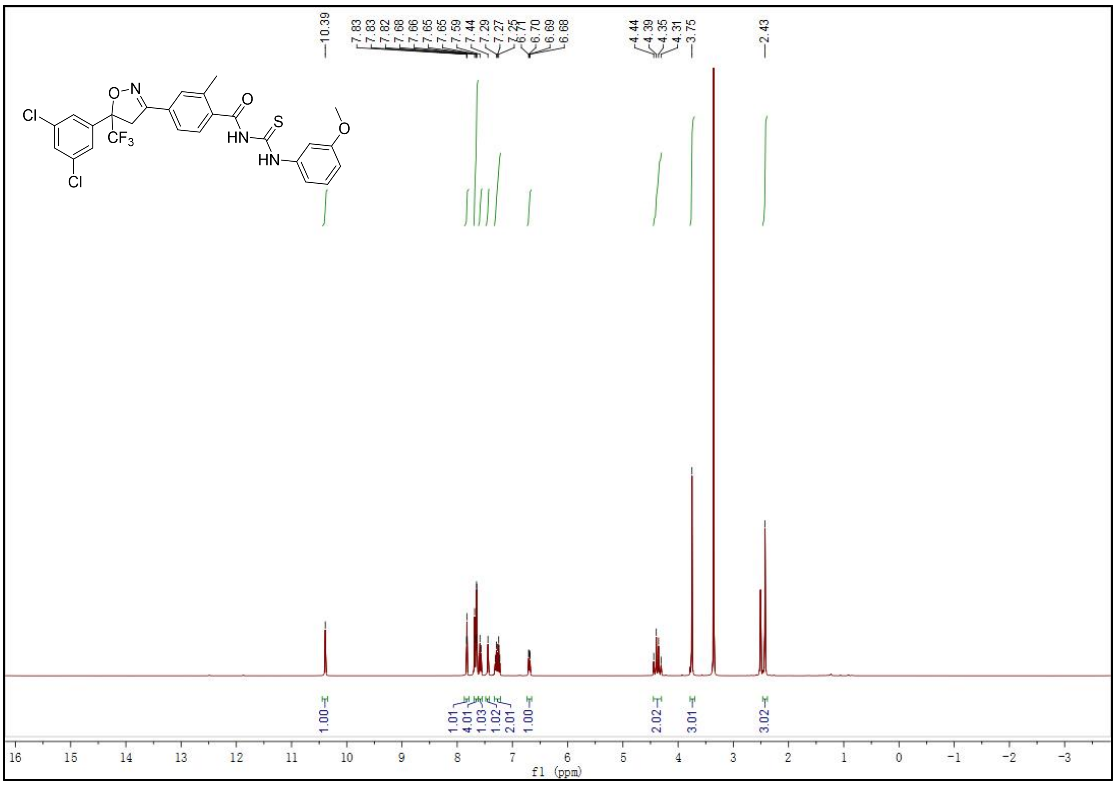

Figure 49. ^{1}H NMR spectrum of compound **17**.

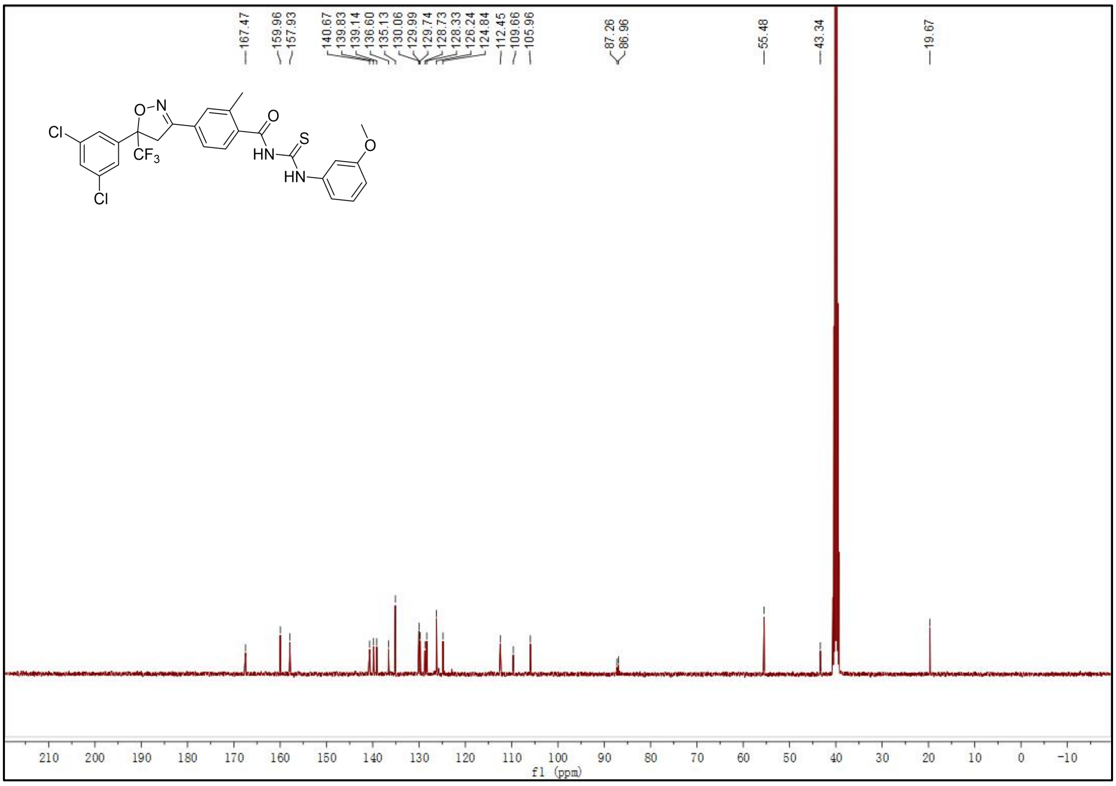

Figure 50. ^{13}C NMR spectrum of compound **17**.

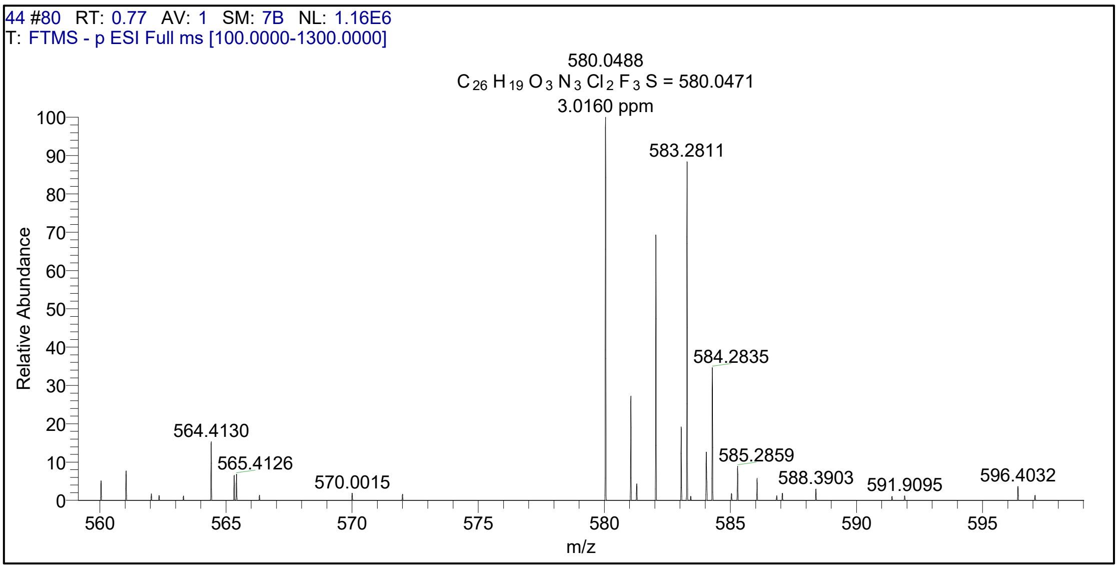

Figure 51. HRMS(ESI) of compound **17.**

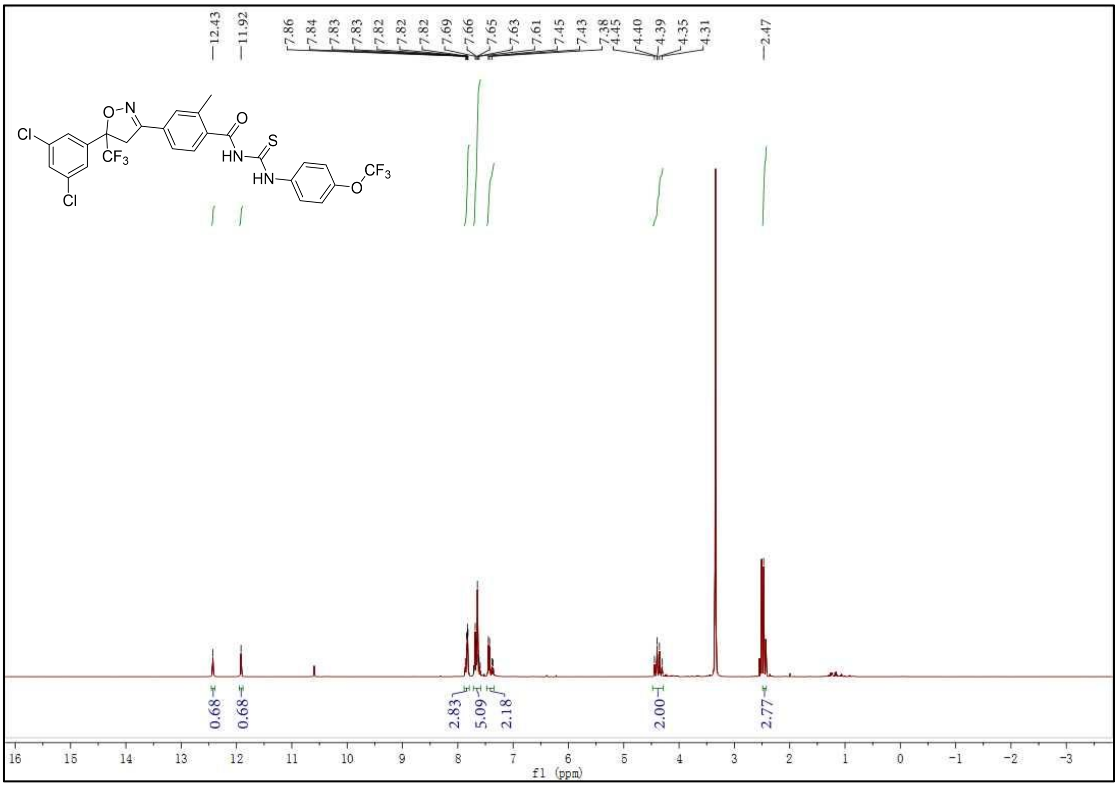

Figure 52. ^{1}H NMR spectrum of compound **18**.

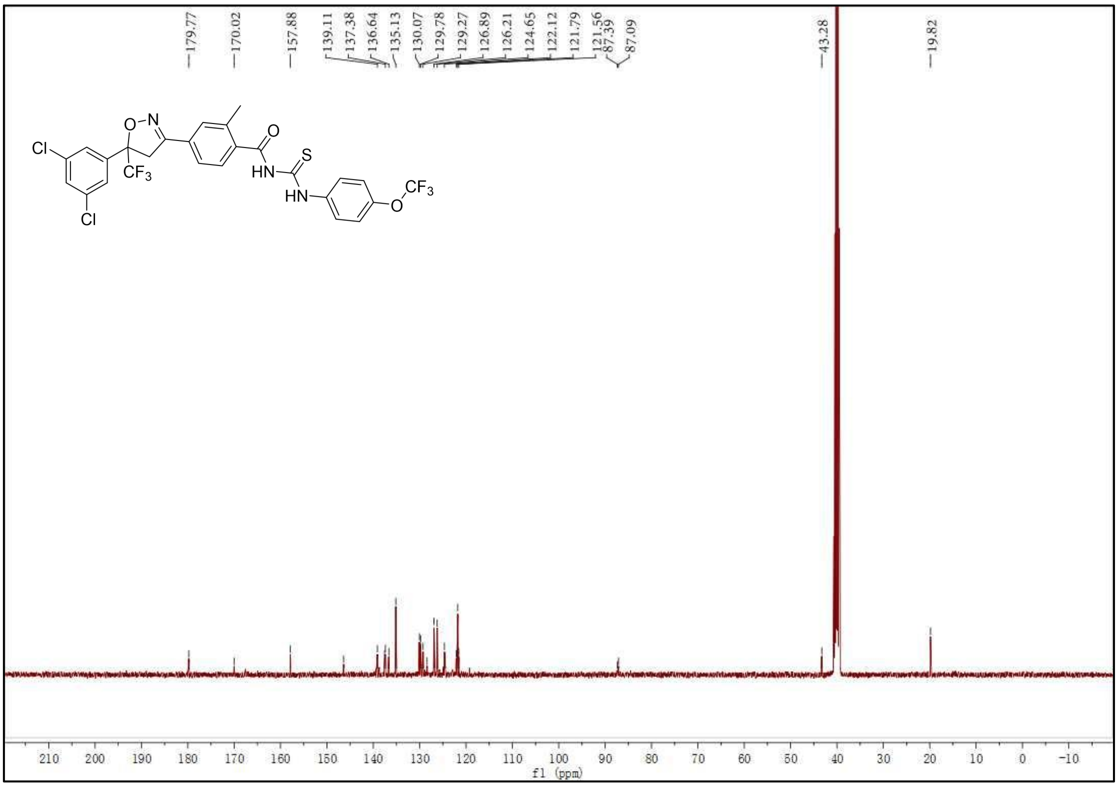

Figure 53. ^{13}C NMR spectrum of compound **18**.

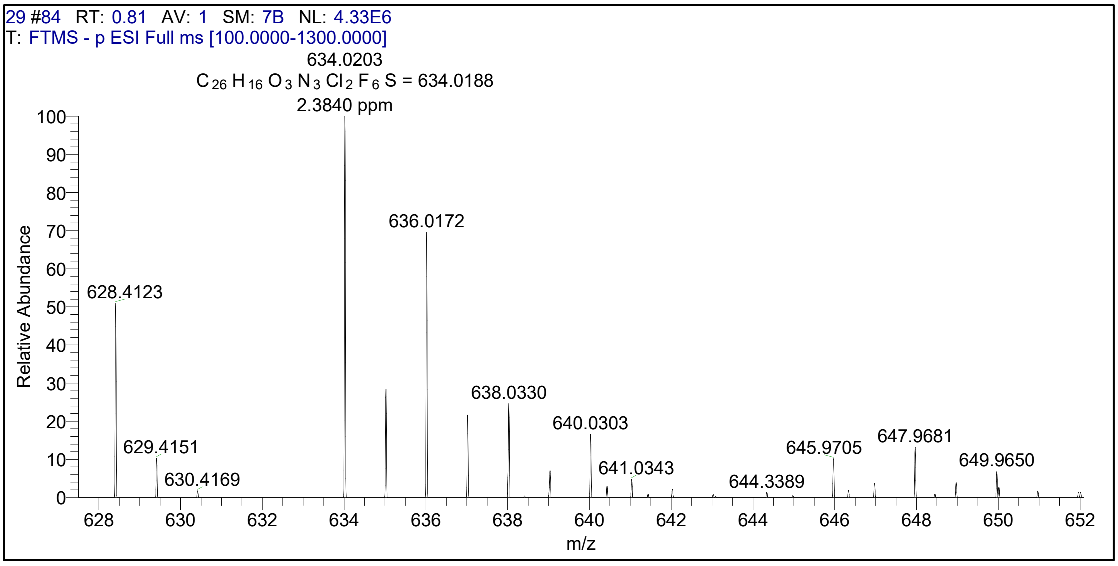

Figure 54. HRMS(ESI) of compound **18.**

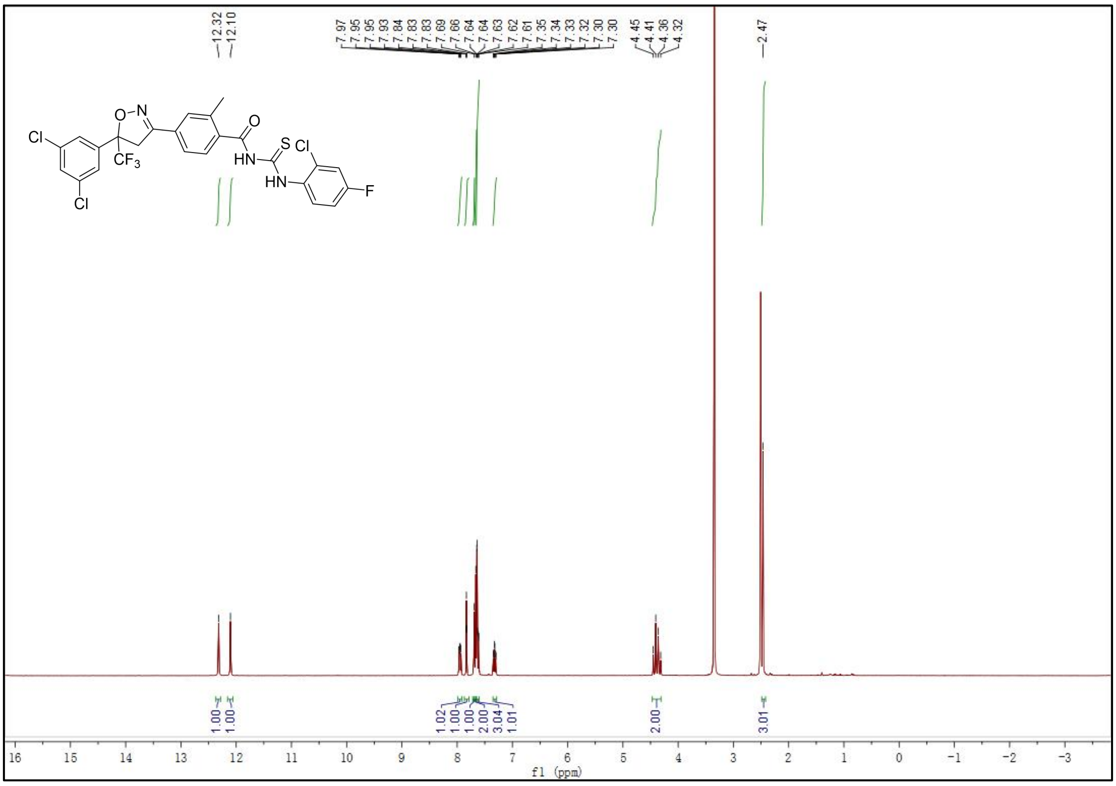

Figure 55. ^{1}H NMR spectrum of compound **19**.

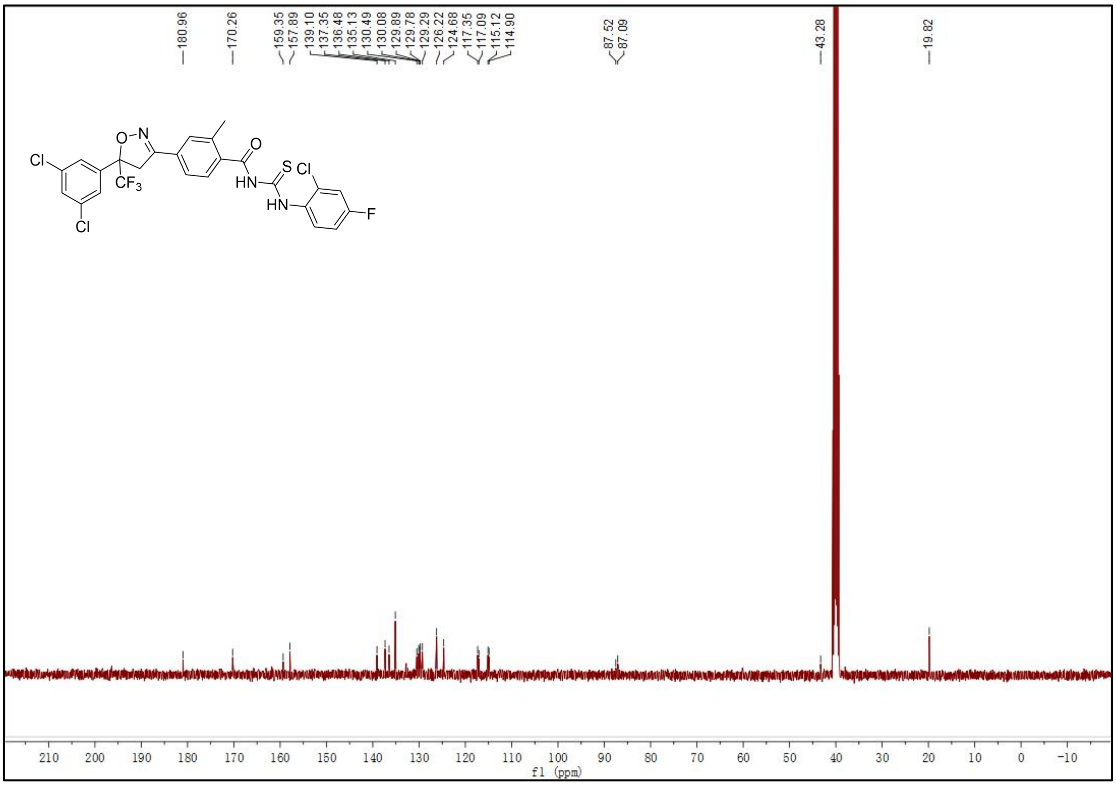

Figure 56. ^{13}C NMR spectrum of compound **19**.

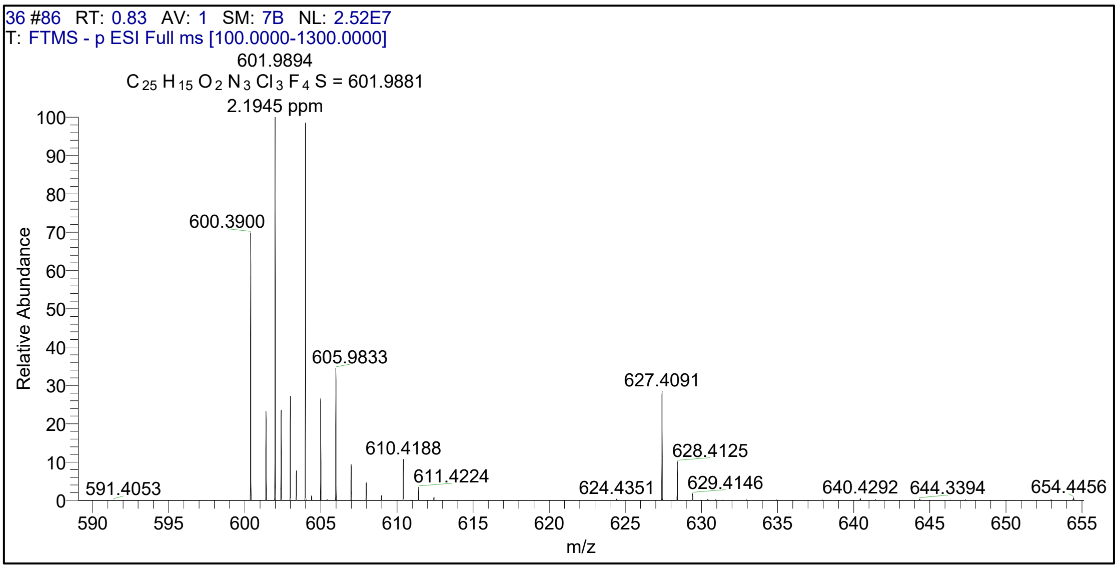

Figure 57. HRMS(ESI) of compound **19.**

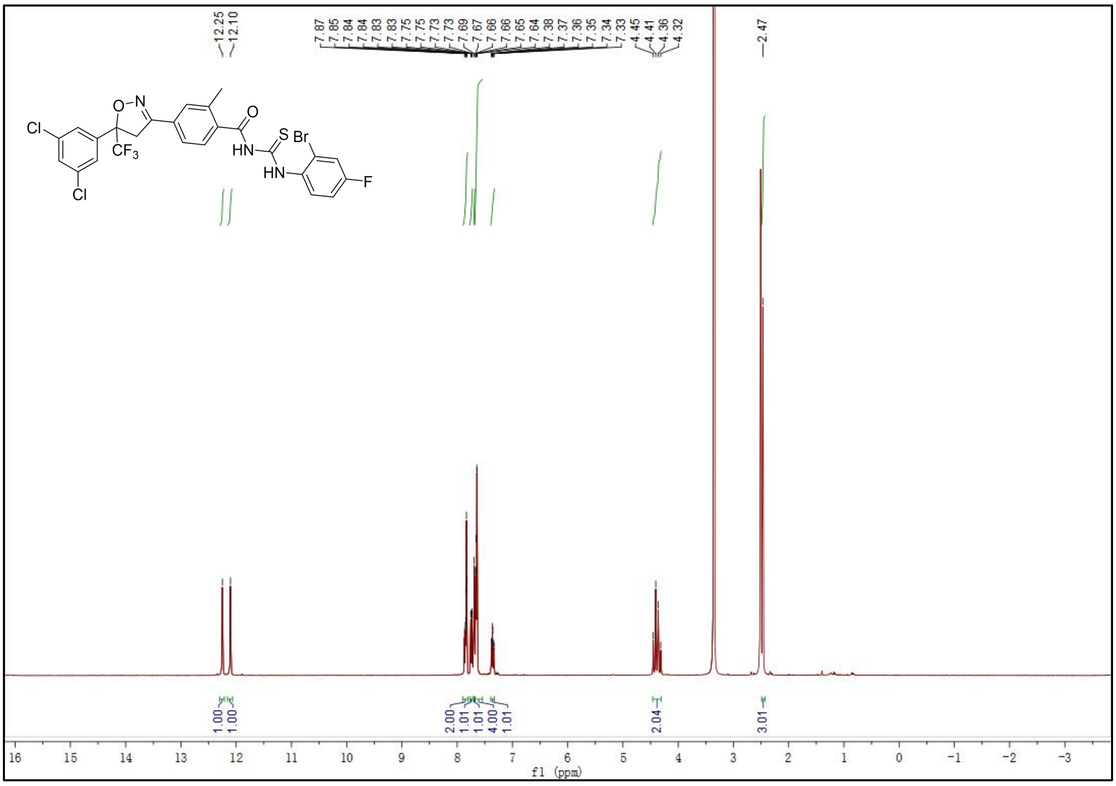

Figure 58. ^{1}H NMR spectrum of compound **20**.

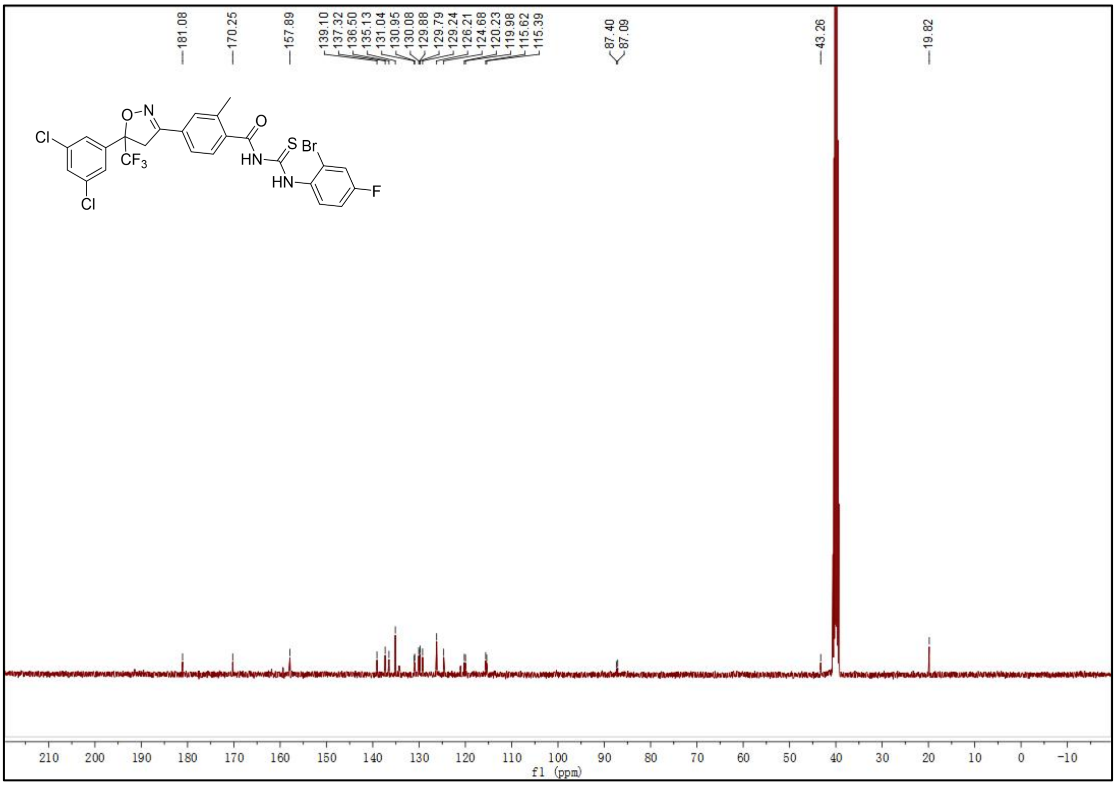

Figure 59. ^{13}C NMR spectrum of compound **20**.

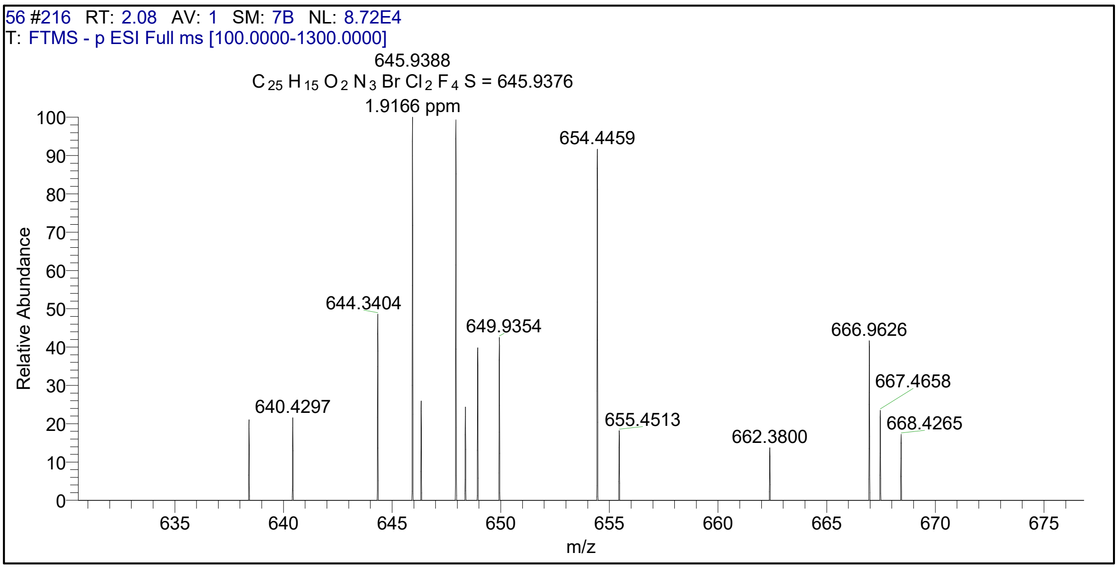

Figure 60. HRMS(ESI) of compound **20.**

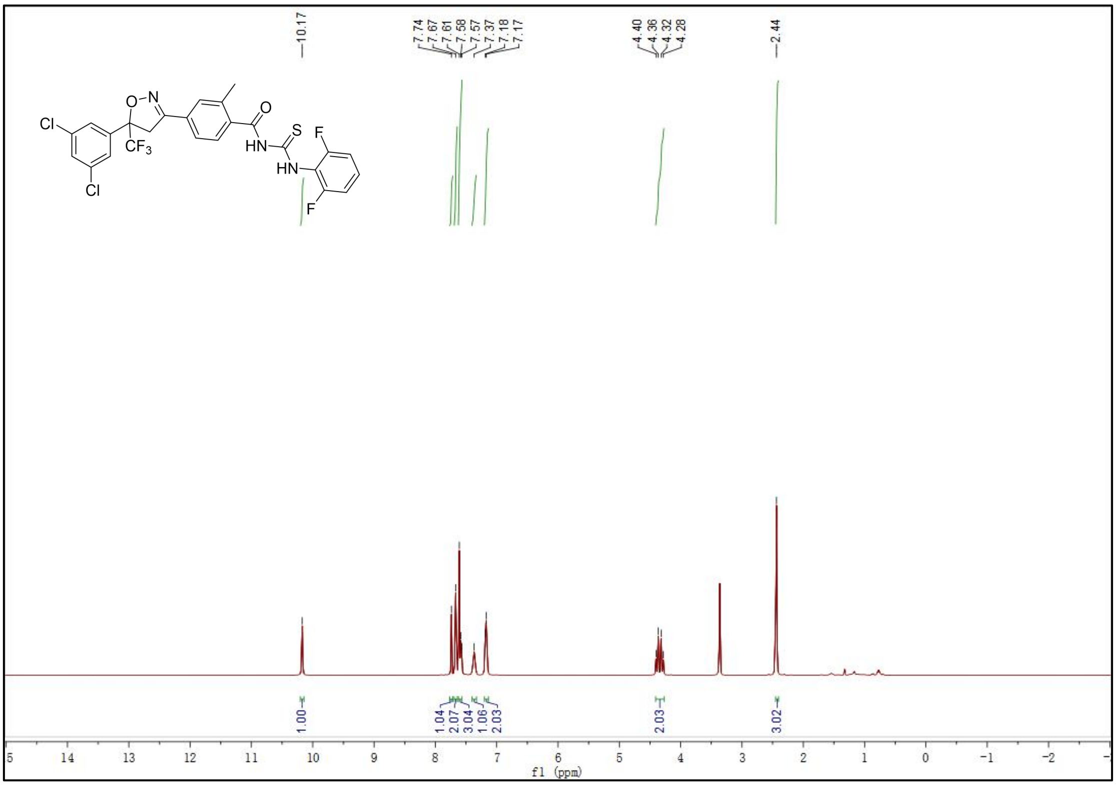

Figure 61. ^{1}H NMR spectrum of compound **21**.

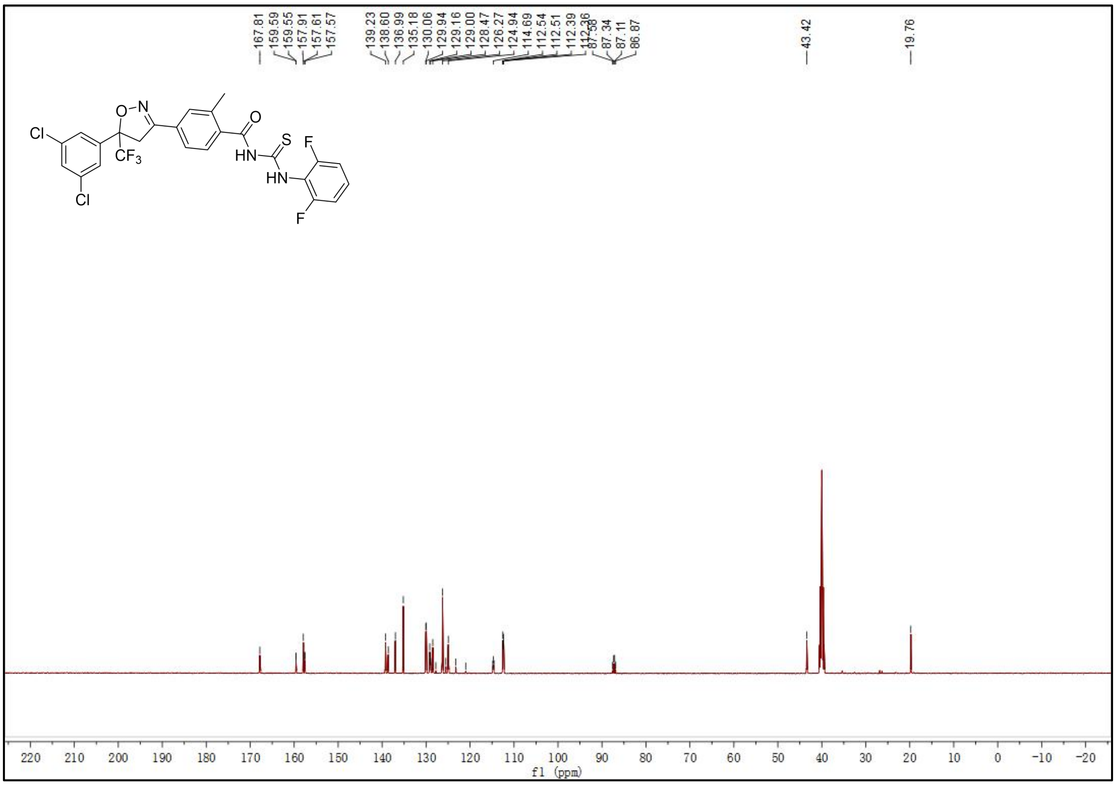

Figure 62. ^{13}C NMR spectrum of compound **21**.

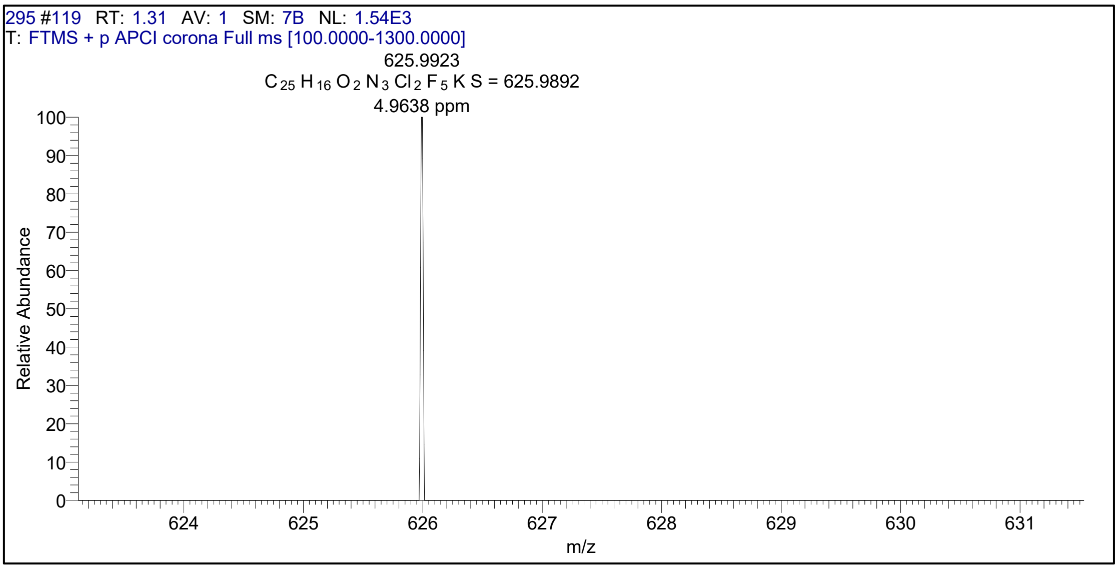

Figure 63. HRMS(ESI) of compound **21.**

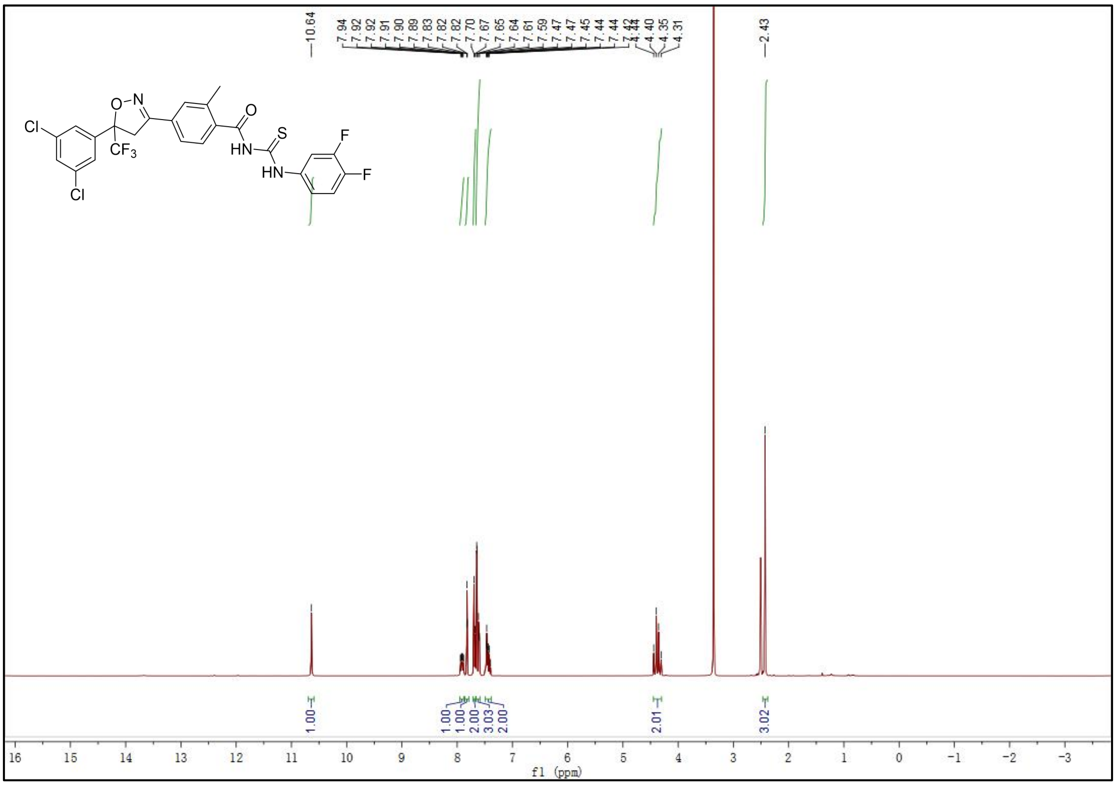

Figure 64. ^{1}H NMR spectrum of compound **22**.

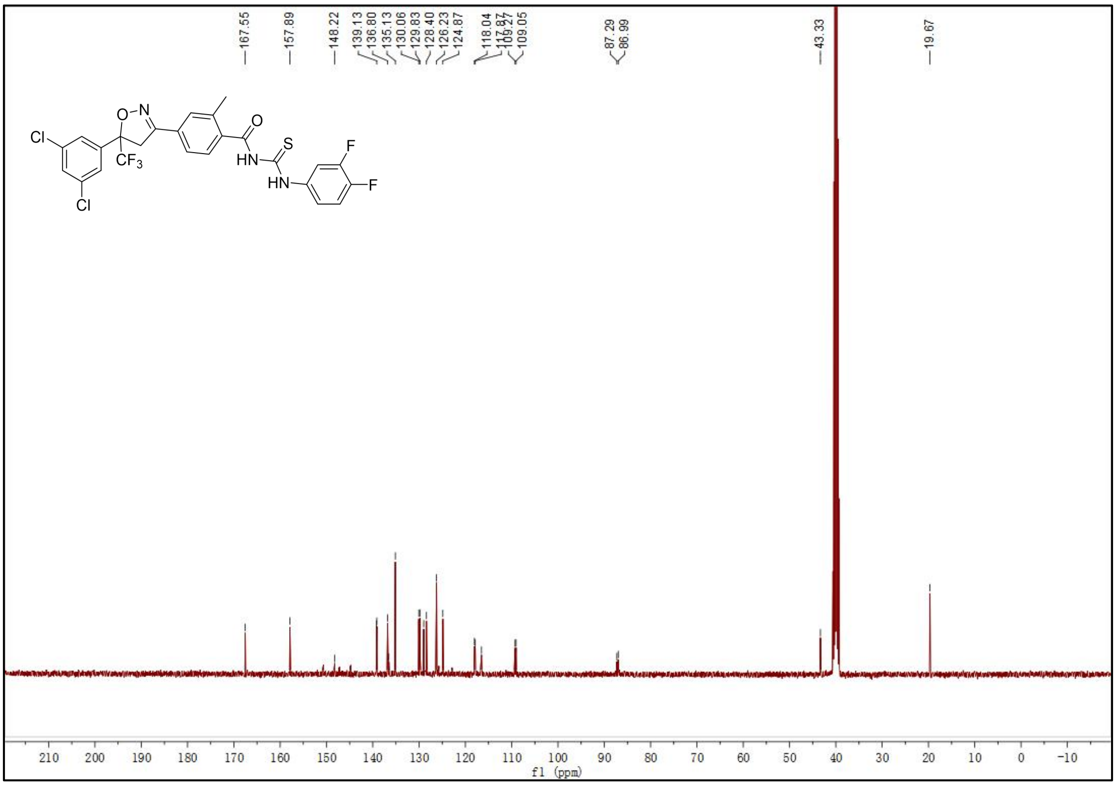

Figure 65. ^{13}C NMR spectrum of compound **22**.

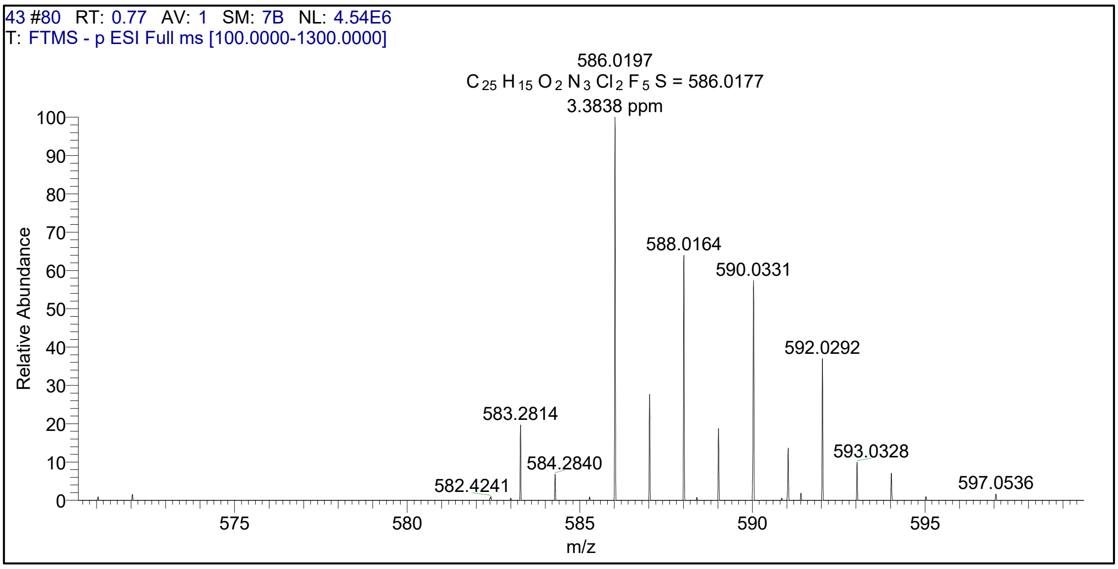

Figure 66. HRMS(ESI) of compound **22.**

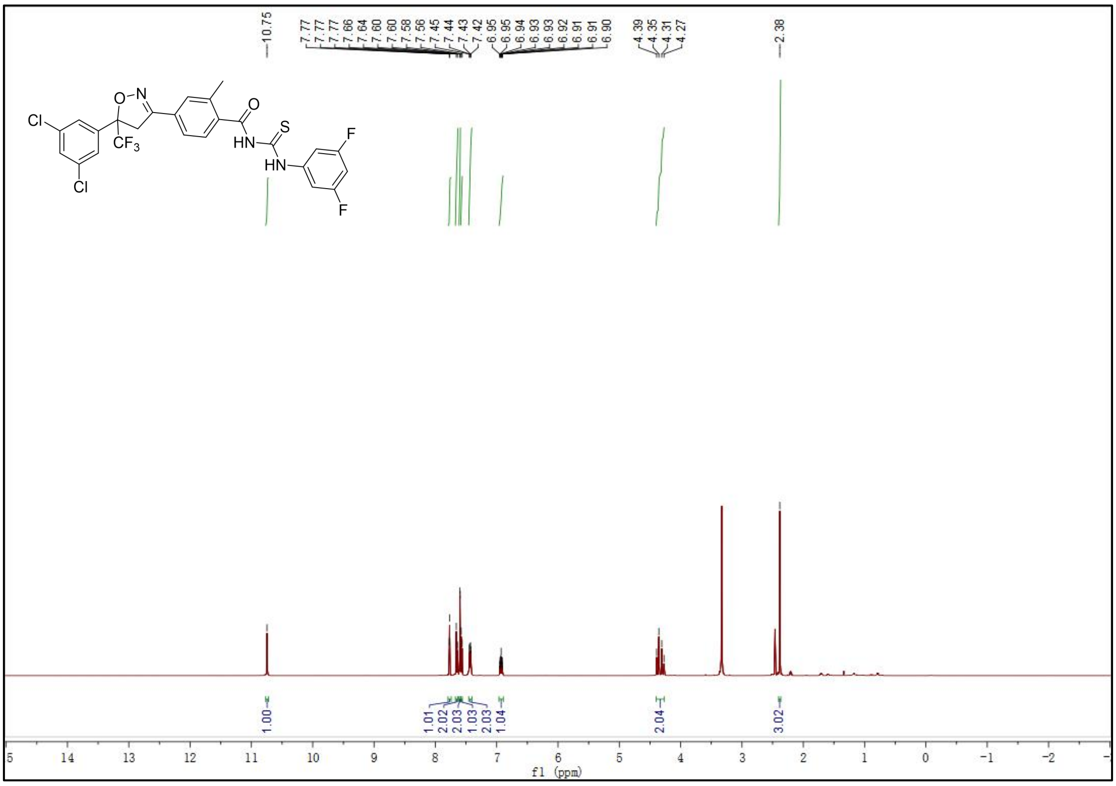

Figure 67. ^{1}H NMR spectrum of compound **23**.

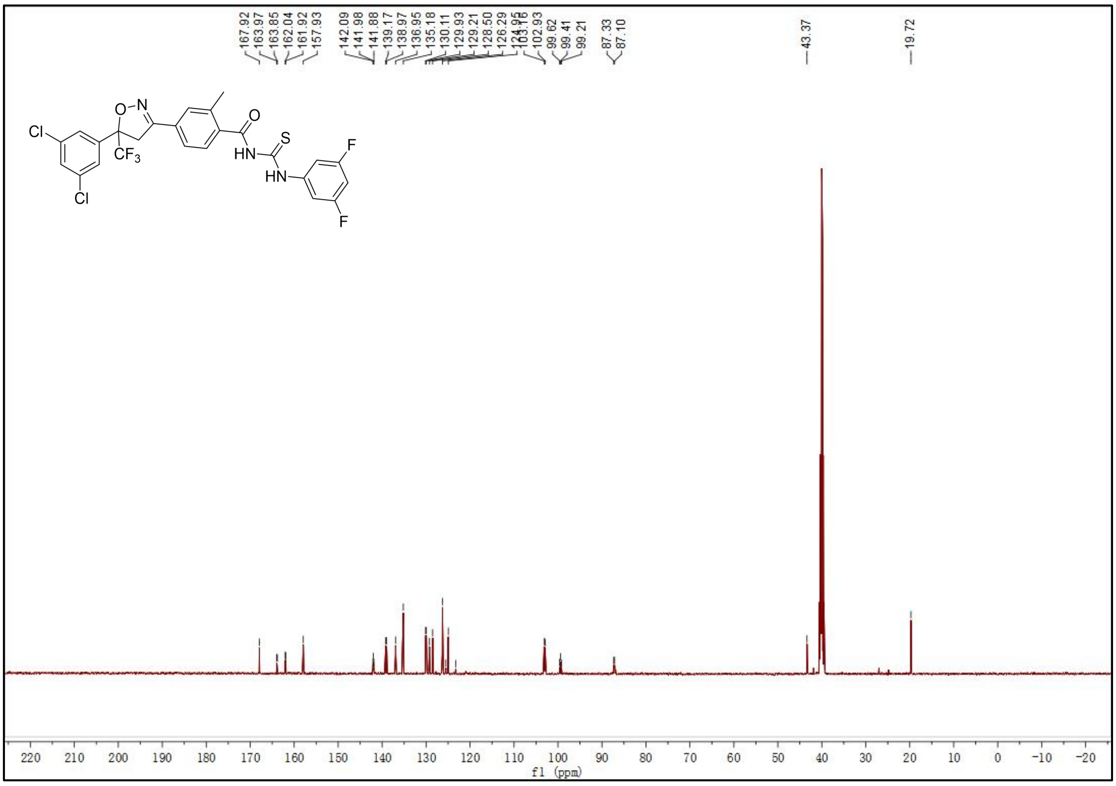

Figure 68. ^{13}C NMR spectrum of compound **23**.

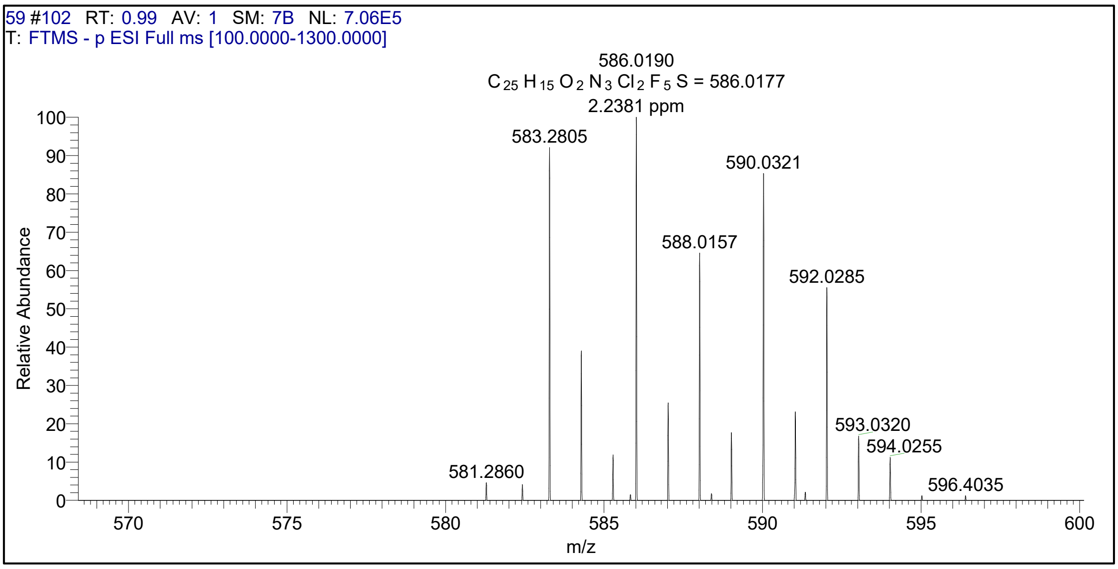

Figure 69. HRMS(ESI) of compound **23.**

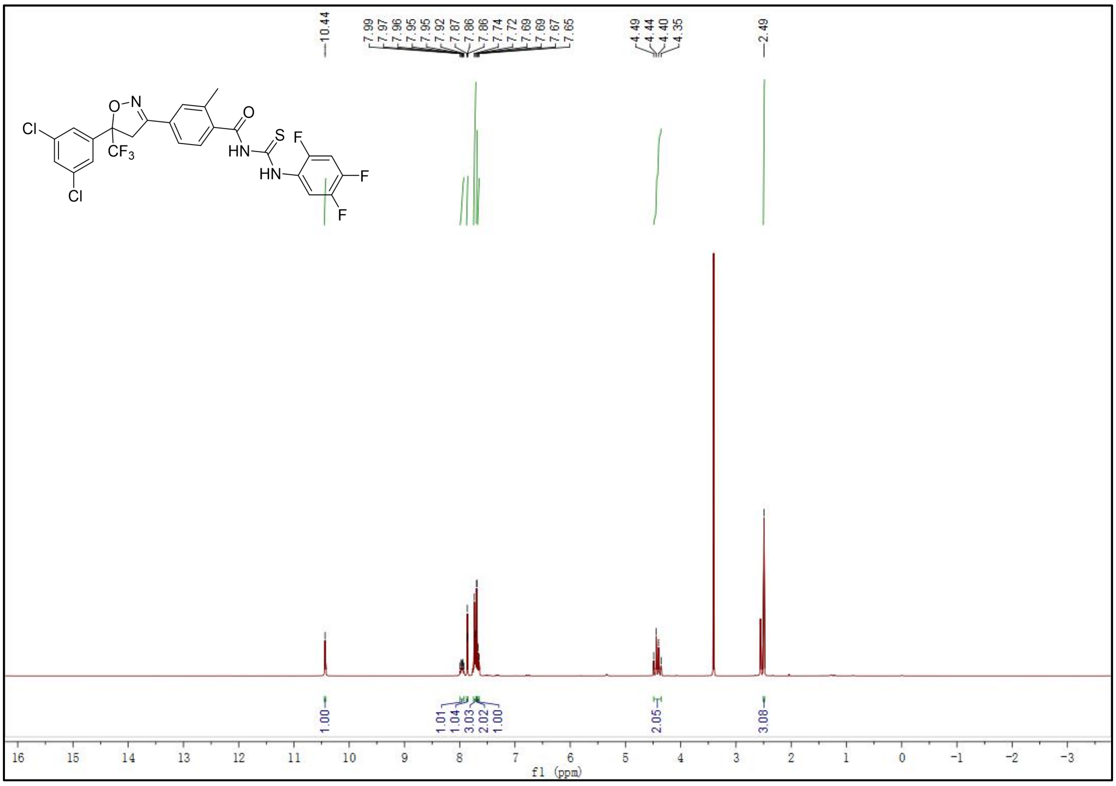

Figure 70. ^{1}H NMR spectrum of compound **24**.

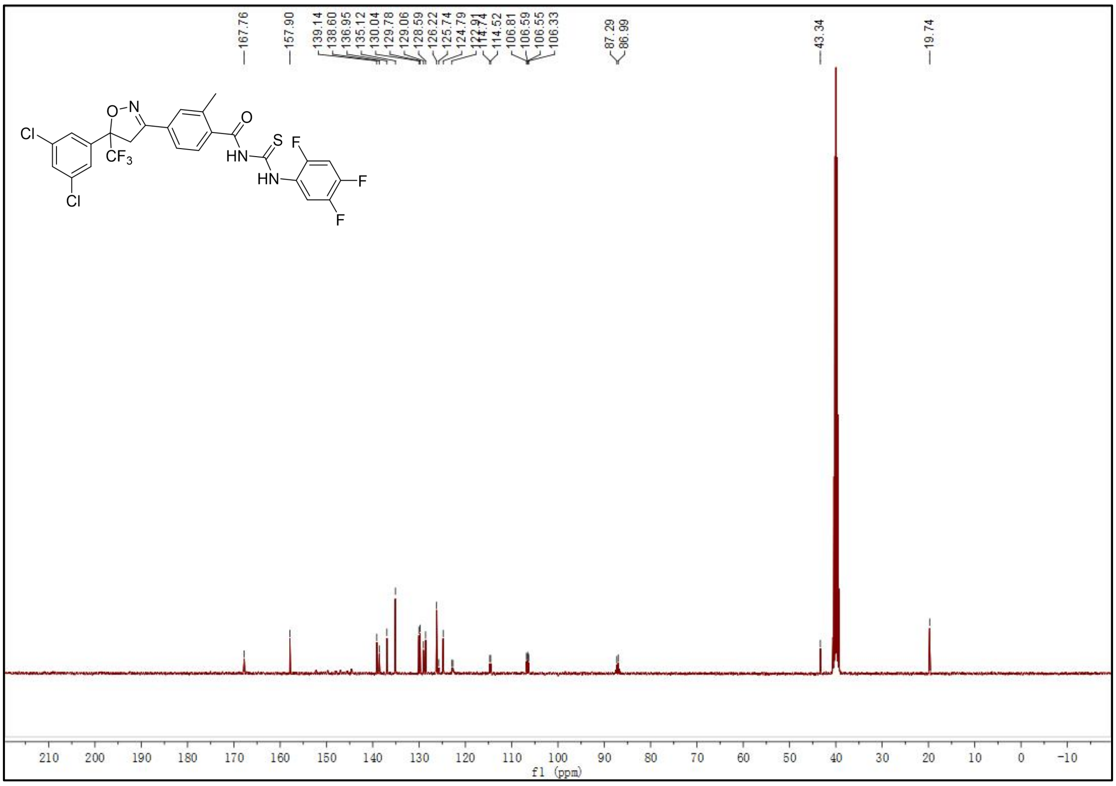

Figure 71. ^{13}C NMR spectrum of compound **24**.

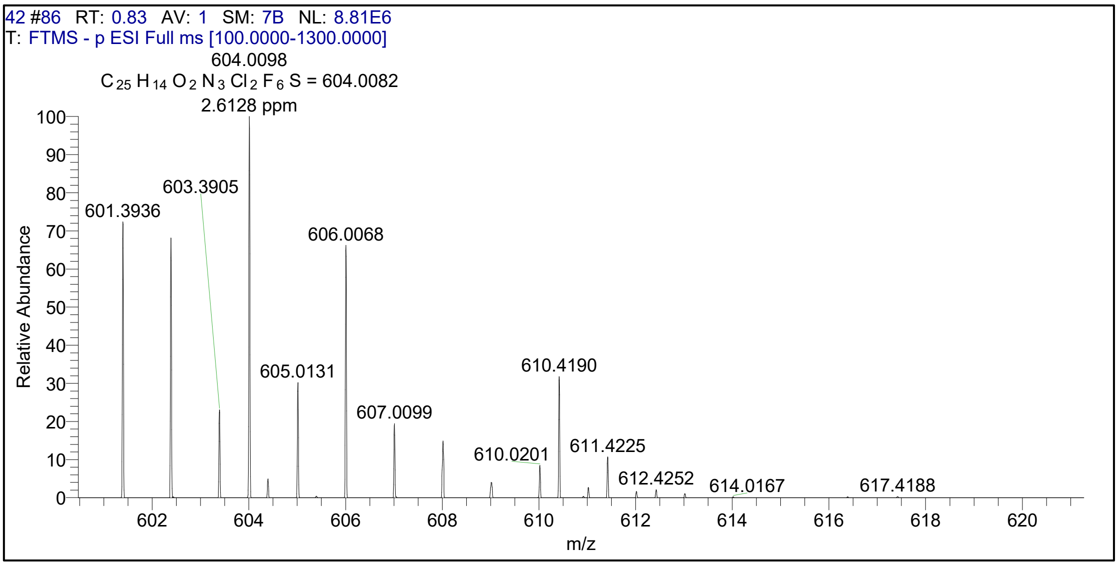

Figure 72. HRMS(ESI) of compound **24.**

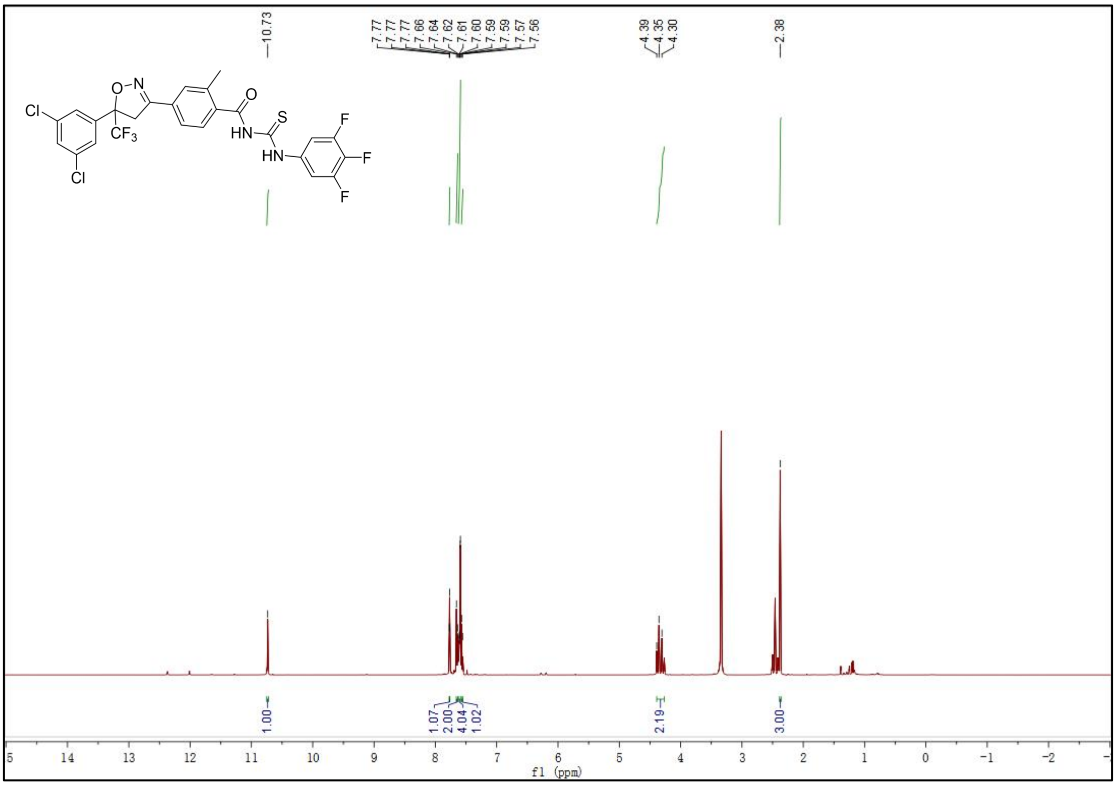

Figure 73. ^{1}H NMR spectrum of compound **25**.

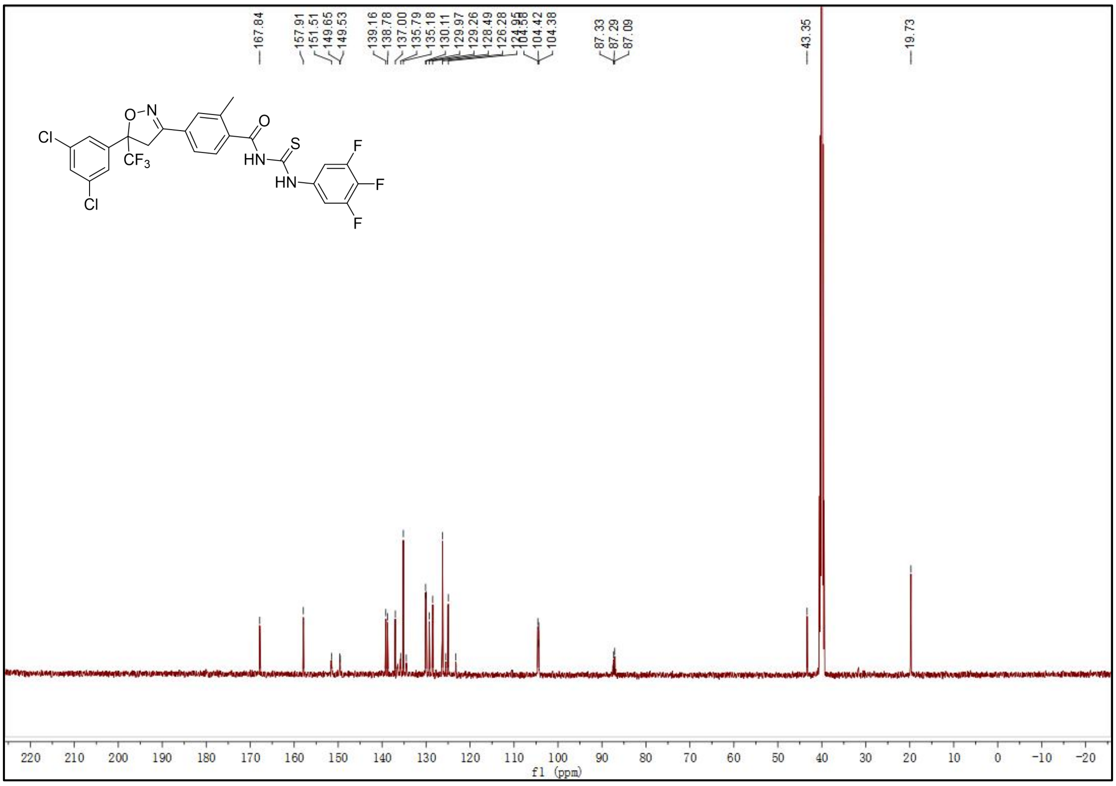

Figure 74. ^{13}C NMR spectrum of compound **25**.

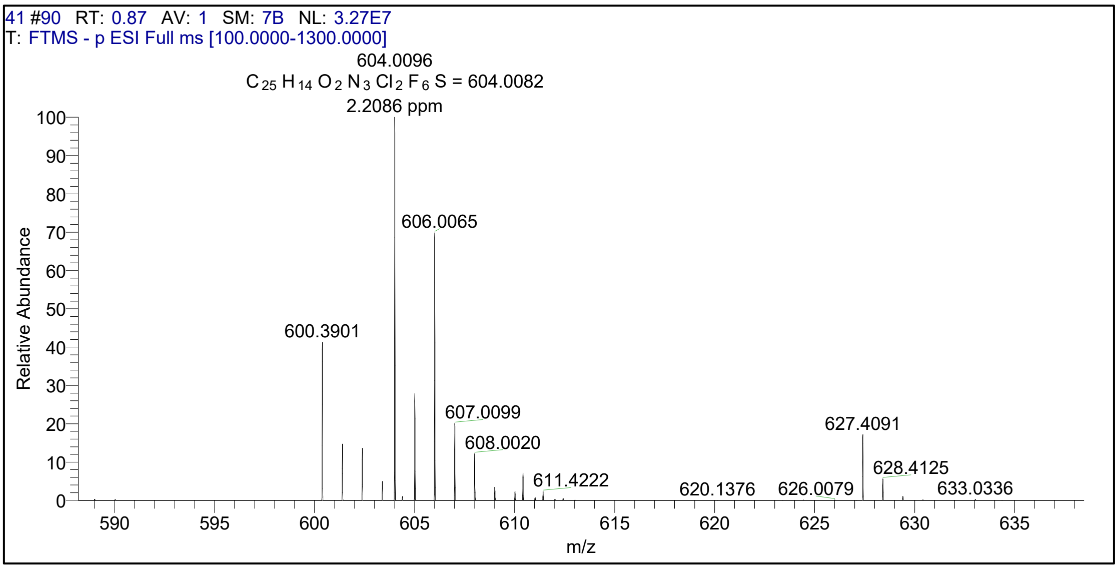

Figure 75. HRMS(ESI) of compound **25.**

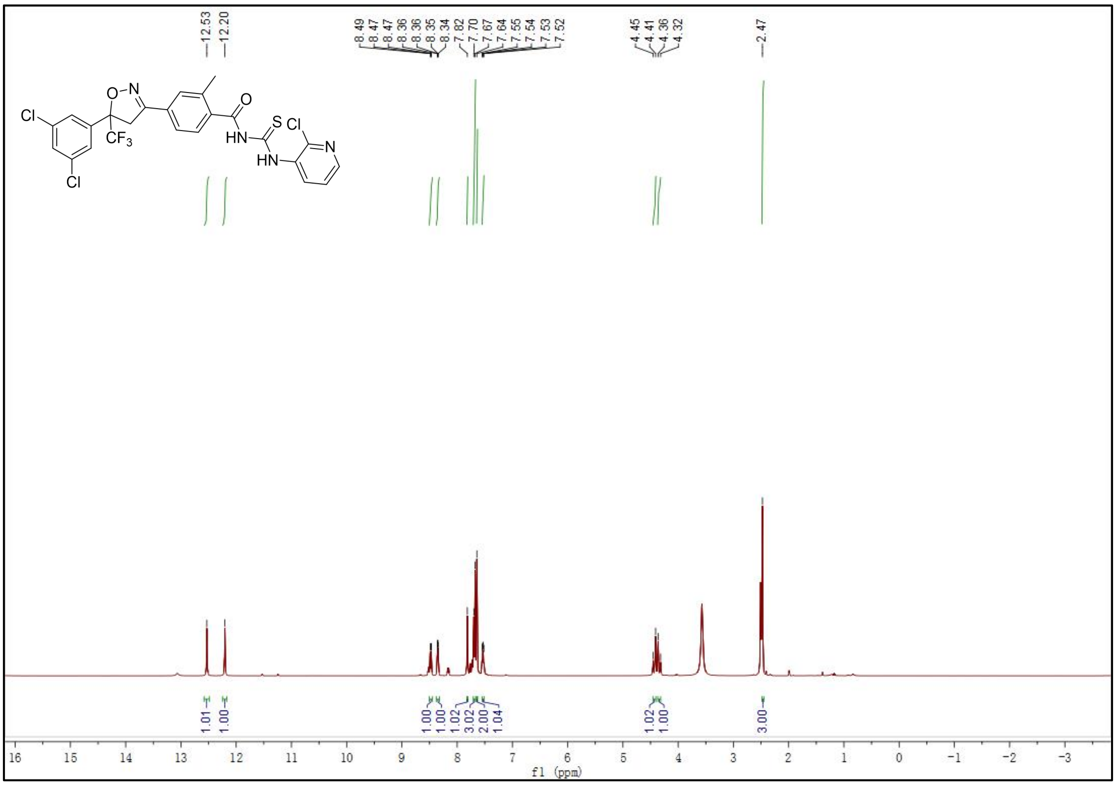

Figure 76. ^{1}H NMR spectrum of compound **26**.

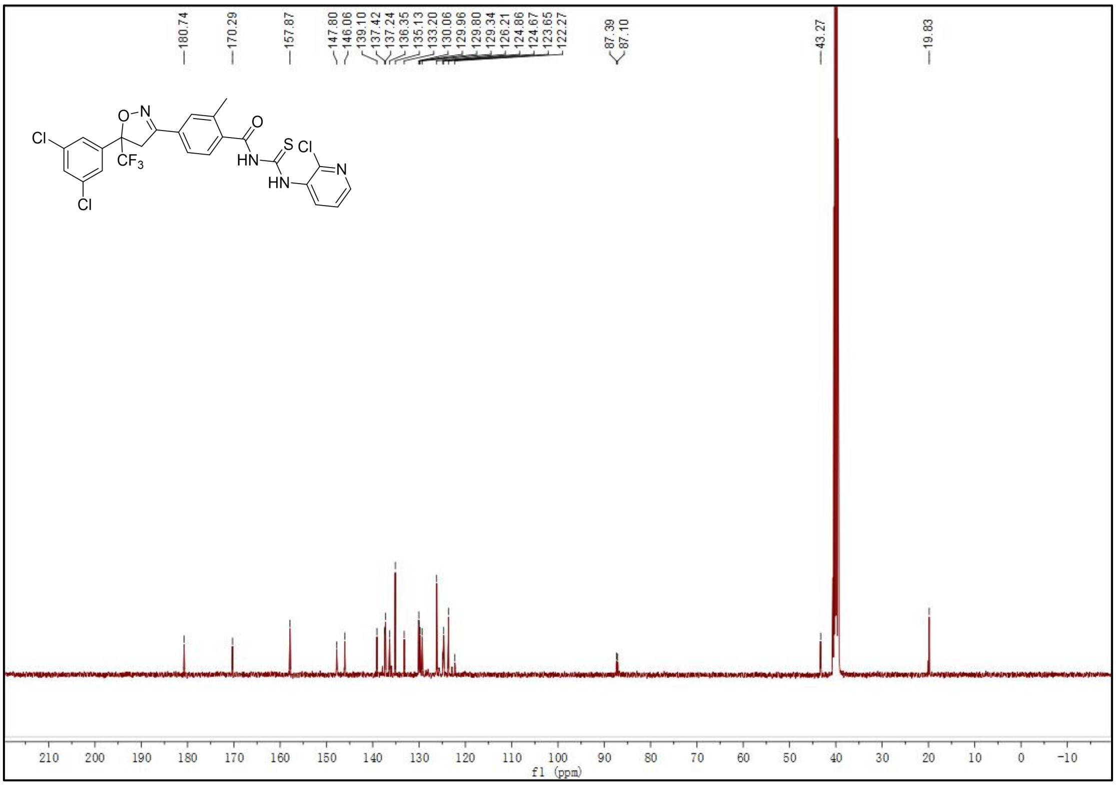

Figure 77. ^{13}C NMR spectrum of compound **26**.

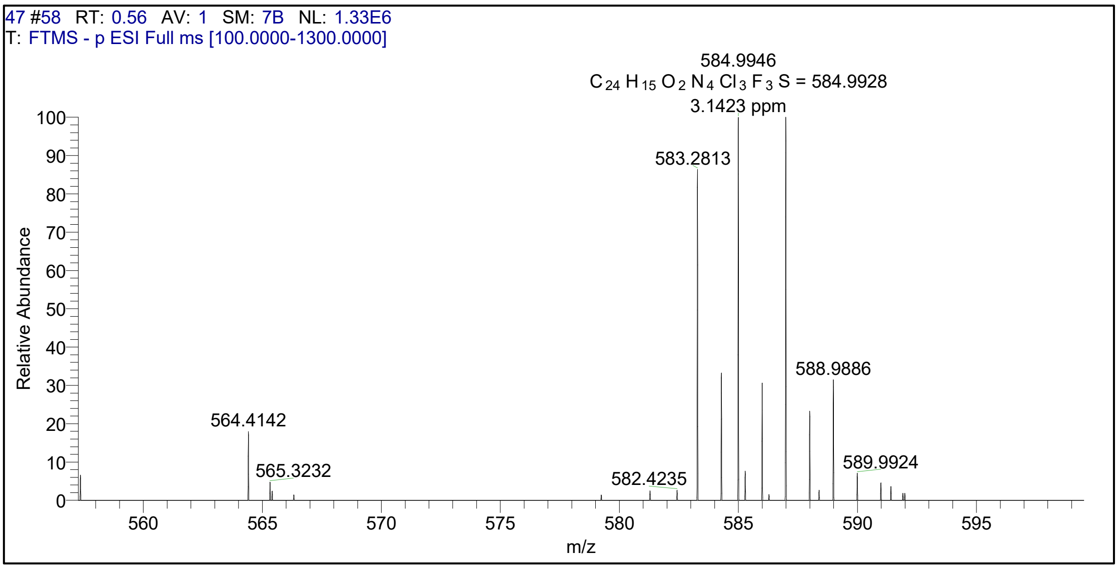

Figure 78. HRMS(ESI) of compound **26.**

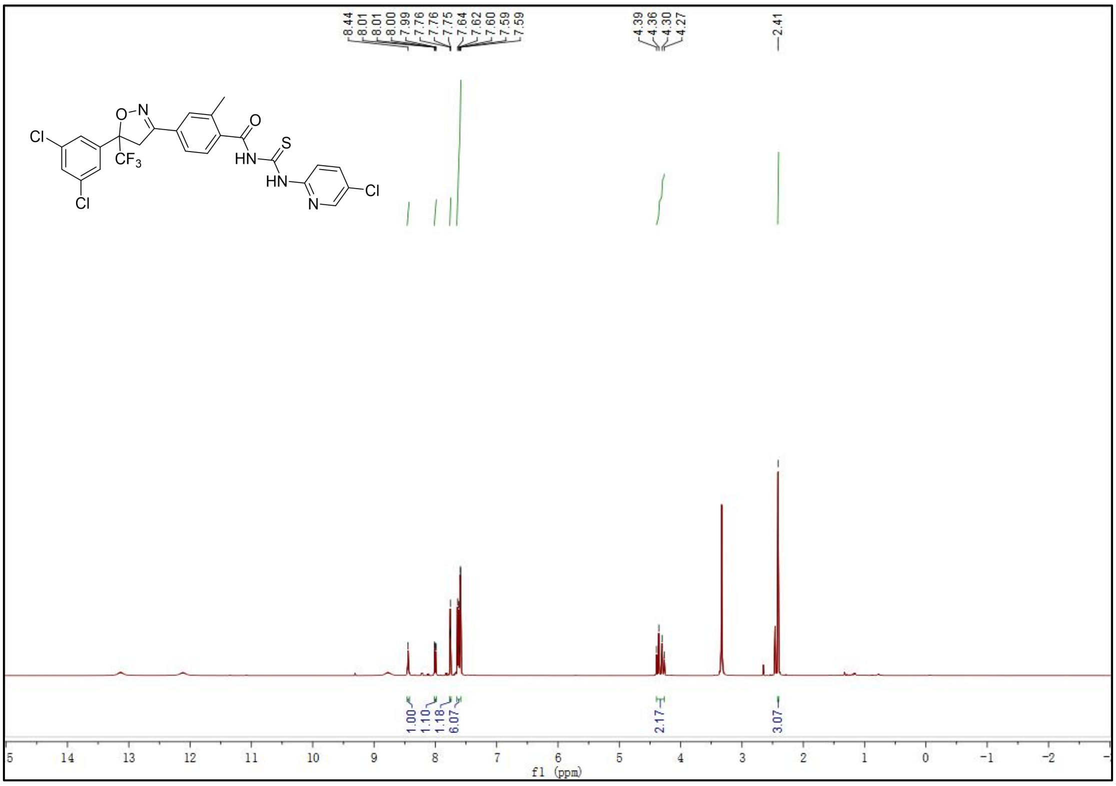

Figure 79. ^{1}H NMR spectrum of compound **27**.

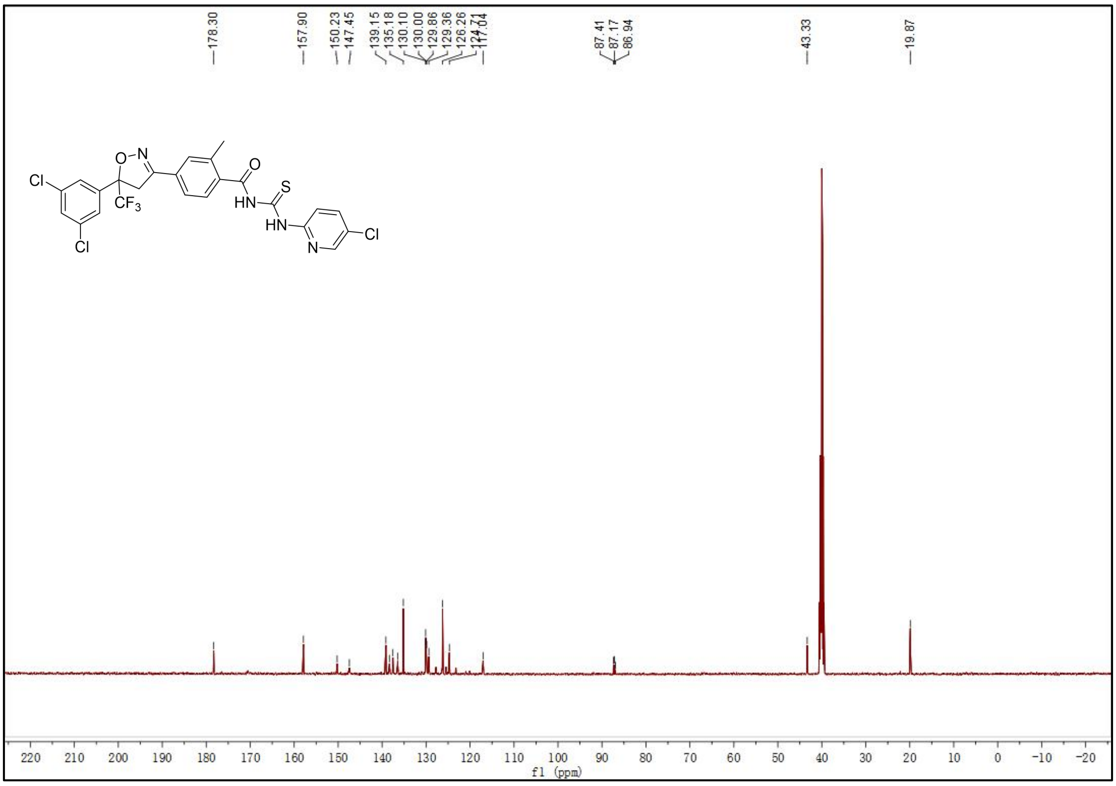

Figure 80. ^{13}C NMR spectrum of compound **27**.

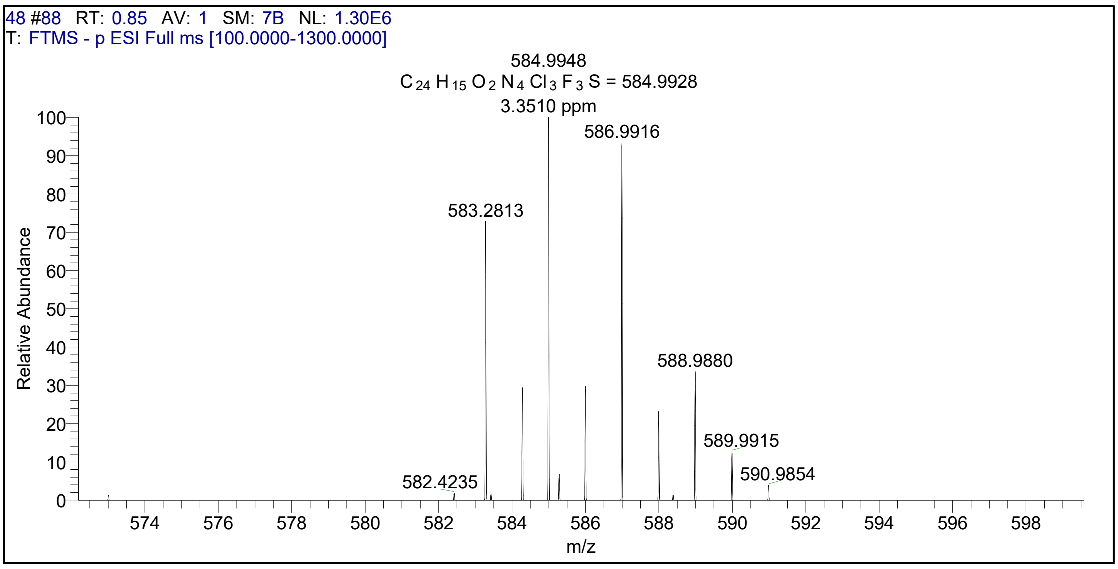

Figure 81. HRMS(ESI) of compound **27.**

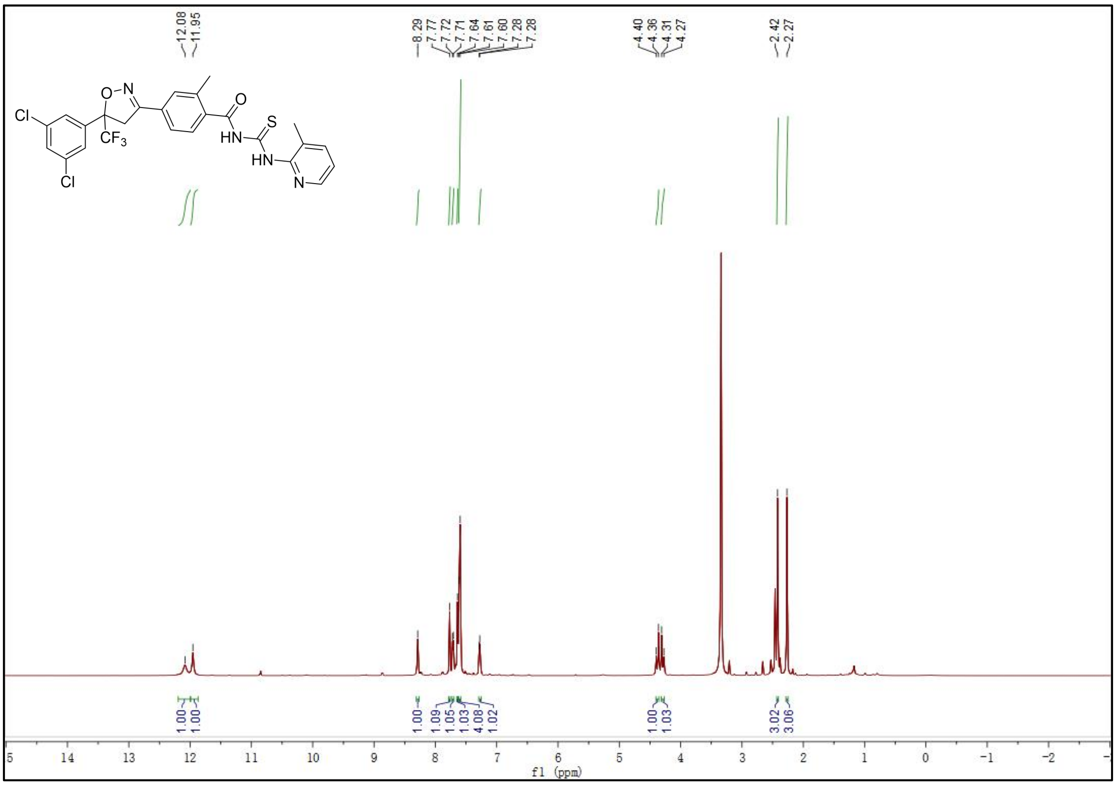

Figure 82. ^{1}H NMR spectrum of compound **28**.

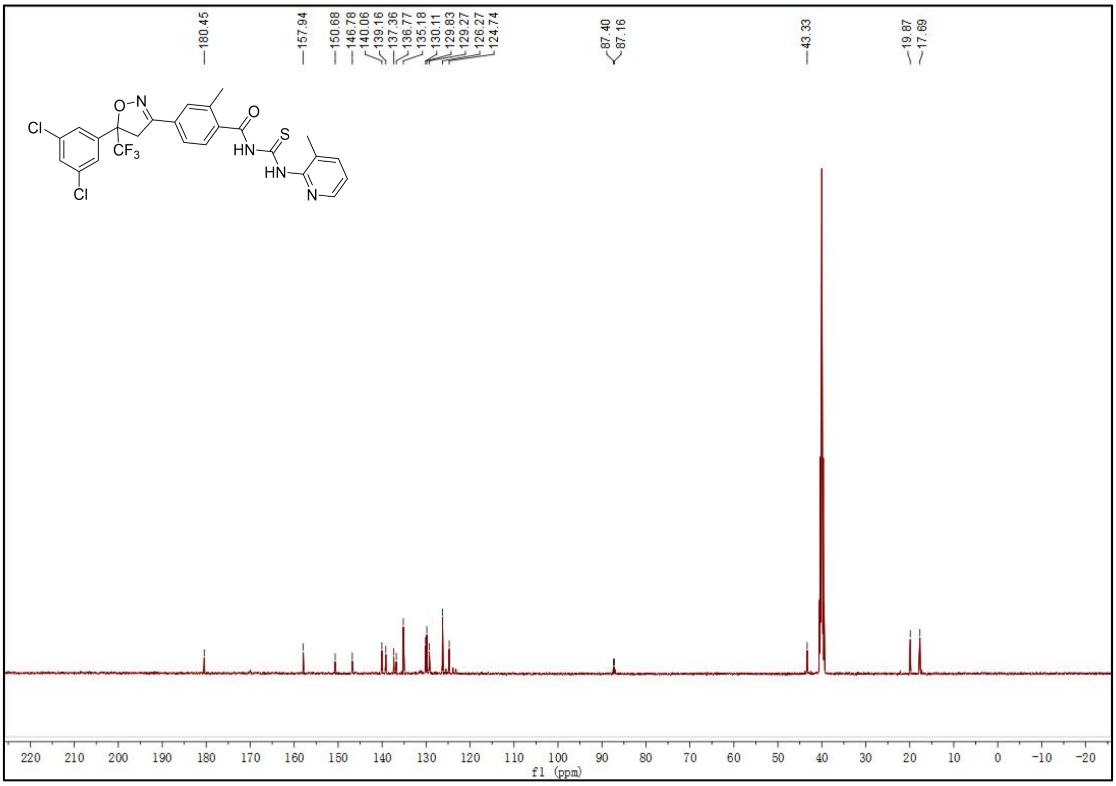

Figure 83. ^{13}C NMR spectrum of compound **28**.

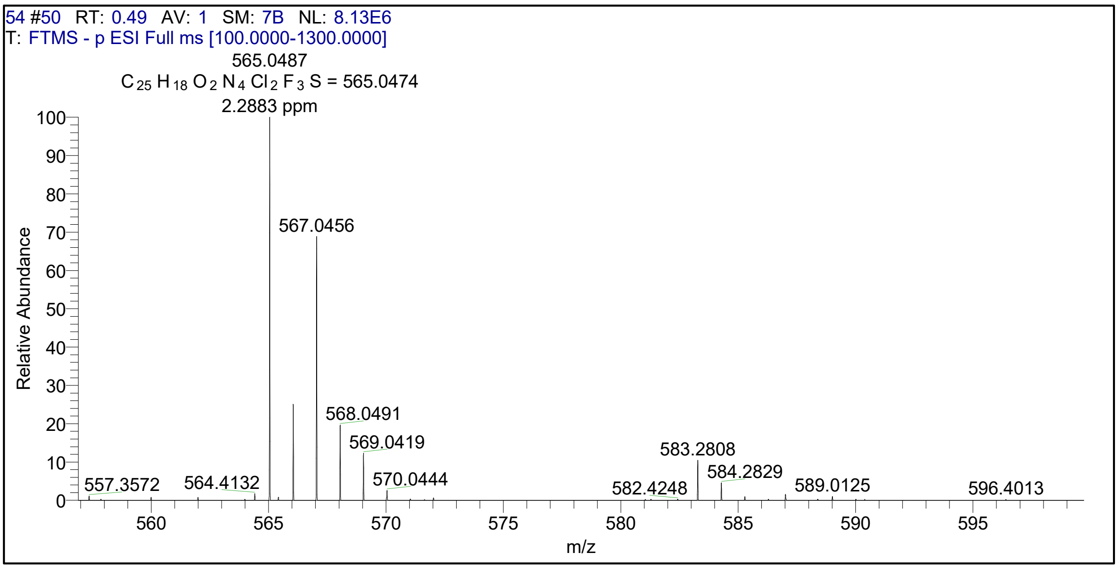

Figure 84. HRMS(ESI) of compound **28.**

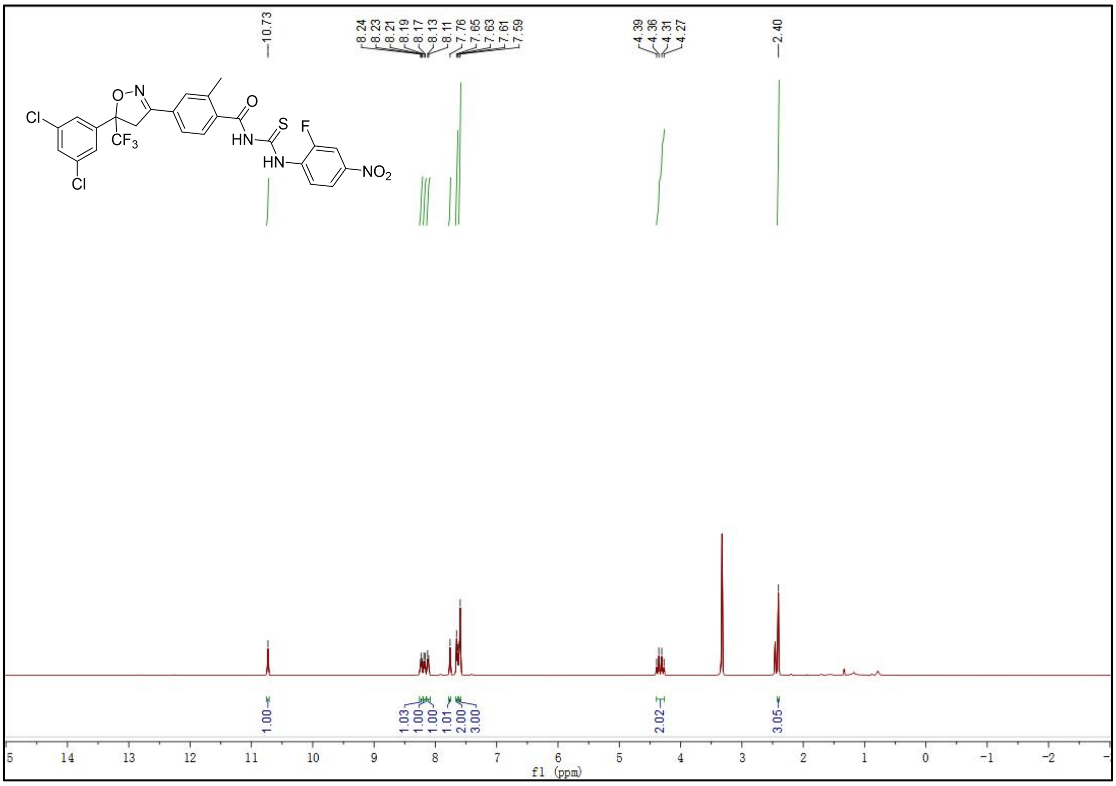

Figure 85. ^{1}H NMR spectrum of compound **29**.

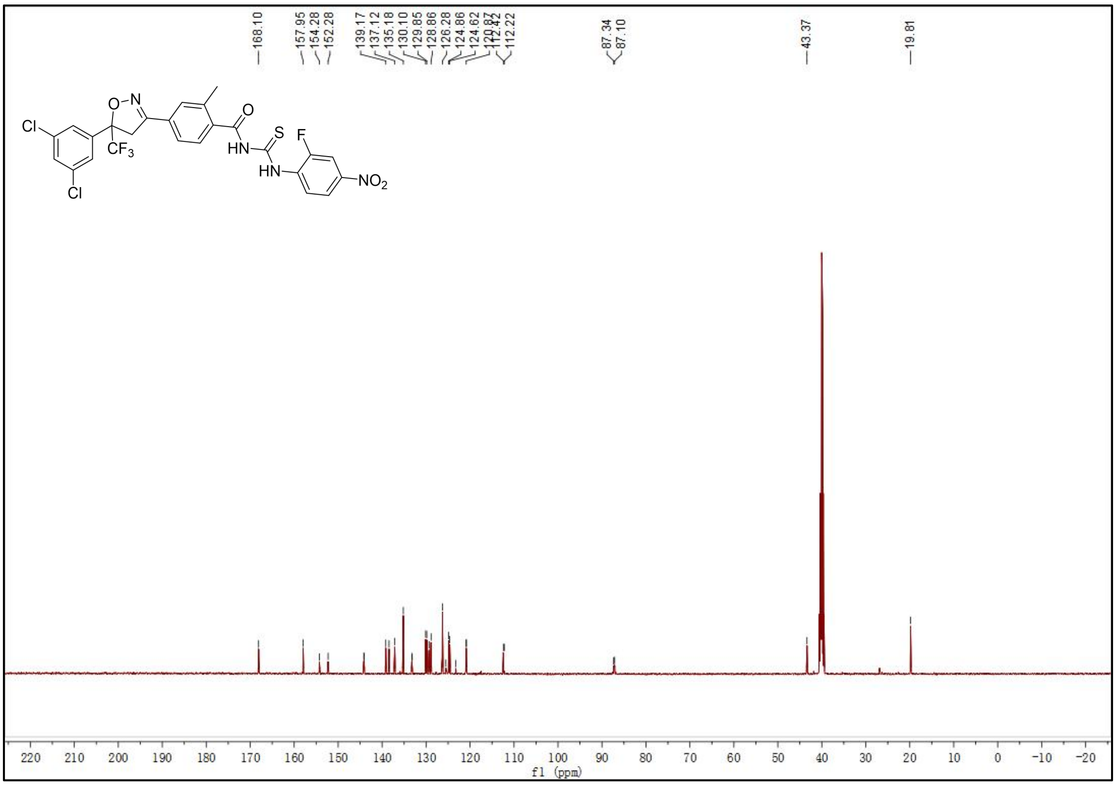

Figure 86. ^{13}C NMR spectrum of compound **29**.

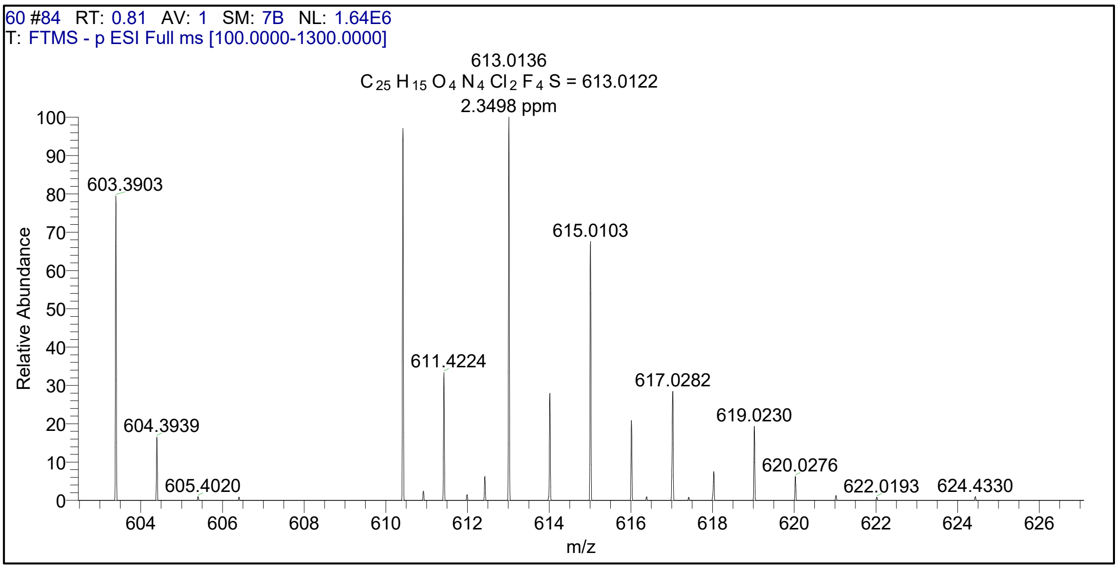

Figure 87. HRMS(ESI) of compound **29.**

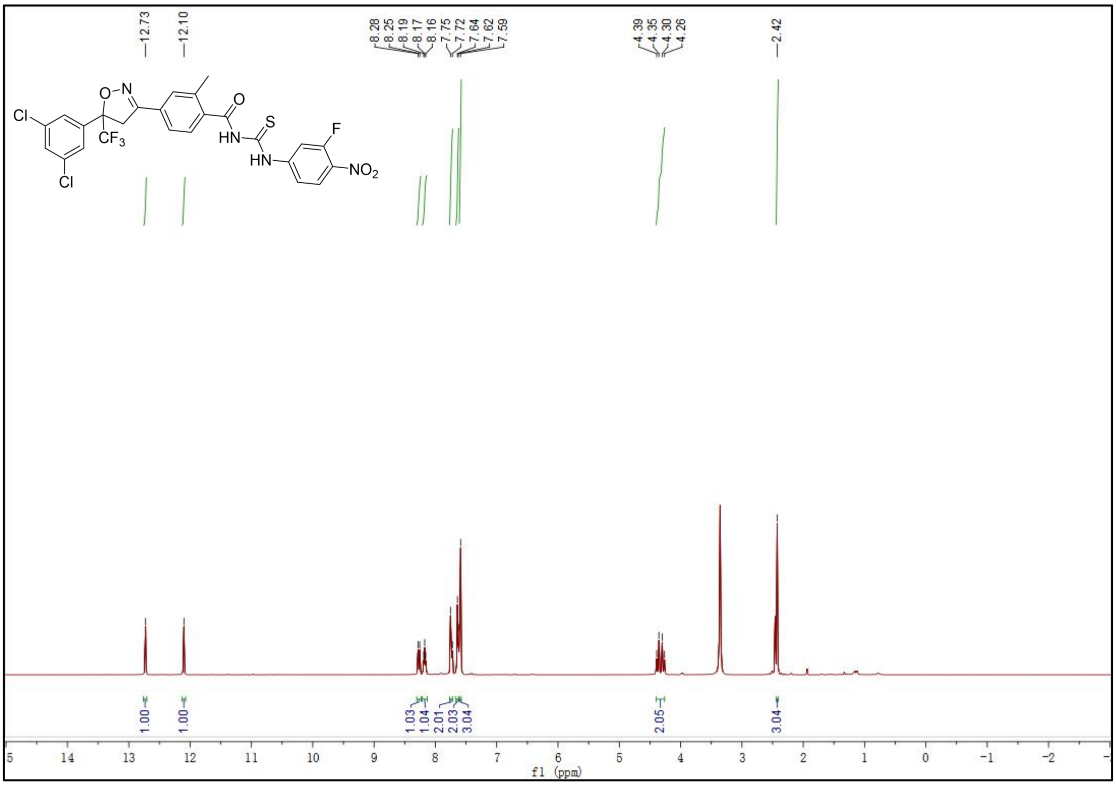

Figure 88. ¹H NMR spectrum of compound **30**.

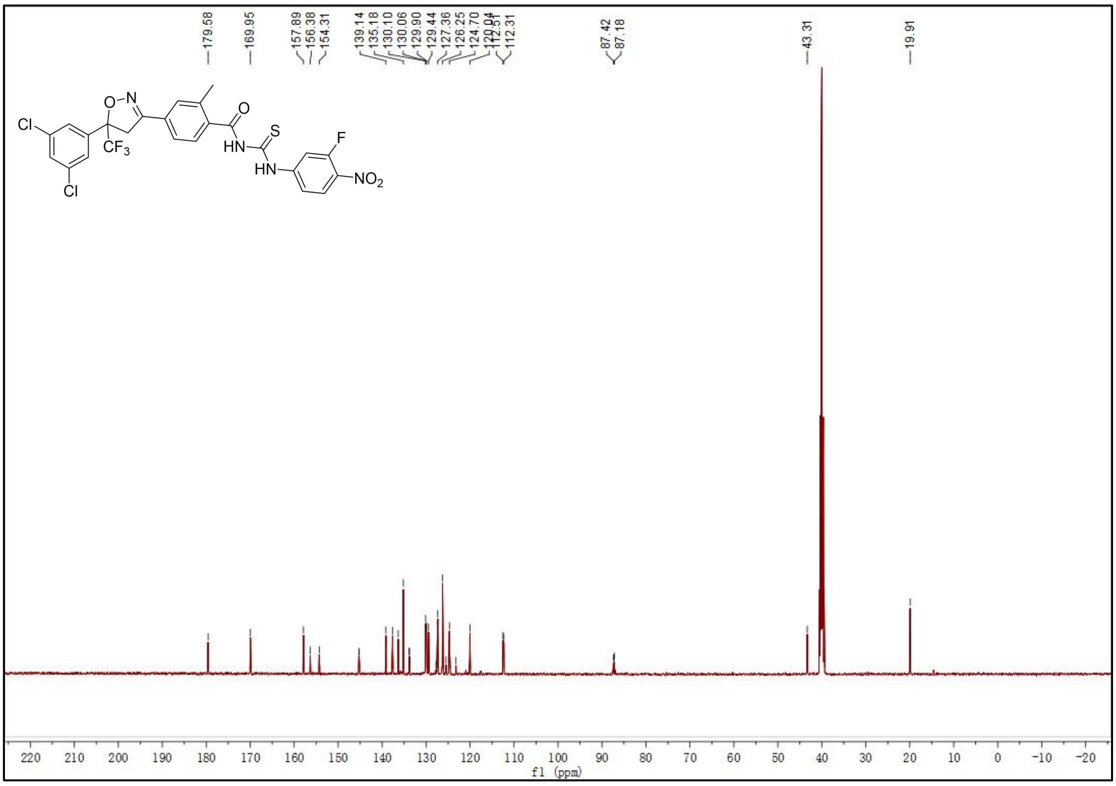

Figure 89. ^{13}C NMR spectrum of compound **30**.

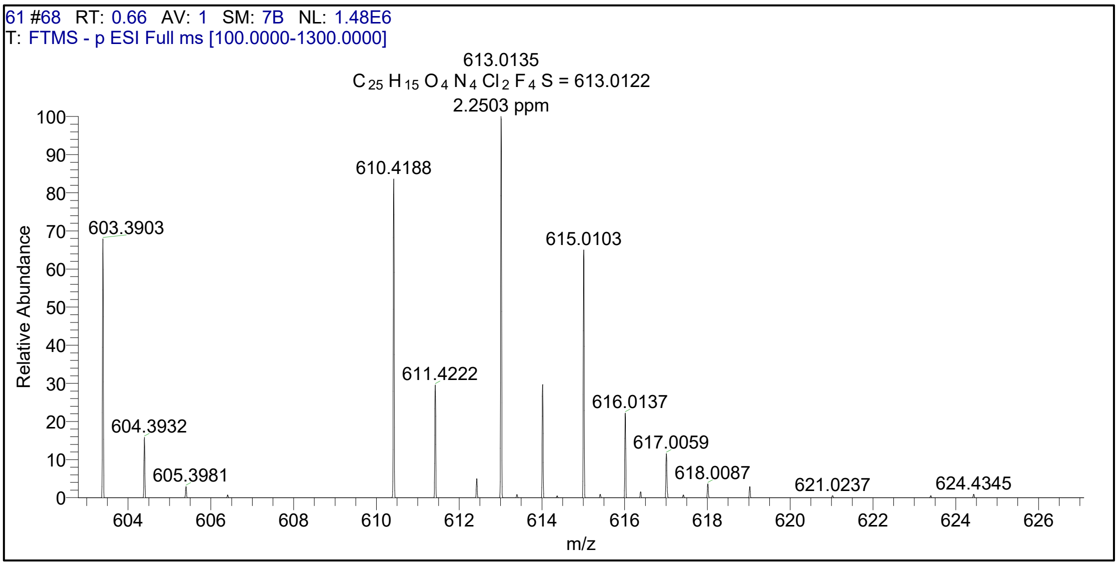

Figure 90. HRMS(ESI) of compound **30.**

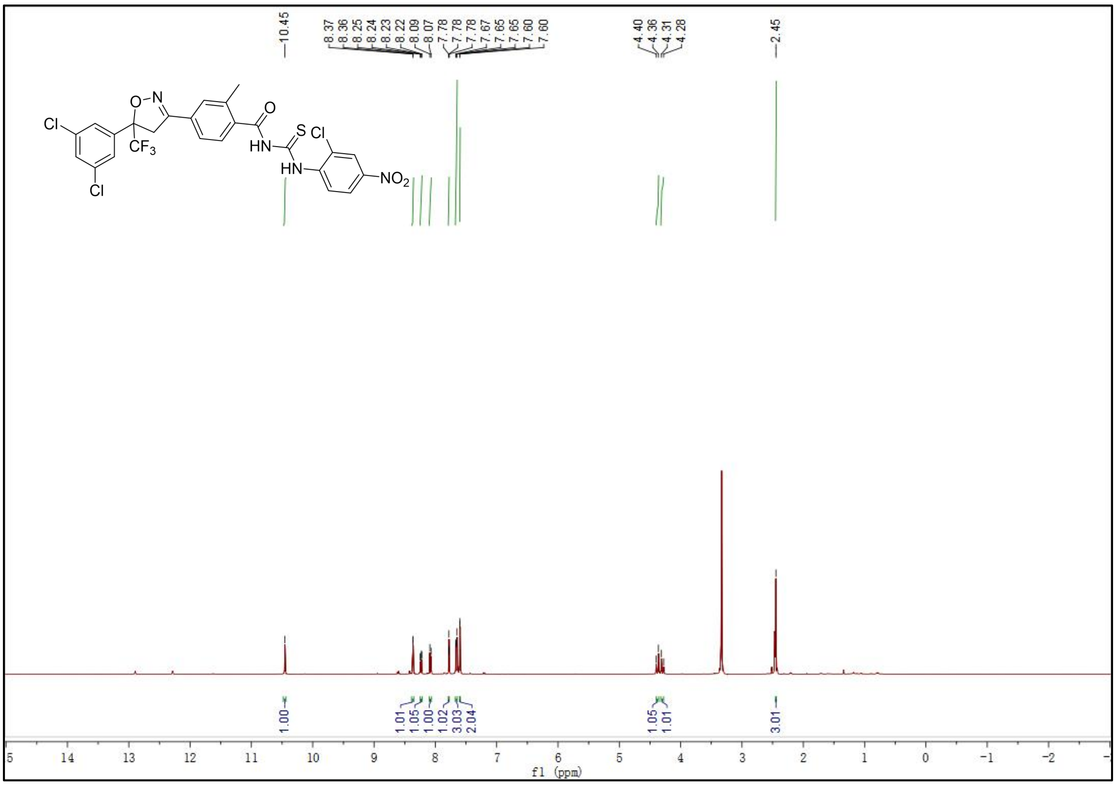

Figure 91. ^{1}H NMR spectrum of compound **31**.

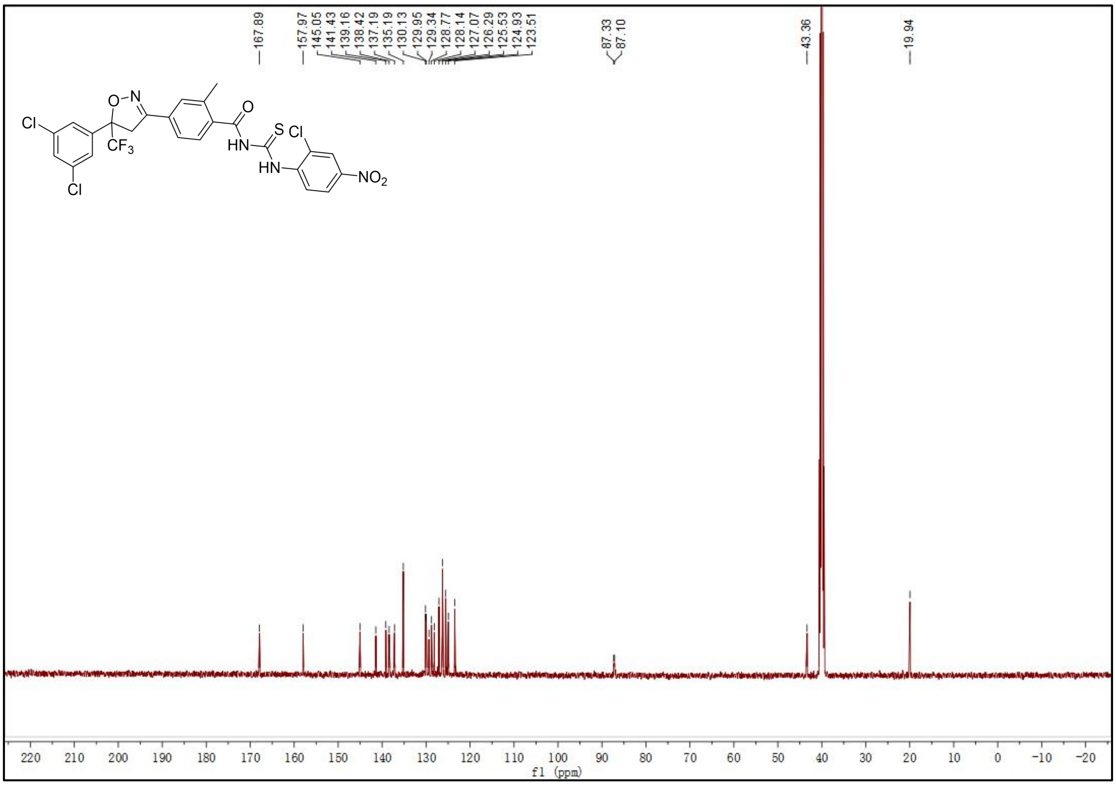

Figure 92. ^{13}C NMR spectrum of compound **31**.

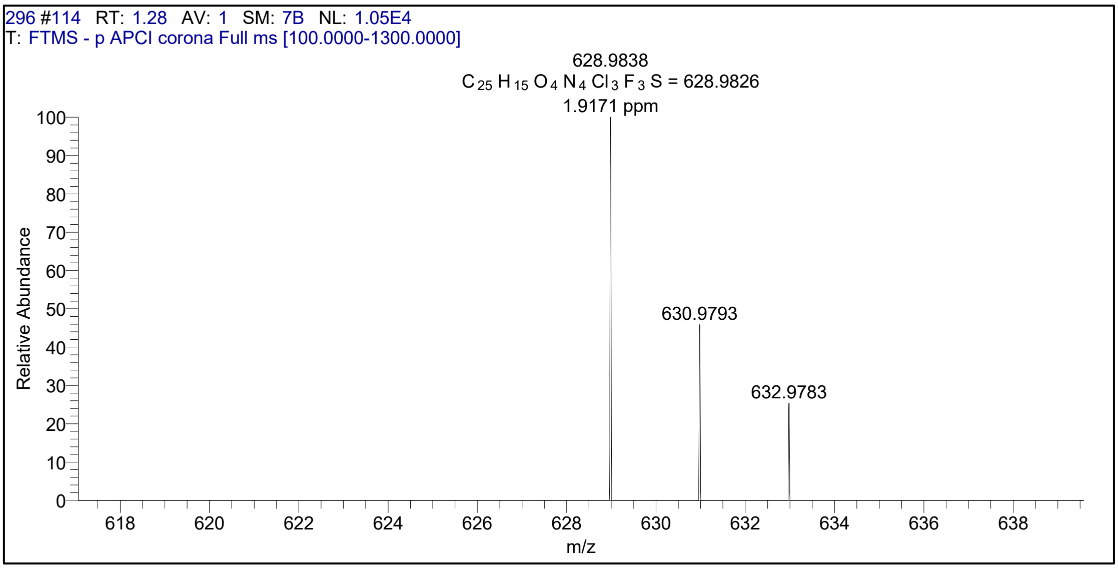

Figure 93. HRMS(ESI) of compound **31.**

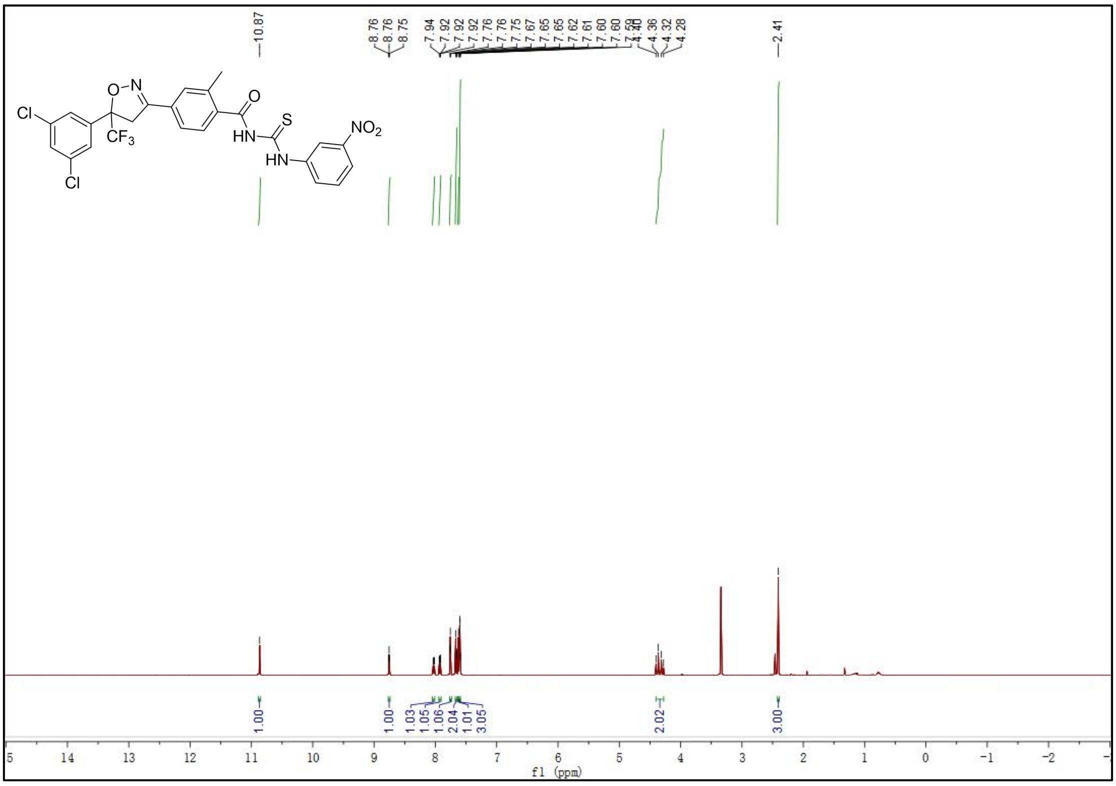

Figure 94. ^{1}H NMR spectrum of compound **32**.

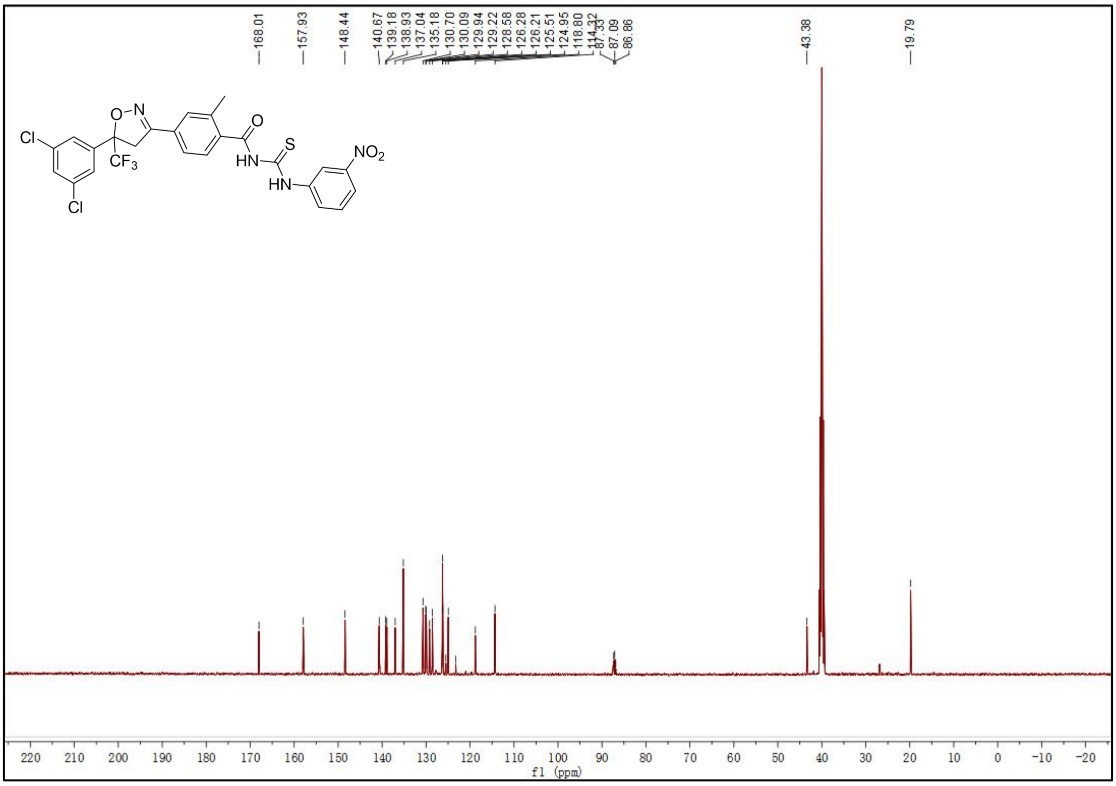

Figure 95. ^{13}C NMR spectrum of compound **32**.

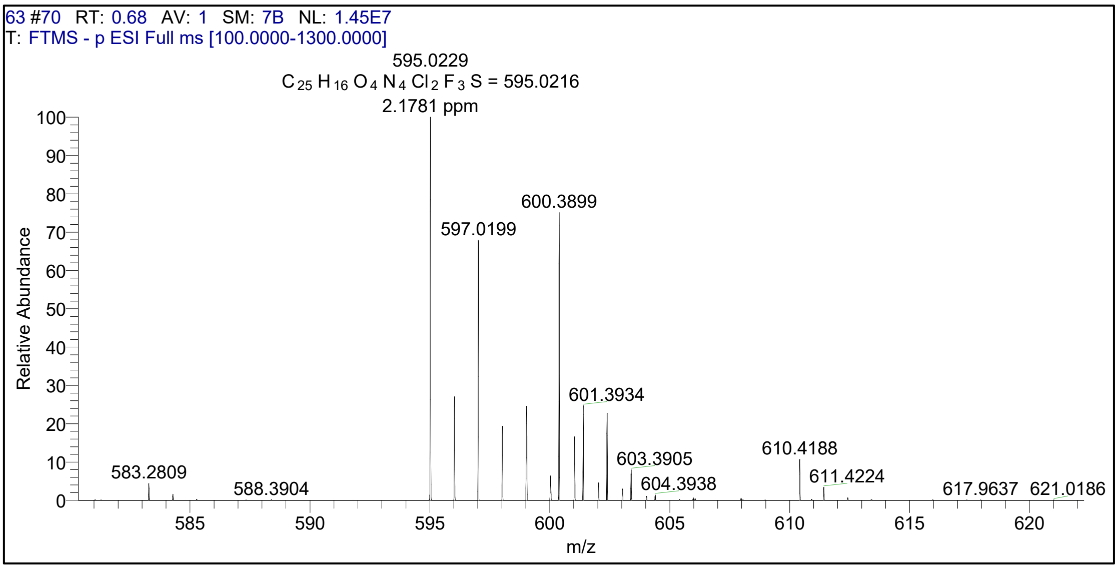

Figure 96. HRMS(ESI) of compound **32.**
